# Supplementary material for: Evaluation of the Growth Assessment Protocol (GAP) for antenatal detection of small for gestational age: The DESiGN cluster randomised trial
Source: PLoS Med. 2022 Jun 21;19(6):e1004004. doi: 10.1371/journal.pmed.1004004 (PMC9212153; doi:10.1371/journal.pmed.1004004)
Supplement: S1 Protocol — (DOCX) [file pmed.1004004.s001.docx]

**The DESiGN Trial**

**DEtection of Small for GestatioNal age fetus (SGA) – a cluster randomised controlled trial to evaluate the effect of the Growth assessment protocol (GAP) programme**

| Version | 8.0 |
| --- | --- |
| Date | 16/08/2018 |
| Sponsor | King’s College London (KCL) |
| IRAS project ID | 180646 |
| Trial Adoption (Portfolio ID) | 20296 |
| Trial registration | [ISRCTN67698474] |
| CTA # | [insert CTA number] |
| NRES # | [15/LO/1632] |

Authorisation: Chief Investigator

| Name | Dr Dharmintra Pasupathy |
| --- | --- |
| Role | Senior Clinical Lecturer in Maternal & Fetal Medicine and Perinatal Epidemiology, KCL |
| Signature | [insert wet signature] |
| Date | 16/08/2018 |

Key Collaborators

| Name | Dr Asma Khalil |
| --- | --- |
| Role | Consultant in Maternal & Fetal Medicine, St George’s |
| Signature | [insert wet signature] |
| Date | 16/08/2018 |

| Name | Professor Jane Sandall |
| --- | --- |
| Role | Professor of Social Science and Women’s Health, KCL |
| Signature | [insert wet signature] |
| Date | 16/08/2018 |

Authorisation: Sponsor Representative

| Name | Professor Reza Razavi |  |
| --- | --- | --- |
| Role | Vice President and Vice Principal (Research), King’s College London |  |
| Signature | [insert wet signature] |  |
| Date | 22/08/2018 |  |

Authorisation: Trial Statistician

| Name | Dr Andrew Copas |  |
| --- | --- | --- |
| Role | Medical Statistician |  |
| Signature | [insert wet signature] |  |
| Date | 16/08/2018 |  |

| Name | Mr Paul Seed |
| --- | --- |
| Role | Medical Statistician |
| Signature | [insert wet signature] |
| Date | 16/08/2018 |

**Table of Contents**

1 Administrative information 8

1.1 Compliance 8

1.2 Sponsor 8

1.3 Structured trial summary 9

1.4 Roles and responsibilities 12

1.4.1 Protocol contributors 12

1.4.2 Co-investigators 12

1.4.3 Role of trial sponsor and funders 13

1.4.4 Trial Team 13

1.4.5 Trial Management Group 13

1.4.6 Trial Steering Committee / Data Monitoring Committee 14

1.4.7 Other Trial Oversight Groups 14

2 Trial Diagram 15

2.1 Diagram of cluster (hospital) participation 15

2.2 Diagram of individual management within participating clusters (hospitals) 15

3 Abbreviations 16

4 Glossary 18

5 Introduction 20

5.1 Background and Rationale 20

5.1.1 Explanation for choice of comparators 22

5.2 Objectives 22

5.3 Trial Design 22

6 Methods 26

6.1 Site Selection 26

6.1.1 Study Setting 26

6.1.2 Site/Investigator Eligibility Criteria 26

6.1.2.1 Principal Investigator’s (PI) Qualifications and Agreements 26

6.1.2.2 Resourcing at site 26

6.2 Site approval and activation 26

6.3 Clusters (Participants) 27

6.3.1 Eligibility Criteria 27

6.3.1.1 Cluster selection 27

6.3.1.2 Cluster Inclusion Criteria 28

6.3.1.3 Cluster Exclusion Criteria 28

6.3.1.4 Eligibility Criteria for Individuals Performing the Interventions 28

6.3.1.5 Co-enrolment Guidance 28

6.4 Interventions 28

6.4.1 Description and components 28

6.4.1.1 Training & accreditation 29

6.4.1.2 Protocols and guidelines 29

6.4.1.3 Audit 30

6.4.1.3.1 SGA rates and detection rates 30

6.4.1.3.2 Missed cases of SGA 30

6.4.1.4 Support and communication 30

6.4.2 Arm A 32

6.4.2.1 Intervention 32

6.4.2.2 Implementation schedule 32

6.4.3 Arm B 32

6.4.3.1 Intervention 32

6.4.3.2 Implementation schedule 32

6.4.4 Compliance and Adherence 32

6.4.5 Concomitant Care 32

6.5 Outcomes 33

6.5.1 Primary Outcomes 33

6.5.2 Secondary Outcomes 33

6.6 Clusters Timeline 34

6.6.1 Early-Stopping of Follow-up 35

6.6.2 Participant Transfers 35

6.6.3 Low compliance of intervention strategy 35

6.6.4 Trial Closure 35

6.7 Sample Size 35

6.8 Recruitment and Retention 37

6.8.1 Recruitment 37

6.8.2 Retention 37

6.8.3 Support mechanisms for interviewed women 37

6.9 Assignment of Intervention 38

6.9.1 Allocation 38

6.9.1.1 Sequence generation 38

6.9.1.2 Allocation concealment mechanism 38

6.9.1.3 Allocation Implementation 38

6.9.2 Blinding 38

6.9.3 Emergency Unblinding 38

6.10 Data Collection, Management and Analysis 38

6.10.1 Data Collection Methods 38

6.10.2 Data Management 40

6.10.3 Non-Adherence and Non-Retention 41

6.10.4 Statistical Methods 41

6.10.4.1 Statistical Analysis Plan 41

6.10.4.2 Statistical Methods – Outcomes 41

6.10.4.3 Additional Analyses - Subgroup 41

6.10.4.4 Additional Analyses – Adjusted 41

6.10.5 Analysis Population and Missing Data 41

6.10.6 Economic evaluation 42

6.10.6.1 Health Economic Analysis Plan 42

6.10.6.2 Within-trial economic analysis 42

6.10.7 Evaluation of implementation 43

6.11 Data Monitoring 44

6.11.1 Data Monitoring Committee 44

6.11.2 Interim Analyses 44

6.11.3 Data Monitoring for Harm 44

6.11.3.1 Safety reporting 45

6.11.3.2 Investigator responsibilities relating to safety reporting 46

6.11.3.2.1 Seriousness assessment 46

6.11.3.2.2 Severity or grading of Adverse Events 46

6.11.3.2.3 Causality 46

6.11.3.2.4 Expectedness 47

6.11.3.3 Notifications 47

6.11.3.3.1 Notifications by the Investigator to the sponsor 47

6.11.3.3.2 Sponsor (KCL) and trial team responsibilities 48

6.11.4 Quality Assurance and Control 48

6.11.4.1 Risk Assessment 48

6.11.4.2 Central Monitoring at KCL 48

6.11.4.3 On-site Monitoring 48

6.11.4.3.1 Direct access to clusters records 49

6.11.4.4 Trial Oversight 49

6.11.4.4.1 Trial Management Team 49

6.11.4.4.2 Trial Management Group 49

6.11.4.4.3 Independent Trial Steering Committee/ Data Monitoring Committee 49

6.11.4.4.4 Trial Sponsor 49

7 Ethics and Dissemination 50

7.1 Research Ethics Approval 50

7.2 Competent Authority Approvals 50

7.3 Other Approvals 50

7.4 Protocol Amendments 50

7.5 Consent or Assent 51

7.5.1 Consent or Assent in Ancillary Studies 52

7.6 Confidentiality 52

7.7 Declaration of Interests 52

7.8 Indemnity 52

7.9 Finance 53

7.10 Access to Data 53

7.11 Ancillary and Post-trial Care 53

7.12 Publication Policy 53

7.12.1 Trial Results 53

7.12.2 Authorship 53

7.12.3 Reproducible Research 53

8 Ancillary Studies 54

9 Protocol Amendments 55

10 References 58

11 Appendices 61

11.1 Appendix 1. GAP programme description 61

11.2 Appendix 2. TIDieR checklist 65

11.3 Appendix 3. Minimum requirements for GAP compliance in the DESiGN trial. 68

11.4 Appendix 4. Letter of support from SCN 69

11.5 Appendix 5. Letter of support from Tommy’s Charity. 70

11.6 Appendix 6. Letter of support from PPI representative. 71

11.7 Appendix 7. Letter of support from RCOG clinical study group on stillbirth. 72

11.8 Appendix 8. Letter of support from SANDS charity. 73

11.9 Appendix 9. Letter of support from NHS England. 74

11.10 Appendix 10. Cluster consent form from local clinical leads. 76

# 1 Administrative information

This document was constructed using the Comprehensive Clinical Trials Unit (CCTU) at UCL Protocol template Version 4. It describes the DESiGN trial, which was initially sponsored by UCL and co-ordinated by CCTU. There was a transfer of sponsorship to King’s College London (KCL) with effect from 2^nd^ of August, 2017.

It provides information about procedures for entering participants into the trial, and provides sufficient detail to enable: an understanding of the background, rationale, objectives, trial population, intervention, methods, statistical analyses, ethical considerations, dissemination plans and administration of the trial; replication of key aspects of trial methods and conduct; and appraisal of the trial’s scientific and ethical rigour from the time of ethics approval through to dissemination of the results. The protocol should not be used as an aide-memoire or guide for the treatment of other patients. Every care has been taken in drafting this protocol, but corrections or amendments may be necessary. These will be circulated to registered investigators in the trial. Sites entering participants for the first time should confirm they have the correct version through a member of the trial team.

CCTU supports the commitment that its trials adhere to the SPIRIT guidelines. As such, the protocol template is based on an adaptation of the Medical Research Council CTU protocol template (2012) and the Standard Protocol Items: Recommendations for Interventional Trials (SPIRIT) 2012 Statement for protocols of clinical trials (1). The SPIRIT Statement Explanation and Elaboration document (2) can be referred to, or a member of CCTU Protocol Review Committee can be contacted for further detail about specific items.

## 1.1 Compliance

The trial will be conducted in compliance with the approved protocol, the Declaration of Helsinki (2008), the principles of Good Clinical Practice (GCP) as laid down by the Commission Directive 2005/28/EC with implementation in national legislation in the UK by Statutory Instrument 2004/1031 and subsequent amendments, the Human Tissue (Quality and Safety for Human Application) Regulations 2007, the UK Data Protection Act, and the National Health Service (NHS) Research Governance Framework for Health and Social Care (RGF).

Participating sites will inform the trial team / KCL as soon as they are aware of a possible serious breach of the protocol, so that KCL can fulfil its requirement to report the breach if necessary to the Research Ethics Committee.

## 1.2 Sponsor

Following the transfer of sponsorship from UCL to KCL on the 2^nd^ of August, 2017:

KCL is the trial sponsor and has delegated responsibility for the overall management of the DESiGN trial to Division of Women’s Health. Queries relating to KCL sponsorship of this trial should be addressed to the KCL Director of Research Management and Innovation or via the trial team.

## 1.3 Structured trial summary

| Primary Registry and Trial Identifying Number | ISRCTN67698474 |
| --- | --- |
| Date of Registration in Primary Registry | 02/11/2016 |
| Secondary Identifying Numbers | Not applicable |
| Source of Monetary or Material Support | Tommy’s Charity  SANDS  Guy’s and St Thomas’ Charity |
| Sponsor | King’s College London |
| Contact for Public Queries | Design.trial@kcl.ac.uk |
| Contact for Scientific Queries | Dr Dharmintra Pasupathy  Senior Lecturer / Consultant in Maternal & Fetal Medicine  and Perinatal Epidemiology  Division of Women's Health  Women's Health Academic Centre KHP  10th Floor North Wing  St. Thomas' Hospital  Westminster Bridge Road  London SE1 7EH  Tel no: 020 7188 7188 ext 56316  Email: Dharmintra.Pasupathy@kcl.ac.uk |
| Public Title | Improving detection of small infants during pregnancy. |
| Scientific Title | **The DESiGN Trial -** Detection of small for gestational age fetus (SGA) – a cluster randomised controlled trial to evaluate the effect of the Growth Assessment Protocol (GAP) programme |
| Countries of Recruitment | UK |
| Health Condition(s) or Problem(s) Studied | Small for gestational age (SGA) fetus and infants |
| Intervention(s) | GAP programme. This includes comprehensive staff training, evidence-based protocols, routine monitoring of SGA and detection rates, regular audits of missed cases to help identify training needs and system failures in fetal growth surveillance, and ongoing communication and support between the Perinatal Institute and Trusts. |
| Key Inclusion and Exclusion Criteria | Inclusion and exclusion criteria into the study listed below is based on characteristics of the maternity unit (cluster). There are no inclusion or exclusion criteria based on women within each maternity unit (cluster). Data from all pregnant women within each maternity unit (cluster) will be collected.  Inclusion criteria: Maternity units which are willing to implement the GAP programme.  Exclusion criteria: Maternity units which have already implemented GAP or will not be introducing GAP. |
| Study Type | Cluster randomised controlled trial |
| Date of First Randomisation | 3^rd^ of November 2016 |
| Target Sample Size | 12 Maternity Units (Clusters). Power calculation based on average of 5000 birth per year per maternity unit (cluster) with a SGA prevalence of 10%. |
| Primary Outcome | Ultrasound detection of infants that are SGA (birthweight <10^th^ centile) by both customised and population standards that were detected antenatally after 24 weeks*.  * The antenatal charts used for ultrasound detection (numerator) will depend on the allocation arm of the trial. The denominator for the estimation of detection in each arm of the trial will be the same population of SGA infants (SGA by both customised and population). |
| Key Secondary Outcomes | A. Ultrasound detection of SGA at birth by customised centiles defined as the proportion of SGA infants (birthweight <10^th^ customised centile) that were detected antenatally by ultrasound scan after 24 weeks. We will determine the additional diagnostic test performance (specificity, false positive and false negative).  B. Ultrasound detection of SGA at birth by population centiles (UK90 population centiles) defined as proportion of SGA infants (birthweight <10^th^ population centile) that were detected antenatally by ultrasound scan after 24 weeks. We will determine the additional diagnostic test performance (specificity, false positive and false negative).  C. Effect on short term clinical outcomes  1. Neonatal – *general parameters* - gestational age at birth, birthweight, head circumference;  *parameters related to immediate condition at birth* - 5-min Apgar score <7, delivery with metabolic acidosis (arterial cord pH<7.1), respiratory support in delivery room;  *parameters related to NICU admission* – length of stay, level of care, *major neonatal morbidity* – one or more of the following - neonatal brain injury, supplementary oxygen requirements > 28 days, necrotizing enterocolitis, Culture-positive sepsis, retinopathy requiring ophthalmic intervention;  *parameters related to transitional care* – length of stay, neonatal morbidity – one or more of the following - hypothermia, hypoglycaemia, nasogastric tube feeding;  *perinatal loss* – stillbirth (antepartum and intrapartum), neonatal death (early and late), death before discharge (after 28 days of birth). In all perinatal losses we will also record cause of death.  2. Maternal - induction of labour; mode of delivery including caesarean section rates; postpartum haemorrhage (>1000ml); severe perineal trauma (3^rd^ / 4^th^ degree tear), length of stay in hospital; breast feeding at discharge; pre-eclampsia; gestational diabetes mellitus.  D. Health economics - number of ultrasound scans after 24 weeks; antenatal clinic / antenatal day unit activity; rates of induction of labour; rates of caesarean sections; length of maternal and neonatal stay; admissions and average length of stay in NICU / SCBU  E. Process evaluation of implementation: proportion of staff trained, staff assessed and women assessed with GAP programme; adherence to SGA risk stratification and management protocols and missed case analysis. Evaluation of acceptability and feasibility of intervention to staff and women, contextual barriers and facilitators and organisational impact.  F. Other methods of assessments of antenatal detection of SGA:  1. Ultrasound detection of SGA using different threshold (e.g. 5^th^ centile).  2. Clinical detection of SGA at birth (by customised centiles): defined as the proportion of SGA infants (birthweight <10^th^ customised centile) that were clinically detected antenatally (by ultrasound scan after 24 weeks and clinically defined/managed as SGA) in each arm.  3. Growth trajectories (fetal biometry and EFW) and Doppler parameters in the detection of SGA.  4. GROW ultrasound charts (which is single component of GAP) against standard population charts on classification of fetal growth (small for gestational age, appropriate for gestational age, large for gestational age). |

##

## 1.4 Roles and responsibilities

These membership lists are correct at the time of writing; please see terms of reference documentation in the TMF for current lists.

### 1.4.1 Protocol contributors

| Name | Affiliation | Role |
| --- | --- | --- |
| Dr Dharmintra Pasupathy | Division of Women’s Health, KCL | Design of trial  Protocol development  Review of protocol |
| Dr Asma Khalil | Department of Obstetrics, St George’s Hospital | Design of trial  Protocol development  Review of protocol |
| Professor Jane Sandall | Division of Women’s Health, KCL | Design of process evaluation  Design of trial  Review of protocol |
| Dr Matias Costa Vieira | Division of Women’s Health, KCL | Design of trial  Protocol development  Review of protocol |
| Dr Andrew Copas | UCL CCTU | Statistical analysis plan & sample size calculation  Protocol development  Review of protocol |
| Mr Paul Seed | Division of Women’s Health, KCL | Sample size calculation |
| Mrs Caroline Doré | UCL CCTU | Senior statistical oversight  Advice on design of trial  Review of protocol |
| Ms Susan Tebbs | UCL CCTU | Advice on design of trial  Protocol development  Review of protocol |

### 1.4.2 Co-investigators

| Name | Affiliation | Role |
| --- | --- | --- |
| Professor Peter Brockelhurst | UCL | Review of protocol  Director of UCL CTU |
| Professor Mark Johnson | Imperial | Review of protocol |
| Professor Debbie Lawlor | University of Bristol | Epidemiological advice on study design |
| Professor Lesley McCowan | University of Auckland | Advice on design of trial & GAP |
| Professor Neil Marlow | UCL | Advice on neonatal outcome measures |
| Professor Donald Peebles | UCL | Link with Strategic Clinical Network for participation of sites |
| Professor Andrew Shennan | KCL | Review of protocol |
| Professor Basky Thilaganathan | St George’s | Topic expert on fetal growth |
| Dr Andrew Copas | UCL | Topic expert on study design for cluster trials and also statistical expertise |
| Dr Annette Briley | KCL | Study implemention |
| Dr Kirstie Coxon | KCL | Design of process evaluation |
| Dr Andy Healey | KCL | Health economics |
| Dr Christoph Lees | Imperial | Topic expert on fetal growth |
| Dr Louise Page | West Middlesex NHS trust | Review of protocol |
| Dr Matias Costa Vieira | KCL | Design of trial  Protocol development  Review of protocol |
| Mrs Alessandro Alagna | Tommy’s Charity | Representation of patient group |

### 1.4.3 Role of trial sponsor and funders

| Name | Affiliation | Role |
| --- | --- | --- |
| R&D at GSTT/KCL | KCL/GSTT | Sponsor |
| Tommy’s Charity |  | Funder – no involvement in development and reporting of the trial. |
| Stillbirth & neonatal death charity (SANDS) |  | Funder – no involvement in development and reporting of the trial. |
| Guy’s and St. Thomas’ Charity (GST) |  | Funder – no involvement in development and reporting of the trial. |

### 1.4.4 Trial Steering Committee / Data Monitoring Committee

| Name | Affiliation | Role and responsibilities |
| --- | --- | --- |
| Professor Anna David | University College London | Chair |
| Professor Elizabeth Allen | London School of Hygiene and Tropical Medicine | Independent Statistician |
| Dr David Howe | University of Southampton | Obstetrician |
| Mrs Sue Tebbs | University of Birmingham | Trial Management |
| Professor Jillian Francis |  | Qualitative Researcher |

### 1.4.5 Other Trial Oversight Groups

| Name | Affiliation | Role and responsibilities |
| --- | --- | --- |
| UCL CCTU Protocol Review Committee | UCL CCTU | Reviewed and supported the development of the protocol (until the change of sponsorship) |
| Stillbirth Clinical Study Group | RCOG | Review of the protocol |
| R&D at GSTT/KCL | GSTT/KCL | Review of the protocol and oversight of general governance and compliance following transfer of sponsorship |

# 2 Trial Diagram

## 2.1 Diagram of cluster (hospital) participation


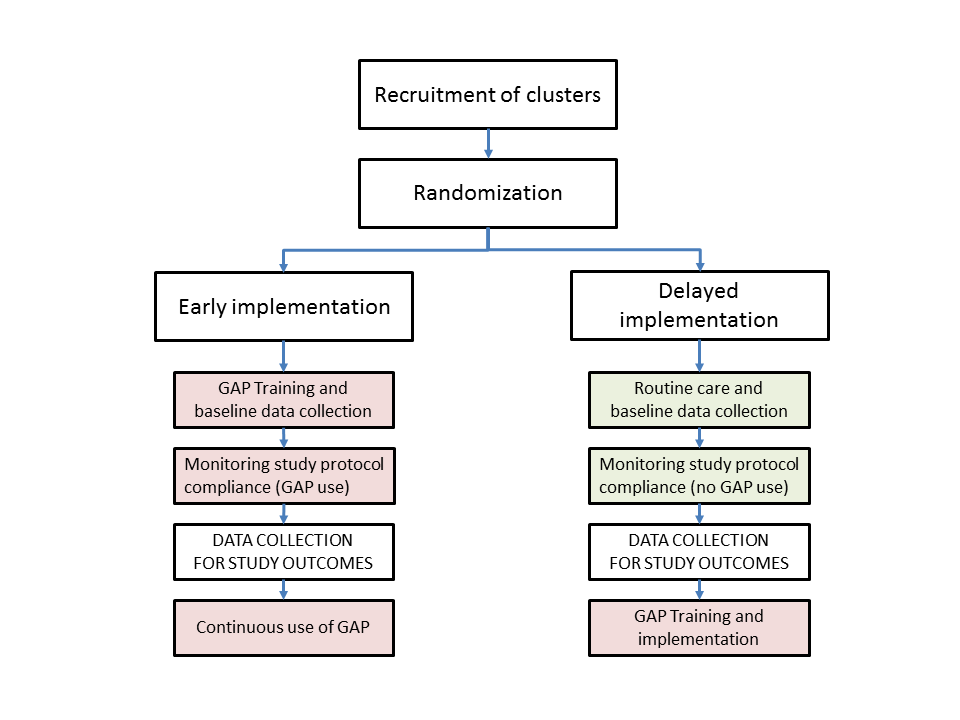


## 2.2 Diagram of individual management within participating clusters (hospitals)


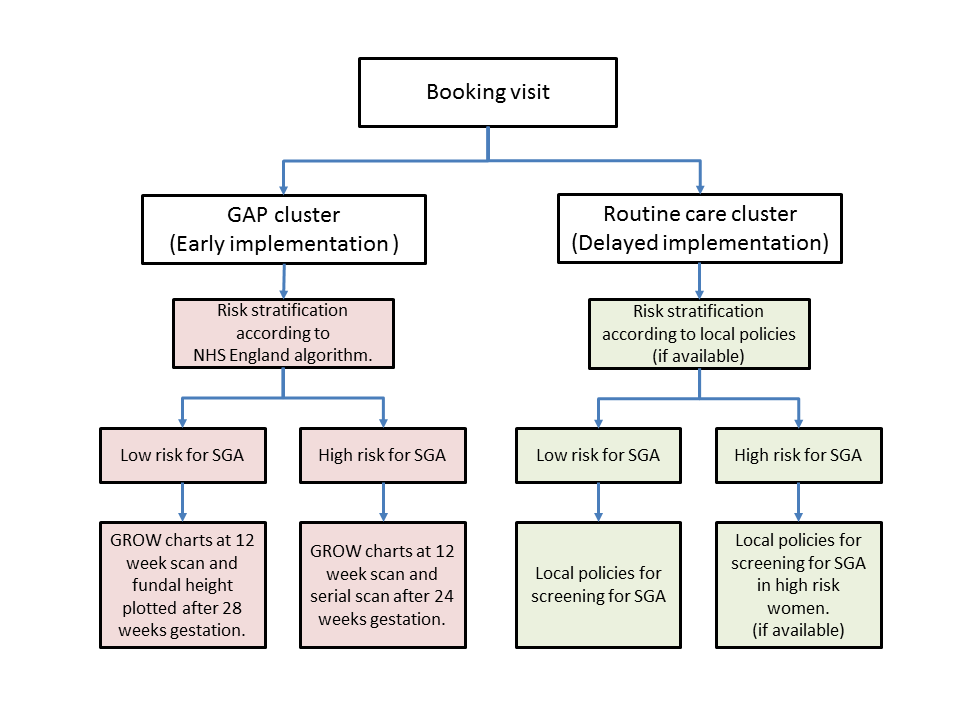


# 3 Abbreviations

| AE | Adverse Event |
| --- | --- |
| AR | Adverse Reaction |
| BMI | Body Mass Index |
| CI | Chief Investigator |
| CRF | Case Report Form |
| CTA | Clinical Trial Authorisation |
| CCTU | Comprehensive Clinical Trials Unit |
| DMC | Data Monitoring Committee |
| DSUR | Development Safety Update Report |
| EU | European Union |
| FDA | (US) Food and Drug Administration |
| FH | Fundal height |
| FWA | Federal Wide Assurance |
| GAP | Growth Assessment Protocol |
| GCP | Good Clinical Practice |
| GROW | Gestation Related Optimal Weight |
| ICH | International Conference on Harmonisation |
| IMP | Investigational Medicinal Product |
| IRB | Institutional Review Board |
| ITT | Intention to Treat |
| KCL | King’s College London |
| LGA | Large for Gestational Age |
| MHRA | Medicines and Healthcare products Regulatory Agency |
| NHS | National Health System |
| NICU | Neonatal Intensive Care Unit |
| NPEU | National Perinatal Epidemiology Unit |
| ONS | Office of National Statistics |
| PI | Principal Investigator |
| PIN | Participant Information Number |
| PIS | Participant Information Sheet |
| QA | Quality Assurance |
| QC | Quality Control |
| QMMP | Quality Management and Monitoring Plan |
| RCOG | Royal College of Obstetricians and Gynaecologists |
| RCT | Randomised Controlled Trial |
| R&D | Research and Development |
| REC | Research Ethics Committee |
| SAE | Serious Adverse Event |
| SAP | Statistical Analysis Plan |
| SAR | Serious Adverse Reaction |
| SFH | Symphysis Fundal height |
| SGA | Small for Gestational Age |
| SCBU | Special Care Baby Unit |
| SPC | Summary of Product Characteristics |
| SSA | Site Specific Approval |
| SUSAR | Suspected Unexpected Serious Adverse Reaction |
| TMF | Trial Master File |
| TMG | Trial Management Group |
| TMT | Trial Management Team |
| ToR | Terms of Reference |
| TSC | Trial Steering Committee |
| UCL | University College London |

# 4 Glossary

| ***Term*** *(and abbreviation if applicable)* | ***Definition*** |
| --- | --- |
| Breastfeeding at discharge | Feeding method stated on discharge from hospital. |
| Gestational age at delivery | Gestational age in weeks and days. Ideally calculated from 1^st^ trimester scan as per NICE guidelines (CG62 1.2.6). |
| Gestational diabetes mellitus | Ideally by an oral glucose tolerance test (as per NICE CG63/WHO) but otherwise by local hospital guidelines, as recorded in the hospital notes or IT records. |
| Large for gestational age by customised centiles | Birthweight above 90^th^ centile by customised centiles. |
| Large for gestational age by population centiles | Birthweight above 90^th^ centile by population centiles (UK90 population centiles). |
| Length of stay (maternal) | Length of time in days spent as an inpatient in hospital either from antenatal admission or from admission in labour or induction until discharge following birth. |
| Length of stay (neonatal) | Length of time in days spent as an inpatient in hospital following birth (includes time in NICU as well as postnatal or neonatal / paediatric ward). It does not count readmission. |
| Length of stay in Neonatal intensive care unit (NICU) admission. | Length of stay in days on NICU. |
| Length of stay in Special care baby unit (SCBU) admission. | Length of stay in days on SCBU |
| Major neonatal morbidity | One or more of the following - intraventricular haemorrhage, supplementary oxygen requirements> 28 days, necrotizing enterocolitis, sepsis, retinopathy of prematurity |
| Need for transitional care | Any duration of transitional care. |
| Neonatal intensive care unit admission. | Admission to NICU |
| Neonatal morbidity | One or more of the following - hypothermia, hypoglycaemia, nasogastric tube feeding |

| ***Term*** *(and abbreviation if applicable)* | ***Definition*** |
| --- | --- |
| Number of antenatal visits | Number of antenatal visits after 24 weeks per pregnancy. |
| Postpartum haemorrhage | Percentage of women giving birth who had an estimated blood loss of greater than 1000ml. |
| Pre-eclampsia | Clinical diagnosis of pre-eclampsia as recorded in the hospital notes or IT records. |
| Small for gestational age by customised centiles | Birthweight less that the 10^th^ centile by customised centiles. |
| Small for gestational age by population centiles | Birthweight less than the 10^th^ centile by population centiles (UK90 population centiles). |
| Severe perineal trauma | Defined as any third or fourth degree tear (affecting the anal sphincter muscle(s) or rectal mucosa) (RCOG GTG29). |
| Special care baby unit admission | Admission to SCBU |
| Stillbirth | Number of babies delivered without signs of life, ≥24+0 weeks of gestation. Expressed per 1000 births (live births and stillbirths). |

# 5 Introduction

## 5.1 Background and Rationale

The rate of stillbirth in the UK has changed little over the past 20 years and remains amongst the highest in developed countries (3, 4). The impact of a stillbirth is extensive, not only for the family but has consequences for the society. Reducing stillbirth is currently a national priority. Until recently two thirds of stillbirths were considered unexplained and, therefore unavoidable. Using a new classification system it has been demonstrated that 43% of babies who died in utero were small for gestational age (SGA) and 9% had placental insufficiency (5). A subsequent population-based study has shown that antenatal detection of SGA can halve the stillbirth risk (6){Gardosi, 2013 #519}. This suggests that improvements in the detection of SGA infants could have the potential to significantly reduce the incidence of stillbirths.

At present the antenatal detection of SGA is poor and antenatal identification is achieved in only about 1 in 4 cases (6-8). Improved antenatal detection of SGA is recognised to improve pregnancy outcome through appropriate antenatal surveillance and timely delivery (6, 9). SGA was traditionally defined as birthweight below the 10^th^ centile for gestational age and sex according to population references (4, 10). SGA by population centiles is associated with many adverse neonatal outcomes including stillbirth and perinatal death (11, 12). However, SGA by population birth weight centiles does not account for physiological maternal characteristics and includes the effects of pathological pregnancies, such as pre-eclampsia, that may affect fetal growth. Thus some infants, currently defined as SGA, may be appropriately grown for maternal constitution, whereas others which are growth restricted will not be defined as SGA. The concept of customised centiles attempts to address these issues and is based on three principles: individualised (adjustment for physiological factors that affect birth weight), optimised growth potential (excluding pathological factors affecting the weight standard such as smoking and diabetes) and use of fetal standards. The use of customised centiles, which adjust for maternal height, weight, ethnicity, parity, gestation at delivery, and fetal sex, identifies additional SGA fetuses which would not have been identified by conventional definitions (13). These infants who are SGA only by customised standards are at increased risk of adverse outcomes, including stillbirth. Crucially, fetuses that are considered SGA only by population centiles seem to have similar outcomes as appropriately grown fetuses (13).

Despite the evidence described above, customised centiles have been criticised because some factors might not have a physiological effect (14-16). This is especially true for maternal weight and ethnicity. Obesity is characterised by a metabolic disturbance that affects fetal growth and is associated with increased perinatal morbidity and stillbirth (17). However, Gardosi *et al.* have demonstrated that variation of maternal weight was not associated with increased risk of perinatal mortality, in women with a body mass index (BMI) within the normal range (20-25kg/m^2^). In the cohort with BMI >25kg/m^2^, there was no correlation between SGA rates by population centiles and rates of perinatal mortality. However rates of SGA by customised centiles and perinatal mortality were correlated (18). This study does not address the cause of perinatal mortality, which may not relate to SGA. The cause specific mortality related to SGA may differ by BMI category. The influence of ethnicity is more complex as it is recognised that there is an association between ethnicity and socio-economic deprivation (19, 20). Socioeconomic deprivation also has an association with antepartum stillbirth risk (21, 22). Therefore any adjustment for maternal ethnicity has the potential to adjust for the pathological effects of socioeconomic deprivation. In a recent publication from a multicentre international study, an international fetal standard was developed using 8 cohorts of pregnant women in optimal conditions, who were at low risk of fetal growth anomalies (23). The authors describe a similar ultrasound growth potential and birthweight distribution across all participating countries, which suggests that no physiological association exists between ethnicity and fetal weight, following exclusion of other potential confounding variables. Although, these Intergrowth data demonstrate that infants born in India to well-nourished women had a mean birthweight of 600g lower than infants born in the UK, using skeletal size as markers of growth there was considered to be sufficient similarity in the distribution of the data to construct an international pooled growth standard used for screening of growth anomalies (23, 24). Kierans *et al.* have used the Canadian stillbirth registry and reported that using population standards there was a higher rate of SGA in women of Chinese and South Asian ethnicity. However the perinatal mortality rate in this group was the lowest compared to other groups. Using customised standards, the rate of SGA was lower in Chinese and South Asian ethinicity and more importantly concordant with the prevalence of perinatal mortality in this population (25). According to current evidence, adjustment for ethnicity seems reasonable as it improves the detection of SGA infants at risk of morbidity and mortality although a pathological effect may exist and this may differ by socioeconomic groups between different populations studied.

At present in most UK obstetric units, the suspicion of fetal growth restriction is firstly assessed by palpation of the maternal abdomen. Symphysis fundal height (SFH) measurement is a screening tool for SGA, however, the accuracy is limited (26, 27). A non-randomised controlled trial of standardised fundal height (FH) measurement and estimated fetal weight plotted on customised charts demonstrated an increase in antenatal detection of small babies (48% vs. 29%, odds ratio 2.2; 95%CI 1.1-4.5) (28). Implementation of these charts was also tested in Australia where there was also a doubling in the detection rate of SGA compared with historical controls (29).

The Growth Assessment Protocol (GAP) is a training programme developed by the Perinatal Institute that consists of use of Gestation Related Optimal Weight (GROW) charts linked to risk assessment, management protocols and audit tools. GROW utilises a systematic method of measurement, achieved through a standardised training and accreditation programme, with the use of FH charts, developed in accordance with the principles of customisation. Estimated fetal weights from ultrasound assessment are also plotted on these customised fetal growth charts. The second component of GAP is the risk assessment and management protocols linked to key points from the RCOG Green-top Guideline on Investigation and Management of the Small-for-Gestational-Age fetus (30). It also includes a missed case audit tool to assess reasons for failure of antenatal recognition of SGA. The development of GAP as a comprehensive programme has been more recent with previous implementations being mainly focused on GROW with aspects of case reviews, audit and management guidance.

The use of the GAP / GROW programme has expanded since its development and is now implemented in 105 (64%) of UK Trusts (31). Gardosi *et al.,* have recently reported the impact of the programme in UK comparing regions with high uptake to regions with low uptake between 2007 to 2012 using data from Office for National Statistics (ONS) records (32). The results have demonstrated that high uptake of the programme was associated with a reduction in stillbirth rates. Overall, there was an impressive 22% reduction in stillbirth rates in the high uptake regions during the period analysed, which reflects the period before and after implementation of GAP / GROW. This observational study fulfilled the Bradford Hill (33) criteria for causality. However, it is recognised that the highest level of evidence is obtained from randomised controlled trials, which is lacking in this area as highlighted in a Cochrane review (34). Therefore a randomised controlled trial to accurately assess the GAP programme is imperative and timely.

Furthermore, the effect of the GAP on the management of pregnancies and other maternal and neonatal outcomes, such as caesarean section rates, induction of labour, gestational age at delivery, neonatal intensive care unit admission, prenatal detection of large for gestational age (LGA) infants, neonatal morbidity and length of stay in hospital is less well reported. Introduction of the GAP programme will also have an impact on health economics and clinical service provision, partly from the outcomes discussed above (such as induction of labour, caesarean section, and length of stay) but also related to utilisation of scanning, which requires evaluation. This independent evaluation will inform the planning of clinical service provision and inform national policy makers on financial implications for maternity care. This must also be balanced against the importance of some key neonatal outcomes (stillbirth, early neonatal death, neonatal morbidity due to brain injuries).

London has a prevalence of stillbirth above the national mean (2013 data: London 5.3/1000 (35) UK 4.8/1000 (36)) and the use of GAP in clinical practice is very low at present (approx. 5%). The London Maternity Network has recommended the use of GAP as a strategy for reduction in stillbirth rates and Trusts are increasingly interested in adopting this package. Given the incomplete usage in London and the current evidence base from observational studies this provides a unique opportunity to undertake a Cluster Randomised Controlled Trial (RCT) to assess the impact of the GAP programme. A study powered to investigate stillbirth as a primary outcome will require a large sample size (37, 38). To study a similar outcome and achieve similar power we will require 346 hospitals (clusters) per arm. It is recognised that improved detection of SGA is associated with a reduction in the risk of stillbirth (9). Therefore we propose a cluster RCT to evaluate the GAP programme as a strategy for improving the antenatal detection of SGA, including implementation evaluation and health economic assessment.

### 5.1.1 Explanation for choice of comparators

A cluster randomised controlled trial comparing the effect of the introduction of GAP to current clinical practice on pregnancy outcomes and service provision.

## 5.2 Objectives

- To determine whether implementation of the GAP programme will result in an improved ultrasound and clinical detection of SGA.
- To investigate the effect of the intervention on short-term maternal and neonatal outcomes
- To estimate the impact of GAP on clinical service provision and health economics.
- To assess fidelity and quality of implementation, acceptability and identify contextual factors associated with variation in outcomes of GAP in order to avoid type III error.

## 5.3 Trial Design

A cluster randomised trial to investigate whether the GAP package leads to improved ultrasound detection of SGA fetuses. However, there is no consensus on the appropriate standard to define SGA. At present, two definitions (population centiles or customised centiles) are being used in clinical practice in hospital throughout the UK. The GROW customised charts, which is one of the components of GAP programme, has the potential to substantially improve the antenatal detection of babies who are SGA at birth by customized standards. Whilst some of the babies who are SGA by population centiles but not by customised centiles may be detected antenatally by GAP, it is possible that the antenatal detection may be inferior compared to current practice in this group. Furthermore the GAP programme was not developed to identify babies who were SGA by population centiles only. With regards to the use of population chart (routine clinical practice), it is likely that it better detects infants that are SGA by population centiles whilst missing some infants that are SGA by customised centiles. In order to have a common group of infants at risk that should be detected by both interventions in the trial (GAP and routine clinical practice) we propose the use of SGA infant by both customised and population centiles. There is consensus that this group of infants have increased risk of adverse outcomes and evidence suggests these infants are at the highest risk of morbidity/mortality - detection of these babies are crucial. Our trial specifically aims to demonstrate the GAP programme leads to improved detection of SGA at birth by both customised and population centile (primary outcome). To further enhance the interpretation of the primary outcome we will also assess the ultrasound detection of SGA by customised centiles and the ultrasound detection of SGA by population centiles as secondary outcomes. We will also: (i) compare the effect of the intervention on secondary maternal, fetal and neonatal outcomes; (ii) evaluate the implementation of GAP and related economic outcomes; and (iii) explore other ultrasound parameter in the assessment of abnormal fetal growth.

The hospitals participating in the study are not currently using the GAP programme and there is a drive to implement GAP in an effort to improve the detection of SGA fetuses with the aim to reduce the incidence of stillbirths. Considering the nature of this intervention, a traditional individual randomized controlled trial is not feasible. Once a unit is trained in GAP it is not possible to randomize their participants to intervention or control due to contamination. A cluster trial is appropriate for the nature of this intervention. Each maternity trust is a cluster in this trial. The clusters will be randomly allocated to either an immediate or delayed implementation of GAP. In the immediate arm training and use of GAP will be instituted (Table 1) at the start of the trial. There will be an interval before the measurement of the study outcomes. This will ensure that in the early implementation arm all deliveries in which the outcomes are measured will have been assessed by the GAP programme during the entire pregnancy. This will allow comparisons of outcomes between the two arms of the trial.

| Table 1. Trial timetable (Gantt Chart). | | | | | | | | | | | | | | | | | | | | | |  |  |  |  |  |  |  |  |  |  |  |
| --- | --- | --- | --- | --- | --- | --- | --- | --- | --- | --- | --- | --- | --- | --- | --- | --- | --- | --- | --- | --- | --- | --- | --- | --- | --- | --- | --- | --- | --- | --- | --- | --- |
|  | **2016** | | | | **2017** | | | | | | | | | | | | | **2018** | | | | | | | | | | | | **2019** | | |
|  | **Pre-trial** | | | **Nov-Jan** | | | **Feb-Aug** | | | | | | | **Sept-Jan** | | | | | **Feb - Aug** | | | | | | | **Sept-Feb*** | | | | | | **Mar-Nov** |
|  |  | | | **1** | **2** | **3** | **4** | **5** | **6** | **7** | **8** | **9** | **10** | **11** | **12** | **13** | **14** | **15** | **16** | **17** | **18** | **19** | **20** | **21** | **22** | **23** | **24** | **25** | **26** | **27** | **28** | **29-36** |
| Protocol development | X | X |  |  |  |  |  |  |  |  |  |  |  |  |  |  |  |  |  |  |  |  |  |  |  |  |  |  |  |  |  |  |
| Funding application | X | X | X |  |  |  |  |  |  |  |  |  |  |  |  |  |  |  |  |  |  |  |  |  |  |  |  |  |  |  |  |  |
| Ethics and R&D approval |  | X | X |  |  |  |  |  |  |  |  |  |  |  |  |  |  |  |  |  |  |  |  |  |  |  |  |  |  |  |  |  |
| Funding activation** |  |  |  |  | X |  |  |  |  |  |  |  |  |  |  |  |  |  |  |  |  |  |  |  |  |  |  |  |  |  |  |  |
| Randomisation |  |  |  | X |  |  |  |  |  |  |  |  |  |  |  |  |  |  |  |  |  |  |  |  |  |  |  |  |  |  |  |  |
| Preparation of GAP |  |  |  | X | X | X |  |  |  |  |  |  |  |  |  |  |  |  |  |  |  |  |  |  |  |  |  |  |  |  |  |  |
| **Early implementation** |  |  |  |  |  |  |  |  |  |  |  |  |  |  |  |  |  |  |  |  |  |  |  |  |  |  |  |  |  |  |  |  |
| GAP |  |  |  |  |  |  |  |  |  |  |  |  |  |  |  |  |  |  |  |  |  |  |  |  |  |  |  |  |  |  |  |  |
| Training |  |  |  |  |  |  | X | X | X | X | X | X | X | X | X | X | X | X | X | X |  |  |  |  |  |  |  |  |  |  |  |  |
| Charts used at 12 weeks |  |  |  |  |  |  |  |  |  |  |  |  |  | X | X | X | X | X | X | X | X | X | X | X | X | X | X |  | X | X | X | X |
| Charts used at delivery |  |  |  |  |  |  |  |  |  |  |  |  |  |  |  |  |  |  | X | X | X | X | X | X | X | X | X |  | X | X | X | X |
| **Delayed implementation** |  |  |  |  |  |  |  |  |  |  |  |  |  |  |  |  |  |  |  |  |  |  |  |  |  |  |  |  |  |  |  |  |
| GAP Training |  |  |  |  |  |  |  |  |  |  |  |  |  |  |  |  |  |  |  |  |  |  |  |  |  |  |  |  |  |  |  | X |
| Charts used at 12 weeks |  |  |  |  |  |  |  |  |  |  |  |  |  |  |  |  |  |  |  |  |  |  |  |  |  |  |  |  |  |  |  | X |
| **Data collection / analysis** |  |  |  |  |  |  |  |  |  |  |  |  |  |  |  |  |  |  |  |  |  |  |  |  |  |  |  |  |  |  |  |  |
| Data collection *** | - | - | - | X | X | X | X | X | X | X | X | X | X | X | X | X | X | X | X | X | X | X | X | X | X | X | X | X | X | X | X |  |
| Comparison period |  |  |  |  |  |  |  |  |  |  |  |  |  |  |  |  |  |  |  |  |  |  |  |  |  | X | X | X | X | X | X |  |
| Monitoring study protocol compliance |  |  |  |  |  |  | X | X | X | X | X | X | X | X | X | X | X | X | X | X | X | X | X | X | X | X | X | X | X | X | X |  |
| Implementation fidelity and acceptability |  |  |  |  |  |  |  |  |  |  |  |  |  |  |  |  |  |  | X | X | X | X | X | X | X | X | X | X | X |  |  |  |
| Data monitoring |  |  |  |  |  |  |  |  |  |  |  |  |  |  | X | X | X | X | X | X | X | X | X | X | X | X | X | X | X | X | X | X |
| Statistical analysis |  |  |  |  |  |  |  |  |  |  |  |  |  |  |  |  |  |  |  |  |  |  |  |  |  |  |  |  |  |  |  | X |
| Report for funders and manuscript |  |  |  |  |  |  |  |  |  |  |  |  |  |  |  |  |  |  |  |  |  |  |  |  |  |  |  |  |  |  |  | X |
| Disseminating the results |  |  |  |  |  |  |  |  |  |  |  |  |  |  |  |  |  |  |  |  |  |  |  |  |  |  |  |  |  |  |  | X |

*Data comparison period – all trusts will be delivering babies with full exposure to GAP by this stage. If a trust is behind the schedule for participation in the study or implementation of GAP it will start the data collection on month 24 (this will allow for 4 month of data collection as a minimal requirement). ** Funding activation will commence in December 2016 for a period of 36 months. *** Retrospective data collection of women delivering in the pre-trial period will be performed to allow for assessment of completeness of electronic records and baseline data.

# 6 Methods

## 6.1 Site Selection

The trial sponsor has overall responsibility for site and investigator selection and has delegated this role to the trial team at the Division of Women’s Health.

### 6.1.1 Study Setting

Multicentre study involving antenatal care in the community and hospital maternity units in England.

### 6.1.2 Site/Investigator Eligibility Criteria

Once a site has been assessed as being suitable to participate in the trial, the trial team will provide them with a copy of this protocol.

To participate in the DESiGN trial, investigators and trial sites must fulfil a set of criteria that have been agreed by the DESiGN Trial Management Group (TMG) and that are defined below.

Eligibility criteria:

- A named clinician is willing and appropriate to take Principal Site Investigator responsibility
- Participating site has not implemented the GAP programme

Trial sites meeting eligibility criteria and that are accepted by the TMG as being suitable to recruit to the trial, will create a local DESiGN Trial Master File (TMF) to use when applying for Site-Specific Approval (SSA) or local institutional approval and for record keeping al trial documents, as applicable.

#### 6.1.2.1 Principal Investigator’s (PI) Qualifications and Agreements

Investigator(s) must be willing to comply with the principles of GCP, to permit monitoring and audit as necessary at the site, and to maintain trial documents updated.

#### 6.1.2.2 Resourcing at site

The investigator(s) should be able to demonstrate a potential for implementing the GAP package in their individual unit which will include cascading of training to all clinical and midwifery staff within the study time line. They should also have an adequate number of qualified staff and facilities available for the foreseen duration of the trial to enable them to conduct the trial properly and safely.

Sites will be expected to provide details for a lead for implementation and three representatives if they are randomized for early implementation.

The site should have sufficient data management resources to allow prompt data return to the trial team.

## 6.2 Site approval and activation

The trial manager or delegate will notify the PI in writing of the plans for site initiation. Sites will not be permitted to implement GAP including training until a letter for activation has been issued.

The site must conduct the trial in compliance with the protocol as agreed by the Sponsor and, by the regulatory authority(ies) (as appropriate), and which was given favourable opinion by the Research Ethics Committee (REC) and/or Institutional Review Board (IRB). The PI or delegate must document and explain any deviation from the approved protocol, and communicate this to the trial team.

A list of activated sites may be obtained from the Trial Manager.

## 6.3 Clusters (Participants)

### 6.3.1 Eligibility Criteria

As per a cluster randomized trial, all participants eligible for the GAP programme will be eligible for the trial.

Inclusion and exclusion criteria into the study is based on characteristics of the maternity unit (cluster). Hospitals that have fully implemented or will not be introducing GAP will not be eligible for participation.

#### 6.3.1.1 Cluster selection

There will be **NO EXCEPTIONS** (waivers) to eligibility requirements at the time of randomisation. Questions about eligibility criteria should be addressed PRIOR to attempting to randomise the cluster.

Clusters will be considered eligible for enrolment in this trial if they fulfil all the inclusion criteria and none of the exclusion criteria as defined in the protocol.

The strategic clinical network of London and the investigators have written to individual trusts inviting participation in a randomised controlled trial. We have received responses from 12 Trusts that have agreed to participate (table 2). Some Trusts have not yet responded and will be accepted if keen to participate in the trial. There are also some trusts outside London who are keen to participate in the study.

| Table 2. Trusts that agreed to take part in the trial. | | | |
| --- | --- | --- | --- |
|  | Trusts | Birth rate  (2013-2014 data) | Agreed to trial |
| 1 | London North West Healthcare NHS Trust | 4863 | YES |
| 2 | Chelsea and Westminster Hospital NHS Foundation Trust | 5713 | YES |
| 3 | Guy’s and St Thomas’ Hospital NHS Foundation Trust | 6788 | YES |
| 4 | Hillingdon Hospitals NHS Foundation Trust | 4042 | YES |
| 5 | Homerton University Hospital NHS Foundation Trust | 5877 | YES |
| 6 | Kingston Hospital NHS Foundation Trust | 5763 | YES |
| 7 | St George’s University Hospitals NHS Foundation Trust | 4967 | YES |
| 8 | Imperial College Healthcare NHS Trust | 8633 | YES |
| 9 | University College London Hospitals NHS Foundation Trust | 6175 | YES |
| 10 | West Middlesex University Hospital NHS Trust | 4774 | YES |
| 11 | Royal Surrey County Hospital NHS Foundation Trust | 3396 | YES |
| 12 | Croydon Health Services NHS Trust | 3952 | YES |

#### 6.3.1.2 Cluster Inclusion Criteria

Hospitals that are willing to implement GAP and willing to participate in the trial will be included.

#### 6.3.1.3 Cluster Exclusion Criteria

Hospitals that have fully implemented GAP will be excluded from the trial.

#### 6.3.1.4 Eligibility Criteria for Individuals Performing the Interventions

All clinical, midwifery and sonographers providing care in pregnancy.

#### 6.3.1.5 Co-enrolment Guidance

There is no limitation for participants to engage in other individual trials. However, it is expected that hospitals are not involved in other cluster trials investigating similar primary end points (detection of SGA).

## 6.4 Interventions

### 6.4.1 Description and components

The intervention is the GAP programme (Appendix 1). The GAP programme is a complex intervention for improved detection of SGA infants through risk stratification, serial fundal height or scans during second and third trimester and use of customized charts for assessment of fetal growth. This programme includes comprehensive staff training and accreditation, evidence-based management protocols, routine monitoring of SGA and detection rates, regular audits of missed cases to help identify training needs and system failures in fetal growth surveillance, and ongoing communication and support between the Perinatal Institute and Trusts (39). This is a continuous intervention and all these components will be maintained after implementation (except for the initial training). To ensure the intervention was comprehensively described a TIDeR checklist is available in Appendix 2.

The intervention, the GAP programme will be introduced at the level of the hospital (cluster). Clusters randomised to introduce GAP programme will implement training and protocols consistent with principles of GAP (Appendix 1). Women in hospitals randomised to GAP will undergo the following:

- They will be risk assessed for SGA and managed as per GAP protocol. Low risk women will be seen routinely in antenatal clinic. At these visits standardised FH measurements will be performed from 28 weeks. In high risk women serial ultrasounds after 24 weeks will be recommended.
- Customised FH and ultrasound charts will be generated at the first trimester ultrasound visit. FH measurements will be plotted on the customised FH chart. In low risk women any deviation in growth on these charts will result in recommendation for ultrasound measurement. Estimated fetal weight (EFW) from ultrasound measurements will be plotted on customised EFW charts for both low or high risk women whenever an ultrasound is done.

In the delayed implementation arm women will receive routine care as per current hospital practice on screening for SGA.

All components of the intervention are delivered face-to-face to women during their antenatal visits. Number of antenatal appointments needed will vary according to obstetric risk for women in both cluster with or without GAP. Implementation of GAP will not generate additional clinical visits. Risk assessment based on GAP principles will influence schedule of care or antenatal surveillance. Low risk women will not be subject to additional visits or procedures. However, the risk factors or growth abnormalities detected using GAP may trigger the need for additional visits or procedures for clinical reasons.

#### 6.4.1.1 Training & accreditation

The aim is to extend training to all staff engaged in antenatal care. The training will be provided by local trainers who will have received training from the Perinatal Institute. The local trainers will be responsible for cascading the training to multidisciplinary staff in individual units. E-learning and testing packages will also be available to reinforce training and facilitate assessment. Competency documents will be available which will reflect knowledge on fetal growth surveillance and clinical application. There will also be online training and competency logs to internally monitor uptake in the Trusts. The responsibility of training will be reliant on local units. We will assess each unit for trail compliance as per (section 6.4.3)

#### 6.4.1.2 Protocols and guidelines

The GAP offers a protocol template, including evidence-based recommendations to standardise practice in the use of customised growth charts and referral criteria, which clinicians can adapt and integrate in their Trust based protocols. It includes an NHS England algorithm (Figure 1) which is a simplified version of that in the RCOG Green-top guideline for risk assessment and management planning for women in relation to fetal growth surveillance (RCOG 2013) (30).

#### 6.4.1.3 Audit

##### 6.4.1.3.1 SGA rates and detection rates

Routine quarterly reporting of SGA and antenatal detection rates is considered an essential component of the GAP programme to allow accredited Trusts to monitor their performance and benchmark against other units with similar demographics. The GROW software has been enhanced to assist Trusts in the collection of this information and to provide the customised centile at birth for postnatal management. This trial protocol will independently evaluate the incidence of detection of SGA in all births during the pre-specified data collection period in each of the participating units. This will not be reliant on the voluntary data submission by the provider of care after delivery.

##### 6.4.1.3.2 Missed cases of SGA

Case reviews have highlighted many learning points for training, protocols and systems failures (40). GAP includes an audit tool to assess local issues relating to fetal growth surveillance. This trial protocol will also independently evaluate the incidence of missed SGA in all deliveries in the pre-specified data collection period within each of the participating units. This will not be reliant on the voluntary data submission by the provider of care after delivery.

#### 6.4.1.4 Support and communication

Based on the programme developed by the Perinatal Institute, Trusts are asked to nominate link persons from each speciality — a midwifery manager (eg head of midwifery, clinical risk manager, matron), an ultrasonographer and an obstetric/fetal medicine lead. These clinicians provide local leadership assisting all aspects of the implementation of the GAP and strengthening the link between their Trust and the GAP team at Perinatal Institute, supporting implementation and feeding back on progress and action plans.

The SCN for London programme team will support regular meetings with the implementation leads to support implementation.

Figure 1. NHS England protocol for screening for SGA fetuses.


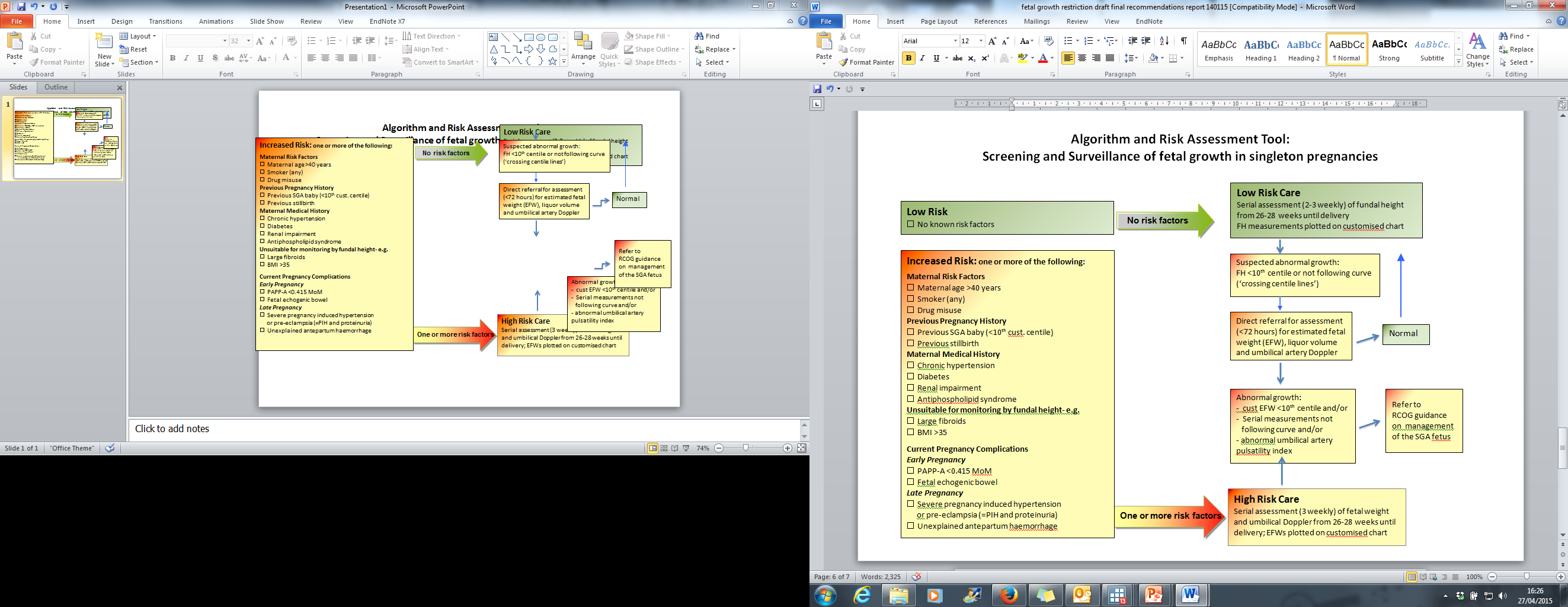


### 6.4.2 Arm A

#### 6.4.2.1 Intervention

GAP programme – this includes: risk stratification according to NHS England algorithm and generation of customised charts at 12 weeks and screening for SGA after 24-28 weeks with FH measurement or serial scans according to stratification of risk in early pregnancy. Customized charts are used as reference for plotting growth assessment (scans or FH measurement).

#### 6.4.2.2 Implementation schedule

Immediate implementation of GAP

### 6.4.3 Arm B

#### 6.4.3.1 Intervention

Current routine practice – this includes risk stratification according to local policies (if present) and screening for SGA after 24-28 weeks according to local policies (if present). Population charts are used as reference for plotting growth assessments (scans or FH measurement).

#### 6.4.3.2 Implementation schedule

Delayed implementation of GAP (following the data collection period)

### 6.4.4 Compliance and Adherence

Clusters (hospitals) will be randomised to immediate vs. delayed implementation of GAP. Individual clusters randomised to immediate implementation will receive and cascade the training and start using GAP (Table 1). The GROW web based software automatically calculates compliance of training and implementation of each cluster. The research team will also assess compliance based on number of staff receiving training and the generation of customised charts. The compliance will be checked during implementation so that we can assure that clusters are using GAP before starting the data collection. We will have pre-specified requirements to consider a cluster compliant which consist of assessment of proportion of deliveries using GAP, proportion of staff that completed training and confirm local guidelines and audit are in line with GAP recommendations (Appendix 3). Clusters without consistent adherence will be identified and members of Trial Team will meet with the Site PI to discuss strategies to improve compliance with GAP. Clusters not compliant during data collection will be analysed but the non-compliant status will be considered. Compliance will also be monitored during the data collection period.

### 6.4.5 Concomitant Care

We acknowledge that stillbirth is a national priority and programmes aimed at reducing stillbirth introduced during the period of study may minimise the effect of GAP. There are plans by NHS England to nationally role out a Care Bundle for the reduction of stillbirth. We have discussed the trial with the implementation team in NHS England and at present the date to introduce this care bundle has not been confirmed. The timescales for implementation nationally has also not been confirmed. The GAP programme is also within this care bundle and therefore evidence generated from this trial will provide information on the clinical and health service impact of a national programme. We have discussed with the Strategic Clinical Network in London and the implementation team of NHS England the need for the participating centres in the trial to be exempt from the national programme during the period of the trial.

## 6.5 Outcomes

### 6.5.1 Primary Outcomes

The primary outcome will be the ultrasound detection of infants that are SGA (birthweight <10^th^ centile) by both customised and population standards that were detected antenatally after 24 weeks.

* The antenatal charts used for ultrasound detection (numerator) will depend on the allocation arm of the trial. The denominator for the estimation of detection in each arm of the trial will be the same population of SGA infants (SGA by both customised and population).

| Table 3. Primary and secondary outcomes and their population | | | | | |
| --- | --- | --- | --- | --- | --- |
| Result of randomised detection method | **Study population** | | | |  |
|  | Not SGA | SGA by customised centiles only | SGA by population centiles only | SGA by both methods | Total |
| Detected | N1 | C1 | P1 | CP1 | T1 |
| Undetected | N0 | C0 | P0 | CP0 | T0 |
| Total | N | C | P | CP | T |
|  |  |  |  |  |  |
| Primary Outcome:  Detection of SGA by both population and customised centiles: CP1/CP  Secondary outcome:  Detection of SGA by customised centiles: (C1+CP1)/(C+CP) | | | | | |
| Detection of SGA by population centiles: (P1+CP1)/(P+CP) | | | | | |

### 6.5.2 Secondary Outcomes

A. Ultrasound detection of SGA at birth by customised centiles defined as the proportion of SGA infants (birthweight <10^th^ customised centile) that were detected antenatally by ultrasound scan after 24 weeks (us chart allocated by the study arm). We will determine the additional diagnostic test performance (specificity, false positive and false negative).

B. Ultrasound detection of SGA at birth by population centiles (UK90 population centiles) defined as proportion of SGA infants (birthweight <10^th^ population centile) that were detected antenatally by ultrasound scan after 24 weeks (us chart allocated by the study arm). We will determine the additional diagnostic test performance (specificity, false positive and false negative).

C. Effect on short term outcomes

1. Neonatal

1. General - gestational age at birth, birthweight, head circumference
2. Parameters related to immediate condition at birth - 5-min Apgar score <7, delivery with metabolic acidosis (arterial cord ph<7.1), respiratory support in delivery room;
3. Parameters related to NICU admission – length of stay, level of care, major neonatal morbidity – one or more of the following - neonatal brain injury, supplementary oxygen requirements at >28 days, necrotizing enterocolitis, culture-positive sepsis, retinopathy requiring ophthalmic intervention.
4. Parameters related to transitional care – length of stay, neonatal morbidity – one or more of the following - hypothermia, hypoglycaemia, nasogastric tube feeding
5. Perinatal loss – stillbirth (antepartum and intrapartum), neonatal death (early and late), death before discharge (after 28 days of birth). In all perinatal losses we will also record cause of death to determine the non-anomalous stillbirth.

2. Maternal

1. Induction of labour
2. Mode of delivery including caesarean section rates
3. Postpartum haemorrhage (>1000ml)
4. Severe perineal trauma -3^rd^ / 4^th^ degree tear
5. Length of stay in hospital
6. Breast feeding at discharge

D. Health economics

1. Number of ultrasound scans after 24 weeks
2. Antenatal clinic / antenatal day unit activity
3. Rates of induction of labour
4. Rates of caesarean sections
5. Length of maternal and neonatal stay
6. Admissions and average length of stay in NICU / SCBU

E. Process evaluation and intervention fidelity

1. Proportion of staff trained
2. Proportion of staff assessed
3. Proportion of women assessed with GAP/GROW programme
4. Missed case analysis
5. Organisational impact and unintended consequences
6. Acceptability and feasibility to women and staff, contextual barriers and facilitators, practice in control sites
7. Adherence to SGA risk stratification and management protocols

F. Other methods of assessments of antenatal detection of SGA:

1. Ultrasound detection of SGA using different threshold (e.g. 5^th^ centile).

2. Clinical detection of SGA at birth (by customised centiles): defined as the proportion of SGA infants (birthweight <10^th^ customised centile) that were clinically detected antenatally (by ultrasound scan after 24 weeks and clinically defined/managed as SGA) in each arm.

3. Growth trajectories (fetal biometry and EFW) and Doppler parameters in the detection of SGA.

4. GROW ultrasound charts (which is single component of GAP) against standard population charts on classification of fetal growth (small for gestational age, appropriate for gestational age, large for gestational age).

## 6.6 Clusters Timeline

The timeline for clusters was previously described in table 1 and in section 2.1. Hospitals (clusters) will be enrolled and allocated following local approval processes. Assessments will be similar in both groups including baseline data collection, data collection throughout implementation followed by assessment of study protocol compliance (GAP compliance for early intervention and routine care compliance in delayed intervention) and finally data collection on outcomes. The period of assessment of study compliance may be increased to ensure clusters in the early implementation fulfil minimum requirements of GAP programme before the data collection period. The intervention will be implemented in both arms of the trial but at different times. Data collection for outcome measures will be performed during the interval between implementation of GAP in both arms of the study.

### 6.6.1 Early-Stopping of Follow-up

Criteria will be developed with the TSC/DMC. If a cluster chooses to discontinue their trial intervention, they should continue to be followed up providing they are willing. They should be encouraged and facilitated not to leave the whole trial, even though they no longer adherent to the intervention schedule. If the cluster exercises the view that they no longer wish to be followed up either, this view must be respected and the cluster withdrawn entirely from the trial. KCL should be informed of the withdrawal in writing using the appropriate DESiGN trial documentation. Data already collected will be kept and included in analyses according to the data analysis plan. Clusters that stop the trial follow-up earlier than planned will not be replaced.

### 6.6.2 Participant Transfers

Not applicable

### 6.6.3 Low compliance of intervention strategy

In the immediate intervention arm, compliance to GAP will be monitored regularly and project midwives will be supported by meetings facilitated by the South London Group programme team. This will include adherence to training protocols, usage of customised charts and protocol development in line with recommendation from GAP (Appendix 3). In hospitals with low compliance the trial team will meet with the site investigator to develop strategies to improve compliance.

### 6.6.4 Trial Closure

The end of the trial will be 9 months after the last day of the data collection period. This is expected to be the end of month 36 of the trial (Table 1).

## 6.7 Sample Size

The power of the study is determined by the number of clusters (hospitals), mean size of clusters, intracluster correlation coefficient (or coefficient of variation between clusters), duration of data collection and prevalence of outcome.

Based on the annual number of deliveries in a sample of London Maternity Trusts, we assessed the mean births per year for 12 Trusts that are likely to participate in the trial (5053 births/year). During the data collection period (4 months) a mean of 42 SGA by customised centiles only; 42 SGA by population centiles only; and 126 SGA by both definitions are anticipated per cluster assuming that 12.5% of newborns are SGA by either definition (see distribution on Table 4). This represents a 60% overlap (126/210=60%) and in the unlikely event of a 33% overlap (assuming fixed a SGA rate of 10% by each definition), a mean of 84 SGA by customised and population centiles would be observed. At present in most units data on ultrasound detection of SGA is not routinely collected therefore we based our power calculation on data from the literature. The literature suggests that whether population or customised centiles are used to define SGA at birth, around 20% of SGA births are detected antenatally by ultrasound (7, 8, 41). We therefore assumed for our sample size the detection rates seen below in table 4 in the delayed arm. For the early implementation arm this represents an improvement in detection of SGA by both customised and population centiles from 20% to 33% (double the Odds Ratio) which we considered clinically meaningful (table 4).

For the ultrasound detection of SGA by population centiles, we assume detection of SGA (at birth by population centiles) in the delayed implementation group is 20% and in the early implementation group is 28% (Table 4). We select a non-inferiority margin of 5% corresponding to demonstrate the intervention leads to SGA detection of at least 15%.

Table 4: Expected number of SGA and performance of outcomes

|  | | **Number of SGA neonates / 10,000 births** according to pooled estimates of previous studies (16, 42-44) | | |
| --- | --- | --- | --- | --- |
|  |  | By population centiles only) | By both population & customised standards. **(PRIMARY OUTCOME)** | By customised centiles only |
| **Total observations*** | | 250 | 750 | 250 |
| Detection – delayed arm | % | 20% | 20% | 16% |
|  | N | 50 | 150 | 40 |
| Detection - Immediate implementation arm (GAP) | % | 12% | 33% | 33% |
|  | N | 30 | 250 | 83 |

We were unable to identify reports of intracluster correlation coefficient for clinical detection of SGA therefore coefficient of the most approximate outcome, fetal growth restriction, was used (0.019) (45). This leads to a design effect of 4.17 and effective sample size of 242 SGA newborns (by customised centiles, or equally for population centiles) in each study arm. The effective sample size for SGA by both customised and population centiles is 145 SGA newborns.

Based on these assumptions for the primary outcome and the expected 60% overlap between definitions, our study will provide 84% power to demonstrate superiority of GAP in detecting the SGA infants by both customised and population centiles. In a very extreme scenario of an overlap of 33%, our study will have 79% power to demonstrate GAP improves the detection of SGA infants by both customised and population centiles.

We also performed power calculation for two secondary outcomes. This sample size will also provide 91% power to demonstrate a superiority of GAP in detecting SGA by customised centiles and leads to 92% power to demonstrate non-inferiority of the intervention for the ultrasound detection of SGA by population centiles.

The precision of our power/sample size calculation is limited by the unknown values of the “true” intracluster correlation coefficient for detection of SGA. However, we were conservative in our other assumptions. Prevalence of SGA by customised centiles is around 13-15%, and we are assuming a prevalence of 10%. Also, previous papers have reported a greater difference in detection of SGA than doubling the OR (primary outcome). These conservative assumptions will allow some variation in the ICC.

## 6.8 Recruitment and Retention

### 6.8.1 Recruitment

We have identified 12 clusters (trusts) that are willing to participate in the trial. We are also seeking to increase the number of clusters to reduce the risk associated with loss of follow up.

### 6.8.2 Retention

The trial team will establish contact with a local site investigator for each cluster. Regular meetings throughout the period of study will be arranged to ensure compliance of the intervention and data collection throughout the study period. There will be regular email and newsletter updates to the participating clusters.

### 6.8.3 Support mechanisms for interviewed women

Women will be approached initially by their direct care midwives employed by Trusts, and if they agree, their contact details will be shared with study researchers with a view to inviting them to take part in an interview. Research midwives are trained to consider the context of recruitment carefully, and will liaise with senior staff at antenatal clinics to ensure that women are not approached at a time when they are distressed or experiencing negative events our outcomes during their pregnancy. SANDS has produced guidance, which advises that communication with women and families should be sensitive, clear and individualised, and we will follow these recommendations in our approach. In the event that women are willing to be interviewed after experiencing a poor outcome, we will ensure the approach encompasses the guidelines above, and that researchers are also adequately trained and supported to undertake interviews in these circumstances, and to signpost participants to support and other resources available to them.

It is reasonable to anticipate that for some respondents, talking through the events of pregnancy or birth may lead to recall of difficult or distressing events, and this is a risk of taking part in the interview. Although this risk is present, the research interviewers’ experiences of conducting in-depth interviews will provide them with skills to help manage this aspect of the research. In brief, if the respondent becomes upset during interview, the researcher will be careful to explore whether they agree to continue, and will suggest stopping or suspending the discussion if emotional discomfort is evident. If they prefer to discontinue, the researcher will remain with them until they are feeling better, and will also provide numbers of support services available to them.

Some respondents may feel comfortable during the interview, but could begin to dwell on their experiences afterwards. For this reason, we will offer contact details for (free of charge) support services to all participants, so that the provision of these is available to all respondents. If the researcher still feels any disquiet about any aspect of an interview, they will ask permission to make a courtesy call a week after the final interview, to check whether they (either mother or partner) wish to revisit any parts of the discussion, and the researcher would also discuss this with their supervisor through the research line management system.

## 6.9 Assignment of Intervention

### 6.9.1 Allocation

#### 6.9.1.1 Sequence generation

The method of allocation selected was the random permutation of clusters within each of two equally sized strata; clusters are divided in the two strata according to their size (deliveries per year in 2013-2014) and then randomized to either early or delayed implementation. Clusters that had the study approved in a later date were randomized in separate blocks. A minimum of two clusters was required to form a block for randomisation.

#### 6.9.1.2 Allocation concealment mechanism

Not applicable.

#### 6.9.1.3 Allocation Implementation

Allocation will be performed by the study statistician. The CI and the trial Team will coordinate with the Perinatal Institute and the individual cluster to facilitate GAP training and will monitor the implementation to ensure all clusters are working towards the timeline of the study.

### 6.9.2 Blinding

Not applicable

### 6.9.3 Emergency Unblinding

Not applicable.

## 6.10 Data Collection, Management and Analysis

### 6.10.1 Data Collection Methods

Source of data will vary according to the outcome assessed. Most of the data will be acquired from routine hospital data. This will include clinical notes and also electronic records (Table 5). Data obtained manually from clinical notes will be entered into a trial database by a member of the central research team. The hospital maternity electronic systems which record antenatal, ultrasound, intrapartum and postnatal data will be processed and uploaded into the local study file repository. Neonatal databases will also be accessed for outcome data. Data from these electronic records will be linked to a patient information number (PIN). The data will then be pseudo-anonymised at each local site (by a member of the DESiGN research team) and sent centrally through the NHS Digital Secure Electronic File Transfer system. At each local site there will be a record linking the PIN to the hospital ID. This will be stored locally. No patient identifiable data will be stored centrally. Data will be collected throughout the study (Table 1) and used for three purposes: (i) data from the period prior to implementation will be used as baseline data, (ii) data from training period full compliance and usage of GAP will be used for data monitoring and understand implementation of GAP, and (iii) data collection for a 6-month period (minimum 4 months required) will be used to assess primary and secondary outcomes of this study. To understand trends in clinical outcomes and service provision in all units we will also collect data from 12 months prior to the study.

| Table 5. Summary of data collection strategy. | |
| --- | --- |
| **Endpoint** | **Data collection** |
| Primary outcome | From electronic records - linked data from US Systems & maternity IT systems |
| Key secondary outcomes |  |
| A and B. Ultrasound test performance | From electronic records - linked data from US Systems & maternity IT systems |
| C.1. Neonatal morbidity | From electronic records - linked data from US Systems, maternity IT systems, neonatal IT systems and risk register |
| C.2. Maternal outcomes | From electronic records - data from Maternity IT systems and risk register |
| D. Health economics | From electronic records - data from Maternity IT systems, neonatal IT systems, appointments database |
| E. Process evaluation of implementation | Primary data collection, data from perinatal institute |
| F. Other methods of assessments of antenatal detection of SGA | From electronic records (linked data from US Systems & maternity IT systems) and primary data collection from review of notes (this is only be performed for the SGA infants at birth according to the maternity IT system). |

For measurement of primary outcome the SGA infants by both customised and population centiles will be identified using information from the maternity IT system, the bulk calculator of customised centiles provided by the Perinatal Institute and the calculator for population centiles (UK 90). Data will also be obtained from the maternity ultrasound IT systems to determine the ultrasound detection rate of SGA by both customised and population centiles. For the assessment of secondary outcomes related to ultrasound test performance the same approach will be used.

Data for secondary outcomes will be collected from each hospital electronic system. Data will be assessed for completeness. In routine maternity systems, there is mandatory recording on a number of key outcomes such as mode of delivery, onset of labour and breast feeding. For some outcomes such as admission to NICU we will validate our data by reviewing incident reports to local clinical risk committees. The validity of data collection for trial outcomes will be assessed during the baseline data collection period. Data on many of the neonatal outcomes will be identified from Badgernet which has widespread use in UK (above 90% of hospitals).

For health economic evaluation, data on service provision will be collected which will include length of stay, antenatal clinic appointments, antenatal day unit visits and number of ultrasound. These data will be collected from the hospital appointment systems and ultrasound software.

For process evaluation of implementation, quantitative data includes: proportion of staff trained, staff assessed; women assessed with GAP/GROW programme; adherence to SGA risk stratification and management protocols, missed case analysis data will be gathered from routine data collection by perinatal institute throughout the period on the intervention. Qualitative methods will be used to gather data which will include: semi-structured interviews with purposively sampled key stakeholders and health professionals (around 8 per early implementation site) and semi-structured interviews with a purposive sample of women (up to 5 per early implementation site). In control (delayed implementation) sites, interviews with a purposive sample of key stakeholders will elicit current practice (approximately 2/site). The researchers will where possible conduct face-to-face interviews. Stability or change in relation to the implementation may be explored through shorter contacts following the main interviews with key stakeholders only. Interviews will be arranged at times which are convenient with participants, and using settings and approaches which the participant prefers (including phone interviews where these are preferred by participants, or where face to face meetings prove difficult or are potentially onerous).

For recording other methods of detection of SGA, research midwives will review the maternity notes of SGA infants to identify the number of clinically detected SGA by study criteria. This approach will be used in both arms of the study and will provide the clinical detection rate of SGA. Additional maternity notes during the study period will also be accessed to review missed cases and aspects of evaluation of implementation. Information will be recorded in the trial database without any personal identification.

For assessment of GROW component, data will be obtained from link registries of maternity and ultrasound systems and will provide data regarding the ability of ultrasound to detect SGA fetuses. This antenatal detection will be performed for both population and customised centiles and will test their performance as a diagnostic test (sensitivity, specificity, false positive and false negative rates).

Information from the clusters will also be collected. This will include characteristics such as size of the cluster, ethnic predominance, description of socioeconomic level of population level of complexity of care, number of midwives, number of sonographers, number of consultants, presence of consultants in birth centre, rates of stillbirth and any specific pathway of antenatal care different from standard recommendation (ie. routine 3^rd^ trimester ultrasound).

### 6.10.2 Data Management

Each centre will upload the data files from the hospital’s IT systems into a secured access-restricted folder. Data collected from audit of patient notes by the research team will be entered in the approved DESiGN trial database by a member of the DESiGN trial team at local sites and protected using established procedures. The data will then be pseudo-anonymised by a member of the trial team and sent to GSTT using the NHS Digital Secure Electronic File Transfer system.

Coded data: The data in the GSTT file repository will be processed and stored on the servers based at KCL. The server will be password protected and only accessible to members of the DESiGN trial team at KCL, and external regulators if requested. The servers are protected by firewalls and are patched and maintained according to best practice. The physical location of the servers is protected by CCTV and security door access.

The file repository, server and coding frames will be developed by the trial team in conjunction with the Data Programmer.

After completion of the trial, the pseudo-anonymised data will be retained on the servers of KCL for on-going analysis of secondary outcomes.

The identification, screening and enrolment logs, linking participant identifiable data to the Participant Identification Number, will be held locally by the trial site. This will either be held in written form in a locked filing cabinet or electronically in password protected form on hospital computers. After completion of the trial the identification, screening and enrolment logs will be stored securely by the sites for 5 years unless otherwise advised by the sponsor.

### 6.10.3 Non-Adherence and Non-Retention

Data on reasons of non-compliance to implementation schedule and withdrawal from the trial will be recorded.

### 6.10.4 Statistical Methods

#### 6.10.4.1 Statistical Analysis Plan

A full SAP will be prepared at the start of the study. All analysis will acknowledge the clustering of individual participants by centre.

#### 6.10.4.2 Statistical Methods – Outcomes

The final decision as to method of analysis will be made once the final number of participating clusters is known. If no more than 12 clusters participate then due to the instability of other approaches, analysis will be by cluster-summary statistic approaches, i.e. calculating the proportion with the primary outcome for each cluster and comparing these values between intervention and control arms using a t-test. This approach includes particular approaches to calculation of effect size and 95% confidence intervals. Should however more clusters participate then more efficient approaches based on an analysis of individual participant data, such as mixed effects models with a random effect for each cluster, will be used which leads naturally to an estimate of effect size (odds ratio for primary outcome) and 95% confidence interval. Subject to the final design for the trial it will be decided whether relevant baseline data are available and if so than an ANCOVA type analysis will be conducted in which adjustment is made for the cluster summary value of the outcome from the baseline period.

#### 6.10.4.3 Additional Analyses - Subgroup

We will assess the effect of the intervention in each of the 4 following groups: SGA at birth by customised centiles only; SGA at birth by population centiles only; SGA at birth by both definitions; and not SGA by both standards. In addition a subgroup analysis of pregnancies at low risk and high risk for delivering a SGA baby will be performed. The definition of risk will follow the proposed in Figure 1.

#### 6.10.4.4 Additional Analyses – Adjusted

As mentioned above adjustment for baseline cluster summary values will be made if these data are collected. Adjustment will also be made for ethnicity. The adjusted analysis will be considered the main analysis.

### 6.10.5 Analysis Population and Missing Data

The primary outcome is determined only for pregnancies that were SGA by customised and by population centiles, so this forms the primary analysis population. In secondary analysis we will also consider the detection of SGA by ultrasound amongst all birth including those non SGA by either standard. Other secondary analyses such as number of ultrasound scans will also be conducted among all births or amongst specific subgroups as described earlier.

Multiple pregnancies and fetal congenital abnormalities detected before birth are going to be excluded from the primary outcome analysis because detection of SGA in these situations should not be related to GAP or standard care.

### 6.10.6 Economic evaluation

#### 6.10.6.1 Health Economic Analysis Plan

General methodological approach.

A cost-effectiveness analysis (CEA) will be carried out. This will include evaluating the cost impact of the GAP programme along-side evidence relating to the primary clinical outcome of interest (detection of SGA). This will enable an assessment of whether the GAP programme was either dominant in cost-effectiveness terms (e.g. whether it is broadly cost neutral or cost reducing but also more effective in detection of SGA) or whether there is evidence of a trade-off if the GAP programme is shown to be cost-increasing while at the same time improving rates of SGA detection. If the latter applies, the economic evaluation will estimate the incremental cost per additional SGA case detected. Health economic modelling will be used to estimate the incremental cost per additional stillbirth avoided. and the incremental cost per additional year of life gained through reduced incidence of still birth. Given that impact of GAP on incidence of stillbirth will not be directly evaluated within the trial, cost per stillbirth avoided will be indirectly inferred drawing on published evidence on the link between improved detection of SGA and reduced risk of stillbirth.

The economic evaluation will use hospital data from hospital records and also individual level data to quantify clinical activity volumes relating to pregnancy and neonatal care within the intervention and control sites included in the trial. This approach has been chosen as it will not be possible to identify some important items of resource use through clinical records at the patient-level alone. National and locally applicable unit costs will be used to convert clinical activity measures into costs and subsequently to evaluate the difference between pregnancy and neonatal care costs between the intervention and control sites.

The economic evaluation will be carried from the perspective of the NHS Trusts but will be limited to an assessment of impact on the cost of hospital-based clinical activity, thereby excluding community-based resource impacts including those arising through contact with primary care services.

#### 6.10.6.2 Within-trial economic analysis

The economic evaluation will draw on the wider design features of the trial to evaluate the impact of the GAP programme on the cost of pregnancy and neonatal care. We will use monthly data on clinical activity (translated into costs) over periods before and after the implementation of the GAP programme in each of the intervention sites. The availability of clinical activity data for control sites will facilitate a “difference-in-difference” design. This is a widely used quantitative approach used in economic and social scientific research to evaluate policy effectiveness.

This calculates the programme impact by comparing the average change in hospital costs over time (pre-and post-implementation) within the intervention and control sites using statistical modelling. The reliability of the difference in difference estimate as a measure of impact is dependent on key assumptions within the data being met, including, in this case, comparable pre-implementation trends in clinical activity between intervention and control sites. This will be assessed as part of the evaluation.

Evidence on cost impact from this analysis will be combined with evidence from the main trial on clinical impact to deliver an assessment of cost-effectiveness (see previous section). Sensitivity analysis will be undertaken to test the impact of uncertainty on key conclusions, particularly in relation to the estimation of pregnancy and neonatal costs of care. Also, a more detailed analysis will be carried out for specific resources more likely to be influenced by the intervention.

### 6.10.7 Evaluation of implementation

The process evaluation, aims to understand the functioning of the intervention by examining implementation, mechanisms of impact, and contextual factors. Implementation of the intervention will be evaluated via a mixed-methods approach drawing on the MRC framework for trials of complex interventions (46, 47). Based on Steckler and Linnan’s framework (48), key dimensions of implementation include: **Implementation process** – the structures, resources and mechanisms through which delivery is achieved; **Fidelity** – the consistency of what is implemented with the planned intervention; **Adaptations** – alterations made to an intervention in order to achieve better contextual fit; **Dose** – how much intervention is delivered; **Reach** – the extent to which a target audience comes into contact with the intervention. **Mechanisms of impact** – the intermediate mechanisms through which intervention activities produce intended (or unintended) effects. The study of mechanisms may include: **Participant responses** – how participants interact with a complex intervention.

We will describe the intervention and the mechanisms through which it is expected to produce change in a specific context using the TIDieR guidance and produce a logic model which informs data items for the evaluation of implementation (49). An assessment of each element of the GAP programme will be undertaken (that training and intervention are being delivered as planned and are acceptable according to providers daily work, impact on the clinical pathways that may ensue from the potential increased workloads in referrals, and that contextual influences are understood and addressed).

For process evaluation of implementation, descriptive quantitative information on fidelity, dose and reach will enable us to consider more detailed modelling of variations between participants or sites in terms of factors such as fidelity or reach (e.g. are there ethnic or socioeconomic biases in who is reached?). Quantitative data includes: proportion of staff trained; proportion of staff assessed; proportion of women assessed with GAP programme; adherence to SGA risk stratification and management protocols, missed case analysis data will be gathered from routine data collection by perinatal institute throughout the period on the intervention.

For evaluation of acceptability and feasibility of intervention to staff and women, contextual barriers and facilitators and organisational impact, gathered through qualitative data collected at six intervention sites which will include: semi-structured interviews with purposively sampled key stakeholders and health professionals (around 8 per early implementation site) and semi-structured interviews with a purposive sample of women (up to 5 per early implementation site). In control (delayed implementation) sites, interviews with a purposive sample of key stakeholders will elicit current practice (approximately 2/site).

The researchers will where possible conduct face-to-face interviews. Stability or change in relation to the implementation may be explored through shorter contacts following the main interviews with key stakeholders only. Interviews will be arranged at times which are convenient with participants, and using settings and approaches which the participant prefers (including phone interviews where these are preferred by participants, or where face to face meetings prove difficult or are potentially onerous).

### 6.10.8 Secondary Analysis

This study will offer the opportunity to explore ultrasound patterns of growth in different conditions and to assess the ultrasound detection of LGA. In addition, it will provide the opportunity to explore the epidemiology of other adverse pregnancy outcomes such preterm birth, pre-eclampsia, gestational diabetes, caesarean section, postpartum haemorrhage and neonatal morbidity. Other health economic analysis will be performed including the cost utility analysis to assess the cost per year of life gained (based on evidence of life expectancy at birth). We will also explore whether there are the necessary supporting data reported elsewhere to enable translation of life years gained through improved detection of SGA into quality adjusted life years (QALYs).

## 6.11 Data Monitoring

### 6.11.1 Data Monitoring Committee

The joint Trial Steering Committee (TSC)/Data Monitoring Committee (DMC) will meet regularly, as required, to assess any change in maternal, fetal and neonatal outcomes. They will have access to all data available from the trial, including adverse events reported.

Further details of the roles and responsibilities of the joint TSC/DMC, including membership, decision making processes, and the timing and frequency of interim analyses (and description of stopping rules and/or guidelines where applicable) are described in detail in the DESiGN trial TSC/DMC Terms of Reference (ToR).

### 6.11.2 Interim Analyses

The TSC/DMC will assess adverse outcomes and potential harms of both arms of the trial. The committee will monitor the rates of stillbirth per study arm and per cluster in four months period. An increase of 50% on stillbirth rates in a single cluster will prompt investigation of the site.

No formal stopping rules are planned. Following the Peto Principle (50), the TSC/DMC will recommend that the trial should continue unless the evidence in favour of one treatment is so overwhelming that it would be unethical to continue the trial. A P-value of <0.001 for the primary endpoint (51) may be used as guidance, but the TSC/DMC will be aware of the practical implications of a decision to stop; in particular whether it will make the GAP programme available more quickly or more generally.

These is the suggested approach but the TSC/DMC will decide in their initial meetings if it is appropriate and additional rules for monitoring will be developed. These committees are independent of the investigators and the sponsor and have the rights to decide when to stop or continue the trial.

### 6.11.3 Data Monitoring for Harm

The non-medicinal intervention being tested in this trial is not expected to have considerable side effects. The intervention is composed of measurement of FH and prompt referral for ultrasound where needed. Side effects of FH may consist of maternal discomfort due to semi-recumbent position and discomfort due to increased sensitivity of skin. Ultrasound is a mechanical wave and can theoretically increase the temperature in the studied tissue. The Doppler ultrasound uses higher energy and focuses in a smaller volume of tissue resulting in greater changes in temperature. In a clinical obstetric scenario, however, the increase in temperature is less than one degree Celsius, which is not considered clinically significant. The World Health Organisation performed a systematic review of 61 publications on the subject and reported no association with adverse maternal, fetal and neonatal outcomes (52). Both components of the intervention are used in different levels on routine care, therefore any of the above cannot be strictly assigned to the intervention.

Other adverse events however can happen due to incorrect use of the GAP tool. This means mistakes in the manual plotting in the FH on GROW chart can lead to an inappropriate management that can ultimately end in a serious adverse event. The audit of missing SGA cases and the review of all stillbirth cases will be performed locally in each cluster as recommended by GAP. Any event associated with misuse of GAP should be reported adverse event in the trial.

The local lead clinician (site investigator) will assess all participants with adverse events and report according to the description below.

#### 6.11.3.1 Safety reporting

Definitions of harm of the EU Directive 2001/20/EC Article 2 based on the principles of ICH GCP apply to this trial (Table 6).

Table 6: Adverse Event Definitions

| **Adverse Event (AE)** | Any untoward medical occurrence in a patient or clinical trial participant administered a medicinal product and which does not necessarily have a causal relationship with this product. |
| --- | --- |
| **Adverse Reaction (AR)** | Any untoward and unintended response to an investigational medicinal product related to any dose administered |
| **Unexpected Adverse Reaction (UAR)** | An adverse reaction, the nature or severity of which is not consistent with the applicable product information (eg Investigator’s Brochure for an unauthorised product or summary of product characteristics (SPC) for an authorised product. |
| **Serious Adverse Event (SAE) or Serious Adverse Reaction (SAR)** | Any AE or AR that at any dose:   - results in death - is life threatening - requires hospitalisation or prolongs existing hospitalisation - results in persistent or significant disability or incapacity - is a congenital anomaly or birth defect |

Adverse events include:

- Missed cases of SGA related to inappropriate plotting on charts or incorrect interpretation of GAP.
- SGA stillbirth related to inappropriate plotting on charts or incorrect interpretation of GAP.
- Maternal death

Adverse events do NOT include:

- Missed cases of SGA associated with lack of resources or delay in achieving the correct management.
- Non-SGA stillbirth.
- SGA stillbirth related to fetal abnormality.
- SGA stillbirth associated with lack of resources or delay in achieving the correct management.

#### 6.11.3.2 Investigator responsibilities relating to safety reporting

All non-serious AEs and ARs, whether expected or not, should be recorded in the patient’s medical notes and reported in the appropriate form and sent to the sponsor. SAEs and SARs should be notified to the sponsor immediately the investigator becomes aware of the event (in no circumstance should this notification take longer than 24 hours).

##### 6.11.3.2.1 Seriousness assessment

When an AE or AR occurs, the investigator responsible for the care of the participant must first assess whether or not the event is serious using the definition given in Table 6. If the event is classified as ‘serious’ then an SAE form must be completed and the sponsor (or delegated body) notified within one working day.

##### 6.11.3.2.2 Severity or grading of Adverse Events

The investigator should make an assessment of severity for each SAE and record this according to one of the following categories:

- **Mild**: an event that is easily tolerated by the participant, causing minimal discomfort and not interfering with every day activities.
- **Moderate**: an event that is sufficiently discomforting interfere with normal every day activities.
- **Severe**: an event that prevents normal every day activities.

Note: the term ‘severe’, used to describe the intensity, should not be confused with ‘serious’ which is a regulatory definition based on participant/event outcome or action criteria. For example, a headache may be severe but not serious, while a minor stroke is serious but not severe.

##### 6.11.3.2.3 Causality

The investigator must assess the causality of all serious events or reactions in relation to the trial therapy using the definitions in Table 7.

Table 7: Causality definitions

| Relationship | Description | Event type |
| --- | --- | --- |
| Unrelated | There is no evidence of any causal relationship | Unrelated SAE |
| Unlikely to be related | There is little evidence to suggest that there is a causal relationship (e.g. the event did not occur within a reasonable time after administration of the trial medication). There is another reasonable explanation for the event (e.g. the participant’s clinical condition or other concomitant treatment) | Unrelated SAE |
| Possibly related | There is some evidence to suggest a causal relationship (e.g. because the event occurs within a reasonable time after administration of the trial medication). However, the influence of other factors may have contributed to the event (e.g. the participant’s clinical condition or other concomitant treatment) | SAR |
| Probably related | There is evidence to suggest a causal relationship and the influence of other factors is unlikely | SAR |
| Definitely related | There is clear evidence to suggest a causal relationship and other possible contributing factors can be ruled out. | SAR |

##### 6.11.3.2.4 Expectedness

This relates to adverse reactions and serious adverse reactions and therefore is not required in this non cTIMP trial.

#### 6.11.3.3 Notifications

##### 6.11.3.3.1 Notifications by the Investigator to the sponsor

The sponsor must be notified of all SAEs within 1 working day of the investigator becoming aware of the event.

Investigators should notify the sponsor of any SAEs and other Notifiable Adverse Events (NAEs) occurring from the time of randomisation until 30 days after the last protocol treatment administration. SARs and SUSARs must be notified to the sponsor until trial closure.

The SAE form must be completed by the investigator (the consultant named on the delegation of responsibilities list who is responsible for the participant’s care) with attention paid to the grading, causality and expectedness of the event. In the absence of the responsible investigator, the SAE form should be completed and signed by a member of the site trial team and emailed as appropriate within the timeline. The responsible investigator should check the SAE form at the earliest opportunity, make any changes necessary, sign and then email to the sponsor. Detailed written reports should be completed as appropriate. Systems will be in place at the site to enable the investigator to check the form for clinical accuracy as soon as possible.

The minimum criteria required for reporting an SAE are the trial number and date of birth, name of reporting investigator and sufficient information on the event to confirm seriousness. Any further information regarding the event that is unavailable at the time of the first report should be sent as soon as it becomes available.

The SAE form must be scanned and sent by email to the trial team at **design.trial@kcl.ac.uk**

Participants must be followed up until clinical recovery is complete and laboratory results have returned to normal or baseline values, or until the event has stabilised. Follow-up should continue after completion of protocol treatment and/or trial follow-up if necessary. Follow-up SAE forms (clearly marked as follow-up) should be completed and emailed to the sponsor as further information becomes available. Additional information and/or copies of test results etc may be provided separately. The participant must be identified by trial number, date of birth and initials only. The participant’s name should not be used on any correspondence and should be blacked out and replaced with trial identifiers on any test results.

##### 6.11.3.3.2 Sponsor (KCL) and trial team responsibilities

Medically qualified staff at KCL and/or the Chief Investigator (CI or a medically qualified delegate) will review all SAE reports received. In the event of disagreement between the causality assessment given by the local investigator and the CI, both opinions and any justifications will be provided in subsequent reports.

The delegated staff at KCL will review the assessment of expectedness and, based on possible wider knowledge of the reference material for the treatment or comparator, and after discussion with the CI, may over-rule the investigator assessment of expectedness for the purposes of onward reporting.

KCL is undertaking the duties of trial sponsor and is responsible for the reporting of SUSARs and other SARs to the regulatory authorities (MHRA and competent authorities of other European member states and any other countries in which the trial is taking place) and the RECs as appropriate. Fatal and life threatening SUSARs must be reported to the competent authorities within seven days of KCL becoming aware of the event; other SUSARs must be reported within 15 days.

KCL will keep investigators informed of any safety issues that arise during the course of the trial.

The trial manager or delegate at KCL will submit Development Safety Update Reports (DSURs) to competent authorities.

### 6.11.4 Quality Assurance and Control

#### 6.11.4.1 Risk Assessment

QA is defined as all the planned and systematic actions established to ensure the trial is performed and data generated, documented and/or recorded and reported in compliance with the principles of GCP and applicable regulatory requirements. QC is defined as the operational techniques and activities performed within the QA system to verify that the requirements for quality of the trial related activities are fulfilled.

#### 6.11.4.2 Central Monitoring at KCL

KCL staff will review Case Report Form (CRF) data for errors and missing key data points. Essential trial issues, events and outputs, including defined key data points, will be detailed in the DESiGN trial Data Management Plan.

#### 6.11.4.3 On-site Monitoring

The frequency, type and intensity of routine and triggered on-site monitoring will be detailed in the DESiGN Quality Management and Monitoring Plan (QMMP). The QMMP will also detail the procedures for review and sign-off of monitoring reports. In the event of a request for a trial site inspection by any regulatory authority KCL must be notified as soon as possible.

##### 6.11.4.3.1 Direct access to clusters records

Participating investigators must agree to allow trial related monitoring, including audits, REC review and regulatory inspections, by providing access to source data and other trial related documentation as required. Cluster consent for this must be obtained as part of the registration process for the trial.

#### 6.11.4.4 Trial Oversight

Trial oversight is intended to preserve the integrity of the trial by independently verifying a variety of processes and prompting corrective action where necessary. The processes reviewed relate to enrolment, consent, eligibility, and allocation to trial groups; adherence to trial interventions and policies to protect participants, including reporting of harms; completeness, accuracy and timeliness of data collection; and will verify adherence to applicable policies detailed in the Compliance section of the protocol. Independent trial oversight complies with the KCL trial oversight policy.

In multi-centre trials this oversight is considered and described both overall and for each recruiting centre by exploring the trial dataset or performing site visits as described in the DESiGN Quality Management and Monitoring Plan.

##### 6.11.4.4.1 Trial Management Team

The Trial Management Team (TMT) will be set up to assist with developing the design, co-ordination and day to day operational issues in the management of the trial, including budget management. The membership, frequency of meetings, activity (including trial conduct and data review) and authority will be covered in the TMT terms of reference.

##### 6.11.4.4.2 Trial Management Group

A Trial Management Group (TMG) will be set up to assist with developing the design, co-ordination and strategic management of the trial. The membership, frequency of meetings, activity (including trial conduct and data review) and authority will be covered in the TMG terms of reference.

##### 6.11.4.4.3 Independent Trial Steering Committee/ Data Monitoring Committee

The Independent TSC/DMC is the only oversight body that has access to accumulating comparative data and the independent group responsible for oversight of the trial in order to safeguard the interests of trial participants. The TSC/DMC provides advice to the CI, KCL, the funder and sponsor on all aspects of the trial through its independent Chair. The membership, frequency of meetings, activity (including trial conduct and data review) and authority will be covered in the TSC/DMC terms of reference.

##### 6.11.4.4.4 Trial Sponsor

The role of the sponsor is to take on responsibility for securing the arrangements to initiate, manage and finance the trial.

# 7 Ethics and Dissemination

## 7.1 Research Ethics Approval

Before initiation of the trial at any clinical site, the protocol and any material to be advertised on the prospective clusters will be submitted to the relevant REC for approval. Any subsequent amendments to these documents will be submitted for further approval. Before initiation of the trial at each additional clinical site, the same/amended documents will be submitted for local Research and Development (R&D) approval.

The rights of the participant clusters to refuse to participate in the trial without giving a reason must be respected. After randomisation the clusters must remain within the trial for the purpose of follow up and data analysis according to the treatment option to which they have been allocated. However, the cluster remains free to change their mind at any time about the protocol treatment and follow-up without giving a reason.

Individual women participating in the trial have the opportunity to opt out from the trial so that their data is not used in this study. The Patient information sheet (PIS) has an opt-out section that participants can complete and return to the research team. This means women being cared in the participating Trusts will be deemed ‘opted in’ unless otherwise stated. The reasons for this are outlined in the section 7.5 of this protocol and this approach was approved by the Confidentiality Advisory Committee. Posters and opt-out leaflets (PIS) will be used to inform women about the study. The opt-out leaflets (PIS) will be available usually in the ultrasound unit, however this may vary to allow for local solutions.

## 7.2 Competent Authority Approvals

This is not a Clinical Trial of an Investigational Medicinal Product (IMP) as defined by the EU Directive 2001/20/EC. Therefore, a CTA is not required in the UK.

The progress of the trial, safety issues and reports, including expedited reporting of SUSARs, will be reported to the Competent Authority, regulatory agency or equivalent in accordance with relevant national and local requirements and practices.

## 7.3 Other Approvals

The protocol will be submitted by those delegated to do so to the relevant R&D department of each participating site or to other local departments for approval as required in each country. A copy of the local R&D approval (or other relevant approval as above), the PIS and individual consent form must be forwarded to the co-ordinating centre before participants are randomised to the trial. The PIS and the individual consent form are applicable only for the subsample of women participating in the process evaluation interviews. Detailed description of consent for the trial is described in section 7.5.

The protocol has received formal approval and methodological, statistical, clinical and operational input from the CCTU Protocol Review Committee.

## 7.4 Protocol Amendments

The Trial Team will be responsible to discuss any potential protocol amendment. They will also be discussed with the investigators, sponsors, KCL, patient groups (SANDS) or other organizations according to the content and how substantive the changes are. The final decision about any amendment will be from the CI and the Trial Team.

Amendments will be submitted for REC approval following local R&D approval. Additional approval from other regulatory agencies will be sought where appropriate. All protocol amendments will be communicated to the trial registries and relevant parties.

## 7.5 Consent or Assent

Investigators conducting individually randomised trials are required to obtain the informed consent of study participants before their random assignment. This ensures adequate explanation about potential risks of intervention and also facilitates the process of randomisation. In cluster randomised trials, however, the size of the cluster may impose a logistic limitation or even make it impossible to obtain individual informed consent and this must be outweighed against the nature of the intervention and potential associated risks (53).

In this cluster randomised trial not only the size of the clusters but an additional temporal issue makes impossible the individual informed consent. Before randomisation of the clusters, prospective women in the trial arm are unknown. Furthermore, the moment a woman presents at a cluster the allocation will have already been assigned. Therefore, the individual does not have the option to withdraw participation and avoid exposure to intervention. In this situation it has been reported the importance of stakeholders, community leaders, decision makers (lead from each cluster) and patient group engagement and agreement with the trial (53). Although it is not the same as individual informed consent, they will be the guardians of patients’ interest before and during the trial. This is in agreement with the MRC recommendations for cluster randomised trial which say that “the roles of the guardians of the patients interests during the trial, the gatekeepers of access to patient groups, and sponsors of the research are even more important in CRTs where individuals may not have the opportunity to give informed consent to participation” (54).

Additionally, is has been suggested the evaluation of a health care programme that is already accepted in clinical practice can be exempted from individual informed consent if clearly justified (55-57). GAP is currently being used in 105 (64%) of Trusts across England (31). Therefore, a harm of the intervention in the clinical detection of SGA should not be expected. As in any clinical trial, the TSC/DMC will also monitor this trial and safeguard the interest of the patient. They are allowed to stop the trial in the case of an unforeseen situation such as any harm related to the intervention.

In view of the above ethical consideration, this protocol has been discussed with key groups in UK. Letter of support from the following group are provided in this protocol: Strategic clinical network (SCN) (Appendix 4), Tommy’s (Appendix 5), PPI representative (Appendix 6), RCOG clinical study group on stillbirth (Appendix 7) and SANDS charity (Appendix 8). This trial protocol has been reviewed by the UK Confidentiality Advisory Group who have advised us on ways to minimise the use of identifiable information and agree that the minimal access to patient identifiable information is within the interests of the general public. We have also received a letter of support from NHS England who is currently developing the Stillbirth Care Bundle in which the GAP protocol is one of the four interventions proposed (Appendix 9). Local clinical leads in each cluster will sign the consent for on behalf of the individual participants. The cluster consent form is included in this protocol (Appendix 10).

Finally, the protection of individual participant identification and data has also been considered. This trial follows standard recommendation of confidentiality in which all patient identification will be kept locally. The trial data database and all information stored centrally will be linked to a unique PIN and will not include patient name, hospital number, NHS number or complete address. The trial database will be password protected and safely stored according to KCL procedures.

Individual consent will be obtained in a sample of women who will be approached for qualitative interviews on the acceptability of the GAP programme. These women will be provided with patient information leaflets and opportunity to consider the participation in the interviews for process evaluation will be provided. Hospital staff will be approached and provided with an information leaflet about the process evaluation study. Individual consent will be obtained from staff taking part in individual interviews.

### 7.5.1 Consent or Assent in Ancillary Studies

Not applicable.

## 7.6 Confidentiality

Participants will be given a unique trial PIN. Data will be entered under this identification number onto the central database stored on the servers based at KCL. The database will be password protected and only accessible to members of the DESiGN trial team at KCL, and external regulators if requested. The servers are protected by firewalls and are patched and maintained according to best practice. The physical location of the servers is protected by CCTV and security door access.

Individual data collected manually from clinical notes review will be entered directly into the trial database without any individual identification, such as name, hospital number or NHS number. Data from hospital electronic records will also be linked to a PIN. These data will then be anonymised and centralised at KCL in the trial database. At each local site there will be a record linking the PIN to the hospital ID. This will be stored locally. No patient identifiable data will be stored centrally.

## 7.7 Declaration of Interests

The investigators named on the protocol have no financial or other competing interests that impact on their responsibilities towards the scientific value or potential publishing activities associated with the trial.

## 7.8 Indemnity

KCL holds insurance to cover participants for injury caused by their participation in the clinical trial. Participants may be able to claim compensation if they can prove that KCL has been negligent. However, as this clinical trial is being carried out in a hospital, the hospital continues to have a duty of care to the participant in the clinical trial. KCL does not accept liability for any breach in the hospital’s duty of care, or any negligence on the part of hospital employees. This applies whether the hospital is an NHS Trust or not. This does not affect the participant’s right to seek compensation via the non-negligence route.

Participants may also be able to claim compensation for injury caused by participation in this clinical trial without the need to prove negligence on the part of KCL or another party. Participants who sustain injury and wish to make a claim for compensation should do so in writing in the first instance to the Chief Investigator, who will pass the claim to KCL’s insurers.

Hospitals selected to participate in this clinical trial shall provide clinical negligence insurance cover for harm caused by their employees and a copy of the relevant insurance policy or summary shall be provided to KCL, upon request.

## 7.9 Finance

The DESiGN trial have a phased funding strategy. Partial funding has initially been secured from Tommy’s Charity, grant number MQATAVR. Additional funding was secured from GST Charity (MAJ150704) and SANDS.

## 7.10 Access to Data

The CI and research team for data management and data analyses will have access to data from all clusters. Whilst patients will not be asked directly for their consent, they will be given the opportunity to opt-out of data collection, with notification via posters in the antenatal clinic or ultrasound departments. Datasets from all hospitals will be amalgamated by the research team and stored without any patient identification.

## 7.11 Ancillary and Post-trial Care

No additional care will be required as the individual trusts participating in the trial have undertaken the responsibility of the implementation of GAP in their unit and are committed to continue the use of GAP programme independently after this study. Participating trust may choose to modify their approach to the use of GAP programme following the study. This decision is independent of the trial and will be based on individual trusts clinical strategy.

## 7.12 Publication Policy

### 7.12.1 Trial Results

The results of the trial will be disseminated regardless of the direction of effect.

### 7.12.2 Authorship

The success of the trial depends on a large number of midwives, obstetricians and anaesthetists. Credit for the study findings will be given to all who have collaborated and participated in the study including all local co-ordinators and collaborators, members of the trial committees, KCL, and trial staff. Authorship at the head of any published paper will take the form “[name], [name] and [name] on behalf of The DESiGN Collaborative Group” and will include specific people involved in each piece of work and the co-investigators.

The writing of the primary results will be the responsibility of a writing committee including all of the investigators. All contributors to the study will be listed at the end of the report, with their contribution to the study identified. Decisions about authorship of additional papers will be discussed and agreed by the trial investigators and advice from the TSC/DMC will be requested if necessary.

### 7.12.3 Reproducible Research

The trial protocol will be published.

# 8 Ancillary Studies

None currently planned.

# 9 Protocol Amendments

| Protocol version | Date | Reason for update | Substantial amendment number | Summary of changes |
| --- | --- | --- | --- | --- |
| 5.0 | 01/09/2015 | Submitted to ethics | NA | Initial protocol. |
| 5.1 | 13/11/2015 | Clarification for ethics | NA | Description of support mechanisms in place for any women interviewed who become distressed |
| 5.1 | 16/05/2016 | Addition for trial documentations | 1 | Review of PIS and posters as per CAG recommendation |
| 5.1 | 06/07/2016 | Non-substantial amendment 2 | NA | Inclusion of 2 new sites. |
| 5.1 | 02/08/2017 | Substantial amendment 3 | 3 | Notification of change in study sponsor (from UCL to KCL). |
| 6.0 | 04/08/2017 | Change outcomes and amendment of PIS and consent forms. Also, update on participating sites, study timeline, data collection, TSC and DMC, planned secondary analysis, authorship, funders and trial registration number. | 4 | 1. Change of sponsorship and logos: This is due to a change in sponsorship and includes update off all trial documents (including PIS and posters). The change of sponsorship has now been approved (substantial amendment 3 - REC favourable opinion letter attached).  2. Trial registration: We included the trial registration and REC number in the protocol.  3. Change in outcomes: The primary outcome has been modified to the antenatal detection of SGA infants by customised and by population centiles, following discussion with the co-investigators, Perinatal Institute, funding agencies and independent reviewers. The rationale for the changes are based on the following: It is recognised that there is an overlap between SGA by customised and SGA by population; There is consensus that this group of infants have increased risk of adverse outcomes and evidence suggests these infants are at the highest risk of morbidity/mortality - detection of these babies are crucial; Both GAP and current practice are aimed at picking these babies - focusing on this overlap group addresses the issue that GAP was not developed to detect SGA pop infants (the opposite also applies - it addresses the issue that routine practice is not aimed at detecting SGA cust only); This has the advantage that there will be only one denominator and the use of this outcome makes clear that we are not comparing the definitions of SGA; Power calculation- based on data available from previous publications approximately 75% of SGA babies by population centiles are also SGA by customised (and vice versa – i.e 60% overlap by definitions). We have repeated the power calculation and are able to continue the study with current number of sites with greater than 80% power; It is important to note that the change in primary outcome does not result in a change in the inclusion or exclusion criteria, proposed study design, duration of study or data acquisition; The originally proposed primary outcome will now be evaluated as a secondary outcome.  4. Update personnel (investigators): The study statistician (Andrew Copas) is now included as a co-investigator. Change of site-investigators (Claire Rozette will be replaced by Spyrus Bakalis as Site PI for St Thomas’ Hospital; Bini Ajay will replace Rosol Hamid as Site PI for Croydon Hospital) and addition of new sites (Chesterfield Hospital and North Middlesex Hospital – HRA approval for their inclusion previously obtained). The list of site investigators and the trial management structure have been removed from the protocol so that future changes in personal does not require changes in the protocol.  5. Update study timeline: The new timeline has been approved by the funders (please see letter from funder). This includes changes in the study start and end date.  6. Amendment to consent and PIS: Clarification of access to data from regulatory agencies on informed consent (they were also removed from the protocol) and clarification of contact numbers in the PIS. In addition, study duration and contact information was clarified in the opt-out PIS.  7. Clarification on data collection (clinical outcomes): We clarified that data will be collected throughout the study. We are now planning to collect data retrospectively for a period immediately before the study to allow for understanding of trends of clinical outcomes and service provision. This will provide a more meaningful interpretation of the trial findings.  8. Joint TSC and DMC: We amended the structure of independent oversight of this study for a joint TSC and DCM. This is due to the nature of this study and the risk for participants deemed to be considerably low given the intervention is being used in clinical practice.  9. Secondary analysis: We provided a summary description of the aims of planned secondary analysis using collected data.  10. Authorship and report: We clarified authorship arrangements .  11. Update on funders and their logos: Additional funding was secured after initial REC application – we updated the funders and added their logos.  12. Process evaluation study: Updated data collection for process evaluation study – focus groups will not be performed and anticipation of the proposed time of interviews.  13. Health economics: We have updated the analysis plan for health economics, which will be focused in the cost-effectiveness (cost related to the detection of one small for gestational age infant).  14. Record keeping at participating sites: We clarified a delegation log will not be required locally as the majority of research activities is performed by the central research staff. Trial documents should be kept in a local file (to be created locally – not provided by the sponsor).  15. Statement of activities and schedule of events updated: Update of documents – KCL/GSTT (sponsor) will now use these documents as the agreement between site and sponsor. |
| 7.0 | 21/11/17 | 1. On reading the staff PIS, we realised that some of the information contained within the sheet is not appropriate for staff, and was likely written for the patient sheets initially. We have therefore removed this text. We have also edited the sheet so that there are now two versions – one for the early implementer site staff, the other for the delayed implementer site staff. Finally, we have added text to support follow-up questions, or telephone interviews, as per the changes approved in Amendment 4.  2.The staff and patient consent forms have been edited to reflect the new versions of the participant information sheets. | 5 | 1.Revised Staff Participant Information Sheets (PIS)  2.Revised consent forms |
| 7.0 | 21/11/17 | 1. Following discussions with our trial data management team, we have realised that the process of anonymising the patient data locally is complex. It is therefore not time efficient to request a clinician at each site to anonymise the data, nor is it reliable or likely to ensure consistency. We are concerned that errors at this stage will prevent us from making appropriate conclusions. We have therefore applied to the Confidentiality Advisory Group (CAG) to request permission for a member of the research team to have temporary access to the patient identifiable information, to perform the anonymization function. This is reflected in the revised protocol.  2.Due to stratification of trial financial resources, it may be necessary for a clinical fellow, rather than a research midwife, to perform the patient notes audits. We have therefore allowed for either role in the revised protocol.  3.We have edited the ‘opt-out’ participant information sheets to reflect the request made to CAG in changing the way that patient information is anonymised. | 6 | 1.Change to the process of anonymising the electronic patient data  2.Change to the job role of the person performing notes audits  3.Revised ‘Opt-out’ of research for patients |
| 8.0 | 16/8/18 | Non-cost extension approved for the trial meaning a change to the trial timeline | 7 | 1. Change to the trial timeline to reflect the non-cost extension to 30 November 2019. 2. Minor rephrasing of neonatal secondary outcomes 3. Updates to members of the trial steering and data monitoring committee. |

# 10 References

1. Chan AW, Tetzlaff JM, Altman DG, Laupacis A, Gotzsche PC, Krleza-Jeric K, et al. SPIRIT 2013 statement: defining standard protocol items for clinical trials. Annals of internal medicine. 2013;158(3):200-7.

2. Chan AW, Tetzlaff JM, Gotzsche PC, Altman DG, Mann H, Berlin JA, et al. SPIRIT 2013 explanation and elaboration: guidance for protocols of clinical trials. Bmj. 2013;346:e7586.

3. Froen JF, Cacciatore J, McClure EM, Kuti O, Jokhio AH, Islam M, et al. Stillbirths: why they matter. Lancet. 2011;377(9774):1353-66.

4. Pallotto EK, Kilbride HW. Perinatal outcome and later implications of intrauterine growth restriction. Clinical obstetrics and gynecology. 2006;49(2):257-69.

5. Gardosi J, Kady SM, McGeown P, Francis A, Tonks A. Classification of stillbirth by relevant condition at death (ReCoDe): population based cohort study. Bmj. 2005;331(7525):1113-7.

6. Gardosi J, Madurasinghe V, Williams M, Malik A, Francis A. Maternal and fetal risk factors for stillbirth: population based study. Bmj. 2013;346:f108.

7. Hepburn M, Rosenberg K. An audit of the detection and management of small-for-gestational age babies. Br J Obstet Gynaecol. 1986;93(3):212-6.

8. Kean LH, Liu DT. Antenatal care as a screening tool for the detection of small for gestational age babies in the low risk population. J Obstet Gynaecol 1996;16:77–82.

9. Lindqvist PG, Molin J. Does antenatal identification of small-for-gestational age fetuses significantly improve their outcome? Ultrasound in obstetrics & gynecology : the official journal of the International Society of Ultrasound in Obstetrics and Gynecology. 2005;25(3):258-64.

10. Battaglia FC, Lubchenco LO. A practical classification of newborn infants by weight and gestational age. J Pediatr. 1967;71(2):159-63.

11. Ota E, Ganchimeg T, Morisaki N, Vogel JP, Pileggi C, Ortiz-Panozo E, et al. Risk factors and adverse perinatal outcomes among term and preterm infants born small-for-gestational-age: secondary analyses of the WHO Multi-Country Survey on Maternal and Newborn Health. PLoS One. 2014;9(8):e105155.

12. Campbell MK, Ostbye T, Irgens LM. Post-term birth: risk factors and outcomes in a 10-year cohort of Norwegian births. Obstet Gynecol. 1997;89(4):543-8.

13. Gardosi J, Figueras F, Clausson B, Francis A. The customised growth potential: an international research tool to study the epidemiology of fetal growth. Paediatr Perinat Epidemiol. 2011;25(1):2-10.

14. Hutcheon JA, Zhang X, Cnattingius S, Kramer MS, Platt RW. Customised birthweight percentiles: does adjusting for maternal characteristics matter? BJOG : an international journal of obstetrics and gynaecology. 2008;115(11):1397-404.

15. Hutcheon JA, Zhang X, Platt RW, Cnattingius S, Kramer MS. The case against customised birthweight standards. Paediatr Perinat Epidemiol. 2011;25(1):11-6.

16. Zhang X, Platt RW, Cnattingius S, Joseph KS, Kramer MS. The use of customised versus population-based birthweight standards in predicting perinatal mortality. BJOG : an international journal of obstetrics and gynaecology. 2007;114(4):474-7.

17. Avcı ME, Sanlıkan F, Celik M, Avcı A, Kocaer M, Göçmen A. Effects of maternal obesity on antenatal, perinatal and neonatal outcomes. J Matern Fetal Neonatal Med. 2014:1-4.

18. Gardosi J, Clausson B, Francis A. The value of customised centiles in assessing perinatal mortality risk associated with parity and maternal size. BJOG : an international journal of obstetrics and gynaecology. 2009;116(10):1356-63.

19. Rossen LM. Neighbourhood economic deprivation explains racial/ethnic disparities in overweight and obesity among children and adolescents in the U.S.A. J Epidemiol Community Health. 2014;68(2):123-9.

20. Anderson NH, Sadler LC, Stewart AW, McCowan LM. Maternal and pathological pregnancy characteristics in customised birthweight centiles and identification of at-risk small-for-gestational-age infants: a retrospective cohort study. BJOG : an international journal of obstetrics and gynaecology. 2012;119(7):848-56.

21. Wood AM, Pasupathy D, Pell JP, Fleming M, Smith GC. Trends in socioeconomic inequalities in risk of sudden infant death syndrome, other causes of infant mortality, and stillbirth in Scotland: population based study. Bmj. 2012;344:e1552.

22. Vos AA, Posthumus AG, Bonsel GJ, Steegers EA, Denktas S. Deprived neighborhoods and adverse perinatal outcome: a systematic review and meta-analysis. Acta Obstet Gynecol Scand. 2014;93(8):727-40.

23. Villar J, Cheikh Ismail L, Victora CG, Ohuma EO, Bertino E, Altman DG, et al. International standards for newborn weight, length, and head circumference by gestational age and sex: the Newborn Cross-Sectional Study of the INTERGROWTH-21st Project. Lancet. 2014;384(9946):857-68.

24. Papageorghiou AT, Ohuma EO, Altman DG, Todros T, Cheikh Ismail L, Lambert A, et al. International standards for fetal growth based on serial ultrasound measurements: the Fetal Growth Longitudinal Study of the INTERGROWTH-21st Project. Lancet. 2014;384(9946):869-79.

25. Kierans WJ, Joseph KS, Luo ZC, Platt R, Wilkins R, Kramer MS. Does one size fit all? The case for ethnic-specific standards of fetal growth. BMC Pregnancy Childbirth. 2008;8:1.

26. Hargreaves K, Cameron M, Edwards H, Gray R, Deane K. Is the use of symphysis-fundal height measurement and ultrasound examination effective in detecting small or large fetuses? J Obstet Gynaecol. 2011;31(5):380-3.

27. Goto E. Prediction of low birthweight and small for gestational age from symphysis-fundal height mainly in developing countries: a meta-analysis. J Epidemiol Community Health. 2013;67(12):999-1005.

28. Gardosi J, Francis A. Controlled trial of fundal height measurement plotted on customised antenatal growth charts. Br J Obstet Gynaecol. 1999;106(4):309-17.

29. Roex A, Nikpoor P, van Eerd E, Hodyl N, Dekker G. Serial plotting on customised fundal height charts results in doubling of the antenatal detection of small for gestational age fetuses in nulliparous women. Aust N Z J Obstet Gynaecol. 2012;52(1):78-82.

30. Royal College of Obstetricians and Gynaecologists (RCOG). Small-for-Gestational-Age Fetus, Investigation and Management. Green-top Guideline No. 31 (2nd edn). RCOG Press: London, 2013.

31. Perinatal Institute. *Growth Assessment Protocol (GAP) - Uptake of the GAP Programme in the UK*. <https://www.perinatal.org.uk/gap-uptake.aspx> (accessed 30 June 2015).

32. Gardosi J, Giddings S, Clifford S, Wood L, Francis A. Association between reduced stillbirth rates in England and regional uptake of accreditation training in customised fetal growth assessment. BMJ Open. 2013;3(12):e003942.

33. Hill AB. The Environment and Disease: Association or Causation? Proceedings of the Royal Society of Medicine. 1965;58:295-300.

34. Carberry AE, Gordon A, Bond DM, Hyett J, Raynes-Greenow CH, Jeffery HE. Customised versus population-based growth charts as a screening tool for detecting small for gestational age infants in low-risk pregnant women. Cochrane Database Syst Rev. 2014;5:CD008549.

35. Office for National Statistics. Characteristics of birth 1, England and Wales, 2013. London: ONS, 2014. Available at: <http://www.ons.gov.uk/ons/rel/vsob1/characteristics-of-birth-1--england-and-wales/2013/index.html> (accessed 28 January 2015).

36. General Register Office for Scotland. Vital events reference tables. Edinburgh: GROS, 2013. Available at: <http://www.gro-scotland.gov.uk/statistics/theme/vital-events/general/ref-tables/2013/section-1-summary.html> (accessed 28 January 2015).

37. Smith GCS, Fretts RC. Stillbirth. The Lancet. 2007;370(9600):1715-25.

38. Mondal D, Galloway TS, Bailey TC, Mathews F. Elevated risk of stillbirth in males: systematic review and meta-analysis of more than 30 million births. BMC Med. 2014;12:220.

39. Perinatal Institute. *Growth Assesment Programme (GAP): Outline Specification.* <http://www.perinatal.org.uk/FetalGrowth/PDFs/GROW_Programme_2014_New_Units.pdf> (accessed 11 March 2015).

40. Confidential enquiry into stillbirths with intrauterine growth restriction. Perinatal Institute, [www.pi.nhs.uk/rpnm/CE_SB_Final.pdf;](http://www.pi.nhs.uk/rpnm/CE_SB_Final.pdf;) 2007. (accessed 02 January 2015).

41. Smith, G. (2015, April). *Fetal Growth - What is Normal, What is Impaired & How do we Test for it?* PowerPoint presentation at the annual conference of the British Maternal & Fetal Medicine Society, London, UK.

42. Clausson B, Gardosi J, Francis A, Cnattingius S. Perinatal outcome in SGA births defined by customised versus population-based birthweight standards. BJOG : an international journal of obstetrics and gynaecology. 2001;108(8):830-4.

43. Gardosi J, Francis A. Adverse pregnancy outcome and association with small for gestational age birthweight by customized and population-based percentiles. Am J Obstet Gynecol. 2009;201(1):28 e1-8.

44. Ego A, Subtil D, Grange G, Thiebaugeorges O, Senat MV, Vayssiere C, et al. Customized versus population-based birth weight standards for identifying growth restricted infants: a French multicenter study. Am J Obstet Gynecol. 2006;194(4):1042-9.

45. Lajos GJ, Haddad SM, Tedesco RP, Passini R, Jr., Dias TZ, Nomura ML, et al. Intracluster correlation coefficients for the Brazilian Multicenter Study on Preterm Birth (EMIP): methodological and practical implications. BMC medical research methodology. 2014;14:54.

46. Anderson R. New MRC guidance on evaluating complex interventions. Bmj. 2008;337:a1937.

47. Moore G, Audrey S, Barker M, Bond L, Bonell C, Cooper C, et al. Process evaluation in complex public health intervention studies: the need for guidance. J Epidemiol Community Health. 2014;68(2):101-2.

48. Steckler A, Linnan L. (2002) Process evaluation for public health interventions and research , J Wiley, San Franciso.

49. Hoffmann TC, Glasziou PP, Boutron I, Milne R, Perera R, Moher D, et al. Better reporting of interventions: template for intervention description and replication (TIDieR) checklist and guide. Bmj. 2014;348:g1687.

50. Peto R, Pike MC, Armitage P, Breslow NE, Cox DR, Howard SV, et al. Design and analysis of randomized clinical trials requiring prolonged observation of each patient. I. Introduction and design. Br J Cancer. 1976;34(6):585-612.

51. Geller NL, Pocock SJ. Interim analyses in randomized clinical trials: ramifications and guidelines for practitioners. Biometrics. 1987;43(1):213-23.

52. Torloni MR, Vedmedovska N, Merialdi M, Betran AP, Allen T, Gonzalez R, et al. Safety of ultrasonography in pregnancy: WHO systematic review of the literature and meta-analysis. Ultrasound in obstetrics & gynecology : the official journal of the International Society of Ultrasound in Obstetrics and Gynecology. 2009;33(5):599-608.

53. Donner A, Klar N. Pitfalls of and controversies in cluster randomization trials. Am J Public Health. 2004;94(3):416-22.

54. Medical Research Council*. Cluster Randomised Trials: Methodological and Ethical Considerations*. London, England: Medical Research Council; 2002.

55. Goldberg HI, McGough H. The ethics of ongoing randomization trials. Investigation among intimates. Medical care. 1991;29(7 Suppl):JS41-8.

56. Henderson WG, Demakis J, Fihn SD, Weinberger M, Oddone E, Deykin D. Cooperative studies in health services research in the Department of Veterans Affairs. Controlled clinical trials. 1998;19(2):134-48.

57. Winkens RA, Knottnerus JA, Kester AD, Grol RP, Pop P. Fitting a routine health-care activity into a randomized trial: an experiment possible without informed consent? Journal of clinical epidemiology. 1997;50(4):435-9.

# 11 Appendices

## 11.1 Appendix 1. GAP programme description

**Growth Assessment Protocol (GAP):**

**Outline Specification**

**INTRODUCTION AND BACKGROUND**

Fetal growth restriction (FGR) is associated with stillbirth, neonatal death and perinatal morbidity. Confidential Enquiries have demonstrated that most stillbirths due to fetal growth restriction are associated with suboptimal care and are potentially avoidable. A recent epidemiological analysis based on the comprehensive West Midlands database has underlined the impact that fetal growth restriction has on stillbirth rates, and the significant reduction which can be achieved through antenatal detection of pregnancies at risk. Customised assessment of birthweight and fetal growth has also been recommended by the RCOG since 2002 and is re-emphasised in the 2013 revision of the Green Top Guidelines.

The Perinatal Institute (PI) provides tools for assessment of fetal growth and birth weight by defining each pregnancy’s growth potential through the Gestation Related Optimal Weight (GROW) software, including

- GROW-chart: customised antenatal charts for plotting fundal height and estimated fetal weight.
- GROW-centile: for calculation of customised birthweight centiles - as an individual centile calculator, or as a bulk centile calculator for databases of pregnancies

The software for these applications has been freely available and used in a variety of settings, and are currently already in use in over 88 trusts and health boards in the NHS as a web application. However recently completed audits in the West Midlands have shown that antenatal detection of fetal growth restriction is directly related to the degree of training and implementation of standardised, evidence based protocols. Therefore from 2013/14, continued or new provision of the software will require Trusts to be accredited in the Growth Assessment Protocol (GAP) Programme. This includes comprehensive staff training, monitoring of FGR referral / detection rates, and regular audits of FGR cases not antenatally detected to help identify system failures in fetal growth surveillance. The GAP programme has resulted in significant reductions in stillbirths in each of the NHS regions where it was widely implemented and has been associated with recent year on year drops in national stillbirth rates in England, to their lowest levels. These successes have been recognised by successive Patient Safety Awards for the Perinatal Institute team in 2013 and 2014.

**This document** outlines the service specification and agreement the Perinatal Institute proposes to enter with your Trust, with respective roles and responsibilities. It is based on three main elements;

1. Training and accreditation of all staff involved in clinical care
2. Adoption of evidence based protocols and guidelines
3. Rolling audit and benchmarking of performance

**GENERAL**

The GROW Team at PI would like to establish regular communication with nominated ‘link persons’ in each specialty, including midwifery (e.g. HOM, clinical risk manager, matron); obstetrics / MFM, ultrasound and IT. These links are intended to serve as conduits for regular communication and feedback on progress.

1. **TRAINING**

*Rationale: Fetal growth restriction is one of the most common complications in pregnancy. Alongside many competing priorities, competency in fetal growth assessment is essential to ensure clinical alertness and ability to make the expectant mother aware that her baby is at increased risk because of suboptimal fetal growth. Standardised assessment improves detection and reduces unnecessary investigations.*

Aim: all maternity care providers who are engaged in maternity care to receive instruction on

- awareness of risk factors for FGR and perinatal mortality, including medical, social and obstetric history
- principles and use of customised charts
- standardised fundal height measurement and recording on the GROW chart
- clinical implications and referral pathways

Roles and Responsibilities

PI: - will provide latest updates of the GROW software (stand-alone or linked to the Trust’s maternity information system) together with ongoing helpdesk support

- rolling programme of training workshops at the PI for GROW link persons / trainers (dates available at <http://www.perinatal.org.uk/diary/diary.aspx>)

- provide a GAP e-learning package and competency document to assess trained staff

Trust: - ensures GROW link persons / trainers attend annual ‘train the trainers’ workshops at the PI - all staff engaged in maternity care and their supervisors are trained

- ensures competency of staff is assessed

- maintains training and competency log

- all staff complete e-learning package and assessment on an annual basis

1. **PROTOCOLS**

*Rationale: There is currently a wide variation in protocols for risk assessment, fetal growth surveillance and referral pathways. This is often accompanied by insufficient investigations for at-risk pregnancies as a result of real or perceived shortages in ultrasound services. New national guidelines present an opportunity to implement standardised, evidence based protocols.*

Aim: To assist with the implementation of

- risk assessment and definition of low and high risk care pathways at booking / early pregnancy
- indications for serial scans and protocols for frequency and timing
- indications for referral for further investigations / obstetric review where required

Roles and Responsibilities

PI: - will provide template protocols representing the latest evidence for surveillance, referral and investigation of pregnancies suspected of fetal growth problems

Trust: - will agree a Trust wide policy which is consistent with such guidelines

- will monitor and ensure that these are adhered to through regular audit (see 3.)

NB protocols are not intended to replace clinical considerations in the management of individual pregnancies.

1. **AUDIT**

*Rationale: Region wide experience in the West Midlands has shown that ‘antenatal detection’ of the SGA baby is an auditable indicator and collection of this information itself promotes learning opportunities and improvement.*

Aim: To establish a rolling audit programme to monitor performance, through

- the SGA / FGR rate (proportion of babies born with a birthweight below the 10^th^ customised centile)
- rate of antenatal referral for suspected SGA / FGR and antenatal detection/diagnosis of SGA
- regular case-note audit of SGA / FGR cases that were not antenatally detected and action plans in response to system failures

Roles and Responsibilities

PI: - will provide data capture tool to calculate the customised birthweight centile and record antenatal detection of abnormal growth as an integral part of the GROW software

- will provide quarterly reports to feed back and benchmark performance

- will provide a tool and training for case note audit of SGA / FGR cases not antenatally detected

Trust: - will record a customised birthweight centile for each baby

- will record baseline and ongoing referral and detection rates of abnormal growth and set Trust specific targets

- undertake a quarterly case note audit and review of at least 10 SGA / FGR cases not antenatally detected

**4. ANNUAL COST**

Charges for the Growth Assessment Programme for new GROW users have been calculated on a minimum cost basis and stratified according to number of deliveries. Payment of set-up and pro-rata first-year costs, are due on commencement of training.

| **Size of Trust**  births per annum | **Set up cost**  Incl. training | **Annual Cost** from 2015/16 |
| --- | --- | --- |
| <3000 | £ 500 | £ 1500 |
| 3000-5000 | £ 500 | £ 2000 |
| 5000-7000 | £ 500 | £ 3000 |
| > 7000 | £ 500 | £ 4000 |

**5. PAYMENT PROCESS**

Details for purchase order

Supplier:

Perinatal Institute,
75 Harborne Road, Birmingham B15 3BU
Company Reg: 08466773
VAT: 161-7845-91

Bank:

Perinatal Institute,
NatWest Bank,
Edgbaston
Sort Code: 60-07-41
Account: 51150158

Please return purchase order together with completed Service Agreement, via

E-mail: [grow@perinatal.org.uk](mailto:grow@perinatal.org.uk);
Fax: 0121 607 0102; or
Post: Perinatal Institute
 75 Harborne Road, Edgbaston,
 Birmingham B15 3BU.

## 11.2 Appendix 2. TIDieR checklist


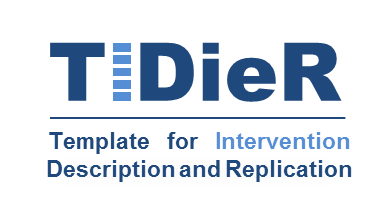
**The TIDieR (Template for Intervention Description and Replication) Checklist*:**

Information to include when describing an intervention and the location of the information

| **Item number** | **Item** | **Where located **** | |
| --- | --- | --- | --- |
|  |  | Primary paper  (page or appendix  number) | Other ^†^ (details) |
|  | **BRIEF NAME** |  |  |
| **1.** | Provide the name or a phrase that describes the intervention. | __27________ | ______________ |
|  | **WHY** |  |  |
| **2.** | Describe any rationale, theory, or goal of the elements essential to the intervention. | __20-22_____ | _____________ |
|  | **WHAT** |  |  |
| **3.** | Materials: Describe any physical or informational materials used in the intervention, including those provided to participants or used in intervention delivery or in training of intervention providers. Provide information on where the materials can be accessed (e.g. online appendix, URL). | __27-31_____ | _____________ |
| **4.** | Procedures: Describe each of the procedures, activities, and/or processes used in the intervention, including any enabling or support activities. | __27-31_____ | _____________ |
|  | **WHO PROVIDED** |  |  |
| **5.** | For each category of intervention provider (e.g. psychologist, nursing assistant), describe their expertise, background and any specific training given. | __27 and 28_ | _____________ |
|  | **HOW** |  |  |
| **6.** | Describe the modes of delivery (e.g. face-to-face or by some other mechanism, such as internet or telephone) of the intervention and whether it was provided individually or in a group. | __27________ | _____________ |
|  | **WHERE** |  |  |
| **7.** | Describe the type(s) of location(s) where the intervention occurred, including any necessary infrastructure or relevant features. | __27_______­_ | _____________ |
|  | **WHEN and HOW MUCH** |  |  |
| **8.** | Describe the number of times the intervention was delivered and over what period of time including the number of sessions, their schedule, and their duration, intensity or dose. | __27_______­­_ | _____________ |
|  | **TAILORING** |  |  |
| **9.** | If the intervention was planned to be personalised, titrated or adapted, then describe what, why, when, and how. | __27________ | _____________ |
|  | **MODIFICATIONS** |  |  |
| **10.^ǂ^** | If the intervention was modified during the course of the study, describe the changes (what, why, when, and how). | _not applicable_ | _____________ |
|  | **HOW WELL** |  |  |
| **11.** | Planned: If intervention adherence or fidelity was assessed, describe how and by whom, and if any strategies were used to maintain or improve fidelity, describe them. | __31 and 41___ | _____________ |
| **12.^ǂ^** | Actual: If intervention adherence or fidelity was assessed, describe the extent to which the intervention was delivered as planned. | __31 and 41___ | _____________ |

** **Authors** - use N/A if an item is not applicable for the intervention being described. **Reviewers** – use ‘?’ if information about the element is not reported/not sufficiently reported.

† If the information is not provided in the primary paper, give details of where this information is available. This may include locations such as a published protocol or other published papers (provide citation details) or a website (provide the URL).

ǂ If completing the TIDieR checklist for a protocol, these items are not relevant to the protocol and cannot be described until the study is complete.

* We strongly recommend using this checklist in conjunction with the TIDieR guide (see *BMJ* 2014;348:g1687) which contains an explanation and elaboration for each item.

* The focus of TIDieR is on reporting details of the intervention elements (and where relevant, comparison elements) of a study. Other elements and methodological features of studies are covered by other reporting statements and checklists and have not been duplicated as part of the TIDieR checklist. When a **randomised trial** is being reported, the TIDieR checklist should be used in conjunction with the CONSORT statement (see [www.consort-statement.org](http://www.consort-statement.org)) as an extension of **Item 5 of the CONSORT 2010 Statement.** When a **clinical trial** **protocol** is being reported, the TIDieR checklist should be used in conjunction with the SPIRIT statement as an extension of **Item 11 of the SPIRIT 2013 Statement** (see [www.spirit-statement.org](http://www.spirit-statement.org)). For alternate study designs, TIDieR can be used in conjunction with the appropriate checklist for that study design (see [www.equator-network.org](http://www.equator-network.org)).

## 11.3 Appendix 3. Minimum requirements for GAP compliance in the DESiGN trial.

**Growth Assessment Protocol (GAP) Accreditation**– to follow demonstrable implementation of Training, Audit process and Protocols

**1**. **Multidisciplinary local GAP team identified**

- including obstetrician, midwife and ultrasonographer
- links with Perinatal Institute support team
- ensures implementation and Trust ownership of the GAP programme

**2. Training:**

Content to include
• Awareness of risk factors for FGR and perinatal mortality, including medical, social and obstetric history
• Principles and evidence for use of customised charts
• Standardised fundal height measurement
• Recording on GROW charts including referral guidelines

Completion

At least 75% of staff engaged in maternity care -

- have received face to face training on GAP including
  - principles and rationale
  - fundal height measurement
  - use of customised charts
- completed competency assessment
- completed the e-learning package
- Training log of all staff is monitored and maintained
- On-going training of GAP elements is included in Trust Training Needs Analysis (TNA)

**3. Audit:**

Content to include:

- Baseline audit to determine rates of SGA, referral and detection
- Use of GROW application to produce centiles and referral and detection rates
- Missed case audit to examine cases with unrecognised SGA

Completion:

- 3+ months of annual deliveries baseline audit of detection rates completed using the GROW tool
- Process in place to record birthweight and referral & detection after each birth
- Tool in place and staff assigned and trained in missed case audit tool and process

**4. Unit or Trust Protocol:**

Content to include

- Risk assessment and definition of low and increased risk care pathways at booking / early pregnancy
- Indications for serial scans and protocols for frequency and timing
- Indications for referral for further investigations / obstetric review where required
- Risk assessment at booking/early pregnancy according to NHS England FGR algorithm.

Completion:

Evidence that GAP template protocol tailored to local use and implemented

## 11.4 Appendix 4. Letter of support from SCN

## 11.5 Appendix 5. Letter of support from Tommy’s Charity.

## 11.6 Appendix 6. Letter of support from PPI representative.

## 11.7 Appendix 7. Letter of support from RCOG clinical study group on stillbirth.

## 11.8 Appendix 8. Letter of support from SANDS charity.

## 11.9 Appendix 9. Letter of support from NHS England.

## 11.10 Appendix 10. Cluster consent form from local clinical leads.

Dr Dharmintra Pasupathy MSc PhD MRCOG

Senior Lecturer / Consultant in Maternal & Fetal Medicine

and Perinatal Epidemiology

Division of Women's Health

Women's Health Academic Centre KHP

10th Floor North Wing

St. Thomas' Hospital

Westminster Bridge Road

London SE1 7EH

*DATE*

Dear Dr Pasupathy

**Re: Invitation to participate in the DESiGN Trial**

Thank you for the invitation to participate in the DESiGN Trial, a randomised controlled cluster trial.

On behalf of our maternity unit and clinical director of maternity services, *PLEASE ADD NAME OF HOSPITAL* we agree to participate in this trial. This trial has been discussed in our unit and I *PLEASE ADD YOUR NAME* am the nominated clinical link in our unit for this study.

Our participation is based on the consensus that this is an area that needs to be robustly evaluated through a randomised controlled trial before implementation. An understanding of the impact on detection, clinical outcomes and service provision is crucial. We understand the study design and the implication of our participation.

We acknowledge and agree that based on the nature of this study, hospitals will be randomised to either early or delayed implementation of GAP. We understand that consent for participation of this study is at the level of the maternity unit and not individual patient based consent.

Best wishes

YOUR NAME & SIGNATURE
