# Supplementary material for: Evaluation of the Growth Assessment Protocol (GAP) for antenatal detection of small for gestational age: The DESiGN cluster randomised trial
Source: PLoS Med. 2022 Jun 21;19(6):e1004004. doi: 10.1371/journal.pmed.1004004 (PMC9212153; doi:10.1371/journal.pmed.1004004)
Supplement: S1 Appendix — (DOCX) [file pmed.1004004.s002.docx]

DEtection of Small for GestatioNal age fetus (SGA) – a cluster randomised controlled trial to evaluate the effect of the Growth assessment protocol (GAP) programme: The DESiGN Trial

Protocol version: 7 dated 18/01/2018

International Standard RCT Number: ISRCTN67698474

**Statistical Analysis Plan (SAP)**

**Version 0**

| **Authorised by:** | **Signature** | **Date** |
| --- | --- | --- |
| Dr Dharmintra Pasupathy, Chief Investigator  Email: dharmintra.pasupathy@kcl.ac.uk |  |  |
| Dr Andrew Copas, Trial Statistician  Email: a.copas@ucl.ac.uk |  |  |

**Table of contents**

[1 ABBREVIATIONS 3](#_Toc491072804)

[2 Abstract – Background and design 4](#_Toc491072805)

[3 Outcome measures 5](#_Toc491072806)

[3.1 Primary outcome 5](#_Toc491072807)

[3.2 Secondary outcomes 5](#_Toc491072810)

[4 Data 7](#_Toc491072817)

[4.1 Data collection and management 7](#_Toc491072818)

[4.2 Data verification 7](#_Toc491072819)

[4.3 Baseline and main trial periods 7](#_Toc491072820)

[5 Sample size estimation 8](#_Toc491072824)

[5.1 Primary outcome 8](#_Toc491072825)

[5.2 Secondary outcomes 9](#_Toc491072826)

[6 Analysis Principles 10](#_Toc491072827)

[6.1 Analysis population: Intention-to-treat (ITT), per-protocol, or other? 10](#_Toc491072828)

6.2 Significance level of tests

[6.3 Baseline comparability 10](#_Toc491072830)

[6.4 Adjustment for design factors 10](#_Toc491072831)

[6.5 Losses to follow-up: handling missing data 10](#_Toc491072832)

[6.6 Cluster level analysis – choice of effect measure and use of baseline data 10](#_Toc491072833)

[7 Analysis details 11](#_Toc491072834)

[7.1 Recruitment and follow-up patterns 11](#_Toc491072835)

[7.2 Participant Characteristics 11](#_Toc491072836)

[7.3 Analysis methods 11.](#_Toc491072838)

[7.3.1 Primary outcome: detail of the two stage cluster-summary method 11](#_Toc491072839)

[7.3.2 Secondary outcomes 11](#_Toc491072840)

[7.4 Sensitivity analyses 12](#_Toc491072844)

[7.5 Subgroup analyses 12](#_Toc491072846)

7.6 Adjustment for factors in analysis

[7.7 Regression diagnostics 12](#_Toc491072847)

[7.8 Multiple imputation by chained equations (MICE) 12](#_Toc491072851)

[8 Tables and graphs 1](#_Toc491072853)3

[8.1 Tables 1](#_Toc491072854)3

[8.2 Graphs 1](#_Toc491072855)3

[9 References 1](#_Toc491072856)4

# ABBREVIATIONS

| **Acronyms** | **Meaning** |
| --- | --- |
| ANCOVA | Analysis of covariance |
| CI | Confidence Interval |
| ITT | Intention-To-Treat |
| MAR | Missing At Random |
| MI | Multiple Imputation |
| MICE | Multiple Imputation by Chained Equations |
| OR | Odds Ratio |
| SAP | Statistical Analysis Plan |
| SGA | Small for gestational age |

# Abstract – Background and design

**Aim and objectives:** The objectives for this cluster randomised trial are to: i) determine whether implementation of the GAP programme will result in an improved detection of SGA by ultrasound; ii) investigate the effect of the intervention on short-term maternal and neonatal outcomes; iii) estimate the impact of GAP on clinical service provision and health economics and iv) assess fidelity and quality of implementation, acceptability and identify contextual factors associated with variation in the effect of GAP.

**Primary Outcome**

The primary outcome of this study is antenatal ultrasound detection of SGA (after 24 completed weeks of gestation) in infants who are SGA at birth by both population and customised centiles. See later for definitions.

**Secondary Outcomes**

The key secondary outcomes are classified according to: clinical outcomes, health-economic outcomes and outcomes derived from process evaluation of the programme implementation. This analysis plan is concerned with the clinical outcomes which are tabulated later.

**Population studied:** Inclusion and exclusion criteria:

Maternity units in the UK are eligible unless they have already implemented GAP. Within participating units all women who give birth in the study periods are eligible for the study and the intervention is implemented at the unit level, but data from women with multiple pregnancies or fetal congenital abnormalities will be excluded from the analysis.

**Trial design:** This trial is a cluster randomised trial where each cluster also provides baseline data (on different individuals). Clusters are randomised to the GAP programme or standard care.

The study intervention is implementation of the GAP programme. This includes cascading staff training, adopting or refining evidence-based protocols for SGA detection, routine monitoring of SGA and detection rates, regular audits of missed cases and ongoing support between the Perinatal Institute and Trusts. In the standard care arm, women will receive routine care as per their current hospital practice on screening and management of SGA.

Most clinical outcome data will be acquired from routine hospital systems. These will be collected for each cluster for a baseline period before clusters have begun implementing GAP and a main trial period in which those clusters allocated to GAP have fully implemented it. See later for details.

**Sample size:** The target sample size is a minimum of 12 clusters, 6 allocated to each arm. We anticipate collecting data from between 84-126 babies with SGA at birth by both population and customised centiles in the main trial period from each cluster, and data for at least as many in the baseline period. This provides between 79% and 84% power to detect as significant our targeted increase in the primary outcome due to the intervention from 20 to 33%.

**Randomisation:** The first 8 clusters committing to participate were divided into two strata of 4 clusters each according to their size (number of deliveries during the year 2013-2014). Two further strata (of three and then two) clusters were subsequently defined for clusters that agreed to participate in the study at later dates. Within each stratum allocation was by computerised random permutation.

**Blinding:**

The clinical outcome data is mainly collected from routine data within each trust. The data collection will occur after allocation. No blinding is possible either during implementation or during statistical analysis.

# Outcome measures

## Primary outcome

The primary outcome of this study is antenatal ultrasound detection of SGA (after 24 completed weeks of gestation) in infants who are SGA at birth. In this trial, we are focused on the antenatal detection of those infants who weigh less than the 10^th^ centile for gestational age on both population-derived and customised growth charts at birth. This group of infants will be the denominator (SGA at birth) for the estimation of the detection rate. Amongst these infants, the numerator (antenatal ultrasound detection of SGA) will be defined as ultrasound-derived estimated fetal weight <10^th^ centile by customised charts in the GAP implementation arm and by population charts in the standard care arm.

## Secondary outcomes

The table below describes all secondary outcomes for the trial, but this plan is only concerned with clinical outcomes. Note that of these those with a double asterisk ** are not to be reported in the first clinical paper.

Table: Key secondary outcomes

| **Clinical Outcomes** | | | **Health-economic outcomes** | **Process evaluation of implementation** |
| --- | --- | --- | --- | --- |
| Antenatal assessments | Neonatal outcomes | Maternal outcomes |  |  |
| Rate of antenatal ultrasound detection of SGA at birth by customised standards and by population references.  Antenatal clinical detection* of SGA**.  Analysis of GAP diagnostic test performance (specificity, sensitivity, negative predictive value, positive predictive value)**.  Ultrasound assessment of SGA using a different threshold e.g. 5^th^ centile**.  Growth trajectories (fetal biometry and EFW) and Doppler parameters in the detection of SGA**.  Comparison of GROW ultrasound charts against standard population charts on classification of fetal growth (small for gestational age, appropriate for gestational age, large for gestational age)**. | **Basic parameters**:  Gestational age at birth  Birthweight  Head circumference | **Antenatal:**  Length of stay in hospital | Number of ultrasound scans after 24 weeks  Antenatal clinic / antenatal day unit activity  Rates of induction of labour.  Rates of caesarean sections  Length of maternal and neonatal stay  Admissions and average length of stay in NICU/ SCBU | Proportion of staff trained, staff assessed and  women assessed with GAP  programme.  Adherence to SGA risk stratification and management protocols  Adherence to missed case analysis  Evaluation of acceptability and feasibility of intervention to staff and women, contextual barriers and facilitators, practice in control sites  Organisational impact and unintended consequences |
|  | **Condition at birth:**  5-minute Apgar score <7  Arterial cord pH <7.1  Any respiratory support given at delivery | **Intrapartum:**  Induction of labour  Mode of delivery (including Caesarean section rates)  Postpartum haemorrhage  Rates of 3^rd^ or 4^th^ degree perineal tear |  |  |
|  | **Neonatal admissions:**  Length of stay at each neonatal level of care |  |  |  |
|  | **Neonatal morbidity:**  Major neonatal morbidity (Any of neonatal brain injury, receipt of supplemental oxygen at 28 days of age, Bell stage 2+ necrotising enterocolitis, Culture-positive sepsis, retinopathy requiring ophthalmic intervention).  Minor neonatal morbidity (Any of: hypothermia, hypoglycaemia, nasogastric tube feeding) | **Postnatal:**  Length of stay in hospital  Breastfeeding at discharge |  |  |
|  | **Perinatal loss:**  Antepartum or intrapartum stillbirth  Neonatal death (early or late)  Death before neonatal discharge (after 28 days of birth)  Cause of death. |  |  |  |
| Abbreviations: EFW – estimated fetal weight, GROW – gestation-related optimal weight, NICU – neonatal intensive care unit, SCBU – special care baby unit, SGA – small for gestational age.  *Clinical detection of SGA is defined as ‘antenatal acknowledgement that the fetus is expected to weigh below the 10^th^ centile at birth, by charts appropriate to the study arm’.  **These secondary outcomes will not be reported in the first clinical paper, please see the section on presentation and publication strategy. | | | | |

# Data

## Data collection and management

Most data will be acquired from routine hospital systems. Data obtained either manually from clinical notes, or electronically from hospital maternity and neonatal databases will be entered/uploaded into the study database. Data will be pseudonymised and linked to a study identification number at each site, before the pseudonymised data is sent electronically to the trial team.  The key connecting participant details to study identification number will be password-protected and kept locally at study sites on NHS networks.

Data collected during the study includes clinical data from the period prior to implementation (baseline data) and the rates of training compliance. We will also use a calculator provided by the Perinatal Institute, which determines customised birthweight standards. A review of the fidelity of GAP use will inform data monitoring and the implementation evaluation, and clinical and service use data will be collected for a 4-6-month period after full GAP implementation, to assess primary and secondary outcomes of this study.

For the process evaluation of implementation, quantitative data (including proportion of staff trained/assessed, women managed with the GAP programme, missed case audit) will be collected.

## Data verification

Basic data checks are performed by the data manager periodically during the trial. Additional range, consistency and missing data checks will be performed by the statistician when the datasets for analysis are constructed, as appropriate, before the statistical analysis is performed. All variables will be examined for unusual, outlying, unlabelled or inconsistent values.

Any problems with trial data will be queried with the Trial Manager or Data Manager as appropriate. If possible, data queries will be resolved; although it is accepted that due to administrative reasons and data availability a small number of problems will continue to exist. These will be minimised.

## Baseline and main trial periods

Although it would be simplest and most robust to define common baseline and main periods for collection of birth outcomes for all trusts this is not possible in this trial because of the different dates at which trusts could commit to the trial and also because of different times taken to implement GAP in trusts in the intervention arm.

The baseline period is the twelve months 10/3/16-9/3/17 (defined because the earliest trust to start training for the intervention was randomised in the second wave and began on 10/3/17) except for the last stratum to be randomised (two trusts) where it is 19/12/16-18/12/17 (defined because the one intervention arm trust began training on 19/12/17).

The main or ‘follow up’ period is defined to be the six months 1/9/18-28/2/19 for all trusts except if:

- 1. an intervention trust that fully implemented the intervention later than the planned deadline of 01/03/18 in which case to reduce contamination the period is reduced to the final 4 months 1/11/18-28/2/19, or
  2. a control trust that is unwilling to wait until after February 2019 to start implementing, in which case the comparison period is the 6 months before they start implementation.

# Sample size estimation

## Primary outcome

A minimum target sample size of 12 clusters (6 per arm) was set based on information collected during protocol development. First, we assessed that the mean births per cluster per year in a sample of London maternity Trusts likely to participate in the trial was 5053. We assumed a 10% rate of SGA by a single definition. Pooled estimates from previous studies suggest that 75% of SGA infants defined by customised standards are also SGA by population references and vice versa. Therefore, we estimate an SGA rate by either definition (customised or population) of 12.5% and 60% of these babies (7.5% of total sample) will be SGA by both definitions (Figure 4).

During the trial outcome period (minimum of 4 months) we anticipate a mean of 42 babies to be SGA by customised standards only; 42 SGA by population references only; and 126 SGA by both definitions per cluster based on the assumptions above (see distribution on Table 3). However, we have explored the impact on power of fewer babies meeting both SGA definitions, defined as a mean of only 84 SGA babies per cluster in the unlikely extreme scenario where the number of infants in the overlap is reduced by a third. Published reporting using detection during standard antenatal care suggest that, regardless of whether population or customised standards are used to define SGA at birth, around 20% of SGA births are detected antenatally.  For the intervention arm (GAP programme), we anticipate an improvement in our primary outcome from 20% to 33%.

**Table 3: Distribution of SGA and expected detection rates**

|  | | **Number of SGA neonates / 10,000 births** according to pooled estimates of previous studies [1 - 4] | | |
| --- | --- | --- | --- | --- |
|  |  | By population reference only) | By both population reference & customised standards. **(PRIMARY OUTCOME)** | By customised standards only |
| **Total observations with SGA infants** | | 250 | 750 | 250 |
| Detection – standard care arm | % | 20% | 20% | 16% |
|  | N | 50 | 150 | 40 |
| Detection -implementation arm (GAP) | % | 12% | 33% | 33% |
|  | N | 30 | 250 | 83 |

We were unable to identify reports of an intra-cluster correlation coefficient for detection of SGA therefore a coefficient of the most approximate outcome (fetal growth restriction) was used (0.019). A cluster size of 126 SGA infants (by customised standards and population references) and 6 clusters in each arm provides 84% power to demonstrate superiority of GAP at the 5% significance level (two-sided test) for our primary outcome.  In the alternative unlikely scenario of only 84 SGA infants by both definitions per cluster the design provides 79% power. Power calculations were performed using the user-written program *clustersampsi* for Stata.

We have no made no explicit allowance in these calculations for the additional baseline data for each cluster, their inclusion in analysis will increase power.

## Secondary outcomes

We also performed power calculations for two secondary outcomes. This sample size will also provide 91% power to demonstrate a superiority of GAP in detecting SGA defined by customised standards (increase in detection from 19 to 33%) and results in over 90% power to demonstrate non-inferiority of the intervention for the ultrasound detection of SGA by population references (increase from 20 to 28%, considering an example non-inferiority margin of 5%). No formal noninferiority margins are set for any outcomes in this trial, which is largely superiority in nature.

# Analysis Principles

## Analysis population: Intention-to-treat (ITT), per-protocol, or other?

For the primary outcome the primary analysis will be on a modified ITT basis, in which any trusts in the intervention arm that did not contact the GAP provider to initiate training and implement the intervention in the study period due to changes in local strategy are excluded, since such changes are not considered informative concerning how GAP would have performed in the trust. In addition, we shall conduct an ITT analysis including all trusts as randomised, and this provides a conservative sensitivity analysis for our primary analysis in the event that the primary analysis shows a benefit of GAP. A further secondary analysis will be conducted under a per protocol approach restricting analysis of the intervention arm to clusters that complied with GAP. Pre-specified requirements for cluster compliance with GAP comprises: (i) identification of a local multidisciplinary GAP team, (ii) a pre-specified proportion of staff that have completed training and (iii) confirmation that local guidelines and audit are in line with GAP recommendations. A minimum of 75% staff should receive face-to-face training and be e-learning compliant.

We shall approach analysis of the two key secondary outcomes concerning SGA detection by customised and by population centiles in the same way. For other secondary outcomes only the modified ITT analysis will be conducted.

Some outcomes are only applicable for certain subgroups of participants within clusters, notably the SGA detection outcomes which are only defined where the baby is born SGA by a particular definition. Data from women with multiple pregnancies or fetal congenital abnormalities will be excluded from the analysis of all outcomes. Likewise data from any participants who opt out of trial participation will not be compiled and not analysed.

## Significance level of tests

All confidence intervals will be 95% and two-sided. Statistical tests will use a two-sided *p* value of 0.05. There are several secondary clinical outcomes which could form the basis of additional claims of harm or benefit but they are best interpreted holistically rather than focusing on individual statistical tests.

## Baseline comparability

Baseline characteristics of individual participants such as ethnicity, age, and parity will be summarised by randomised group.

## Adjustment for design factors

We will adjust the analyses by a stratification factor in three categories: first group of trusts to be randomised – large trust, first group of trusts to be randomised – small trust, second or third group to be randomised.

## Losses to follow-up: handling missing data

Because outcomes are obtained largely from routine data there can be no loss to follow-up. However there may be missing data for some predictive factors, notably ethnicity. Missing ethnicity is important because this is a factor we wish to adjust for in analysis in case of imbalance and also because it is required in the definition of customised centiles used to define SGA. In some cases it may be possible to assign a baby with missing ethnicity as either SGA or not SGA by customised centiles because birthweight is below the customised SGA threshold for all ethnicities, or above the threshold for all ethnicities, but otherwise missing ethnicity data means that SGA status is missing. Imputation of missing ethnicity will be applied as explained later.

## Cluster level analysis – choice of effect measure and use of baseline data

In trials with a large number of clusters the recommended approach is to apply a regression model to the individual participant data accounting for the clustering for example through random effects. However because of the modest number of clusters in this trial a cluster-summary level analysis is preferred. This will be conducted in two stages to allow for individual factor adjustment. Firstly the cluster summary values will be adjusted for ethnicity, because this is a factor whose distribution may vary considerably between clusters, age and parity. This adjustment will be applied to the summary values for both baseline and main trial period separately. In the second stage an ANCOVA or linear regression analysis will be applied in which the adjusted cluster-summary values for an outcome in the main trial period are compared between intervention and control arms adjusting for the stratification factor and the baseline cluster-summary value for the outcome. The adjusted cluster summary values are calculated on the difference scale and this analysis provides an effect of the intervention on the difference scale (e.g. a change in the proportion with an outcome). This choice of scale was made in preference to alternatives such as a ratio (relative risk) or odds ratio, as it was considered pragmatic and as likely to represent the effect of the intervention as any other choice of scale.

# Analysis details

## Recruitment and follow-up patterns

A CONSORT diagram will be presented to represent recruitment and randomisation of trusts, implementation of the intervention, and numbers of individual women participating and opting out of the trial.

## Participant characteristics

Characteristics of the individual participants in the main trial period will be reported for each of the two trial arms. Summary measures for the baseline characteristics of each arm will be presented as mean and standard deviation for continuous (approximately) normally distributed variables, medians and interquartile ranges for non-normally distributed variables, and frequencies and percentages for categorical variables.

## Analysis Methods

Following the approach described in section 7.2 for baseline characteristics for each outcome we shall report a summary measure by arm for both the baseline and main trial period. Methods of testing for an intervention effect are now described.

### Primary outcome: detail of the two stage cluster-summary method

The approach taken to apply a cluster-summary analysis approach whilst also accounting for individual level predictors follows that explained by Hayes and Moulton [5]. A logistic regression model including ethnicity, age and parity as explanatory factors is fitted to the primary outcome data from all the participants in the main trial period (ignoring clustering by trust). From this model the probability of primary outcome is predicted for all participants and these probabilities can be averaged across participants for each trust separately to give the cluster-summary proportion with the primary outcome expected for that cluster if there is no effect of the intervention. The expected probability can be subtracted from the observed probability to give an adjusted ‘residual’ cluster-summary value. In the same way cluster-summary residuals can be derived separately for the baseline period. The final step is to compare these cluster-summary ‘residual’ values for the main trial period between arms in a linear regression model adjusting for the ‘residual’ values from the baseline and the stratification factor.

Results will be reported as a difference due to the intervention with corresponding 95% confidence intervals (CI) and two-sided p-values. Plots will be presented firstly with a point for each cluster to represent the baseline (horizontal axis) and main trial period (vertical) cluster summary values, and secondly for the baseline and main trial period cluster summary residual values accounting for cluster variation in ethnicity, age and parity.

### Secondary outcomes

The same approach will be taken to test for an intervention effect for secondary outcomes, though for continuous outcomes such as length of stay cluster-summary means will be analysed rather than proportions.

## Sensitivity analyses

As described in section 6.1 different analysis populations will provide sensitivity analyses for the primary and key secondary outcomes. No other sensitivity analyses are planned.

## Subgroup analyses

For the primary outcome we shall explore whether the effect of the intervention on each primary outcome differs by whether the participant presented to the trust before 24 weeks gestation, i.e. received the full ‘exposure’, or not. Our two-stage cluster-summary analysis will be applied for women who present before 24 weeks only to give an intervention effect estimate and 95% CI and then separately applied for women who present later, and the two estimates and 95% CIs can be informally compared (no formal statistical test as the two estimates are not independent and the modest number of clusters precludes a sophisticated analysis of the interaction between full exposure and intervention).

## Adjustment for factors in analysis

We will adjust for age (continuous), parity (first baby or not) and ethnicity in the first stage of our analysis approach (see 7.3.1) and then for the design stratification factor and baseline cluster-summary value in the second stage.

## Regression diagnostics

We shall examine the plots mentioned in 7.3.1 to assess whether a difference (i.e. additive) effect of the intervention fits the data and is therefore good representation of the intervention’s effect on the primary outcome. In the event that the fit is very poor and another representation is clearly better (i.e. greater effect of intervention when a cluster has a higher or lower baseline residual value) then we shall also examine the effect of the intervention on a ratio or odds ratio scale.

## Multiple imputation by chained equations (MICE)

To avoid bias in intervention effect estimates and loss in efficiency, missing predictor values such as ethnicity will be imputed using MICE [6 - 8] under the assumption that missing data values are likely to be missing at random (MAR) which means they are dependent on the values of the observed data, but not dependent on the values of the missing data. Ethnicity is likely to vary strongly between clusters so imputation will be conducted separately by trust. Although ethnicity is a predictor variable it is also used in the definition of SGA by customised centiles so imputing ethnicity also effectively imputes the primary outcome.

Missing ethnicity values will be replaced with simulated values from an imputation model containing age, parity and birthweight. The imputation model will be a multinomial logistic regression model. The number of imputed datasets will be greater than the number of percentage points for which data are missing (e.g. 30 imputations for 30% missing data in ethnicity) considering the cluster with the greatest percentage missing.

Each imputed dataset will be analysed separately and the results combined using Rubin’s rules to produce a single treatment effect estimate and 95% confidence interval. To be clear, the multiple imputation is conducted first and then the two-stage cluster-summary analysis as described in 7.3.1 will be applied to each imputed dataset, and the results combined.

# TABLES AND FIGURES

## Tables

**Table 1: Characteristics of the participants in the main trial period**

| **Groups**  **Variable** | | **Intervention**  **N=**  **%** | **Control**  **N=**  **%** |
| --- | --- | --- | --- |
| Age | Median [IQR], years |  |  |
| Ethnic origin | White |  |  |
|  | Mixed |  |  |
|  | South Asian |  |  |
|  | Black |  |  |
|  | Other |  |  |
| Parity (all live birth) | No children |  |  |
|  | Yes children |  |  |
| Etc |  |  |  |

**Table 2: Effect of intervention on outcome measures**

| Outcome measures | Intervention  % (N) | | Control  % (N) | | Intervention effect  (95% CI) | p-value |
| --- | --- | --- | --- | --- | --- | --- |
|  | Baseline | Main | Baseline | Main |  |  |
| **Primary outcome** |  |  |  |  |  |  |
| Antenatal detection of SGA |  |  |  |  |  |  |
| Modified ITT |  |  |  |  |  |  |
| ITT |  |  |  |  |  |  |
| Per protocol |  |  |  |  |  |  |
| **Secondary outcomes** |  |  |  |  |  |  |
| Antenatal detection of SGA (population) |  |  |  |  |  |  |
| Antenatal detection of SGA (customised) |  |  |  |  |  |  |
| Stillbirth |  |  |  |  |  |  |
| Neonatal death |  |  |  |  |  |  |
| Etc. |  |  |  |  |  |  |

## Figures

**Figure 1: CONSORT diagram**

**Figure 2: Scatter plot of baseline and main trial period antenatal detection of SGA across clusters (with study arm indicated)**

**Figure 3: Scatter plot of baseline and main trial period residual values (ethnicicty adjusted) for antenatal detection of SGA across clusters (with study arm indicated)**

# References

[1] Zhang X, Platt RW, Cnattingius S, Joseph KS, Kramer MS. The use of customised versus population-based birthweight standards in predicting perinatal mortality. BJOG: an international journal of obstetrics and gynaecology. 2007;114(4):474-7.

[2] Clausson B, Gardosi J, Francis A, Cnattingius S. Perinatal outcome in SGA births defined by customised versus population-based birthweight standards. BJOG : an international journal of obstetrics and gynaecology. 2001;108(8):830-4.

[3] Gardosi J, Francis A. Adverse pregnancy outcome and association with small for gestational age birthweight by customized and population-based percentiles. Am J Obstet Gynecol. 2009;201(1):28 e1-8.

[4] Ego A, Subtil D, Grange G, Thiebaugeorges O, Senat MV, Vayssiere C, et al. Customized versus population-based birth weight standards for identifying growth restricted infants: a French multicenter study. Am J Obstet Gynecol. 2006;194(4):1042-9.

[5] Hayes RJ, Moulton LH. Cluster Randomized Trials. Abingdon, UK: Taylor & Francis, 2009.

[6] [White IR](http://www.ncbi.nlm.nih.gov/pubmed?term=White%20IR%5BAuthor%5D&cauthor=true&cauthor_uid=21225900), [Royston P](http://www.ncbi.nlm.nih.gov/pubmed?term=Royston%20P%5BAuthor%5D&cauthor=true&cauthor_uid=21225900), [Wood AM](http://www.ncbi.nlm.nih.gov/pubmed?term=Wood%20AM%5BAuthor%5D&cauthor=true&cauthor_uid=21225900). Multiple imputation using chained equations: Issues and guidance for practice. [Stat Med.](http://www.ncbi.nlm.nih.gov/pubmed/21225900) 2011 Feb 20;30(4):377-99.

[7] Sullivan TR, White IR. Should multiple imputation be the method of choice for handling missing data in randomized trials? SMMR.2017.

[8] Rubin D. Multiple imputation for nonresponse in surveys. New York: Wiley & Sons 1987.
