# Supplementary material for: Evaluation of the Growth Assessment Protocol (GAP) for antenatal detection of small for gestational age: The DESiGN cluster randomised trial
Source: PLoS Med. 2022 Jun 21;19(6):e1004004. doi: 10.1371/journal.pmed.1004004 (PMC9212153; doi:10.1371/journal.pmed.1004004)
Supplement: S2 Appendix — (DOCX) [file pmed.1004004.s003.docx]

# Effect of the Growth Assessment Protocol (GAP) on the detection of small for gestational age: the DESiGN cluster randomised trial.

**S2 Appendix. Additional methodology**

Recommended practice in standard care sites

Compliance with Implementation

Calculation of antenatal detection of SGA

Data management

Statistical analysis - Two-stage cluster-summary statistical approach and one-stage ‘sensitivity’ analysis method

**Recommended practice in standard care sites**

*Fetal growth screening amongst women at low risk of SGA:* There was variation amongst sites on the expected gestation at which to commence fundal height measurements (e.g. 24 weeks, 25 weeks for nulliparous women, 28 weeks for multiparous women). Two sites recommended that fundal heights be plotted onto a population fundal height chart. Two sites recommended use of McDonald’s rule (i.e. that fundal height measured in centimetres should be within 2-3 of gestation measured in weeks). Women with a fundal height outside of the expected range should be referred for a fetal growth scan. Two sites did not have guidance on this. One site recommended assessment of uterine artery Dopplers at the anomaly scan in all women including low risk women and referral for fetal growth scans at 28 and 36 weeks if considered abnormal (mean pulsatility index (PI) >1.25 or total PI>2.5).

*Indications to refer women at low risk of SGA for a fetal growth scan:* Women at two sites were referred for fetal growth scans if the fundal height measurement is 3cm above or below the gestational age measured in weeks. Women at two sites were referred for fetal growth scans if the fundal height measured below the 10th centile (or above the 90th/95th centile) on the population fundal height chart. One site also recommended referral when fundal height measurements were static. Two sites provided no guidance on referral of low risk women for fetal growth scans.

*Defining women at high risk of SGA:* There were wide variation in the risk factors used to identify women at high risk of SGA. One site had no guidance on this. There was agreement that women with hypertension, diabetes and pre-eclampsia were considered at high risk. There were differences in the threshold used for PAPP-A (<0.4 multiples of median), a previous small baby (i.e. <2.5kg), smoking (>10/day only). Additional risk factors considered included young age, underweight BMI, poor obstetric history (e.g. previous pre-eclampsia or placental abruption), alcohol abuse, inflammatory bowel disease, sickle cell disease, mild or moderate pregnancy-induced hypertension. One guideline stratified risk factors into major and minor risk factors, recommending different guidance according to type of risk. Examples of minor risk factors were nulliparity, BMI 30-35, smoking 1-10/day, low fruit intake. Two other guidelines also stratified risk using results of the uterine artery Doppler pulsatility index.

*Fetal growth screening amongst women at high risk of SGA:* Scan protocols for women at high risk of SGA vary widely, but sites offered a minimum interval of 4 weeks for any indication (unless SGA is detected). Sites which offered uterine artery Dopplers used these to increase or reduce the frequency of serial fetal growth scans according to the results. All sites assessed fetal growth using population-based fetal growth reference charts e.g. Hadlock.

**Compliance of implementation**

Fidelity was assessed through degree of adherence of site guidelines to those recommended by the Perinatal Institute, compliance with the training target (>75% of each staff group trained in both face-to-face and e-learning methods) and proportion of notes in which risk stratified was documented correctly according to GAP recommendations [1]. Guidelines were assessed as being poorly (inclusion of less than half the recommended guidance), moderately (partly adherent with inclusion of more than half the recommended guidance) or highly adherent (only occasional deviance from recommended guidance) to GAP. Implementation reach and dose were assessed by calculating the proportion of notes with a GROW chart, number of fundal height measurements plotted for low risk women (median, IQR), proportion of low risk women who had at least the minimum expected number of fundal height plots on the GROW chart (minimum calculated as 3-weekly fundal height measurement plotted from 28 weeks’ gestation until birth), number of growth scans conducted for high risk women, proportion of high-risk women with at least the minimum expected number of growth scans plotted on the GROW chart (minimum calculated as for fundal height measurements) and proportion of low-risk women with a fundal height plot which definitely warranted referral for a fetal growth scan, who received the scan. Results of the overall assessment of implementation strength are summarised in this paper. A detailed analysis will be reported separately.

**Calculation of antenatal detection of SGA**

We obtained electronic maternity records for all births during the study period. The birthweight centile was then calculated for all pregnancies using – zanthro – package in Stata (UK90 birthweight chart). Customised birthweight centile was also calculated for all pregnancies in the study using the GROW bulk calculator provided by the Perinatal Institute. The definition of which birthweight centile (UK90 and/or GROW) was used is described below separately for primary and secondary outcomes.

We obtained electronic ultrasound records for all pregnancies during the study period. We calculated estimated fetal weights (EFW) from biometry where it was not already available in the ultrasound dataset. The EFW centile was then calculated according to the fetal growth chart applicable to each study arm and period of study (Hadlock chart used during pre-randomization in all clusters and in standard care clusters during the outcome period; GROW charts were used for intervention arm clusters during the outcome period). Hadlock centiles were calculated in Stata using the Hadlock formula [2]. GROW centile were calculated using the bulk calculator provided by the Perinatal Institute.

Antenatal detection was calculated as the proportion of babies confirmed as SGA at birth (birthweight centile; denominator) that were identified as SGA antenatally by ultrasound (EFW centile; numerator). For clarity, false positive cases (SGA by estimated fetal weight but not SGA by birthweight) were not included in the numerator, as they were not part of the population of interest (denominator includes only babies confirmed to be SGA at birth).

For the primary outcome the definition of SGA at birth was infants that had birthweight <10th centile by both the UK90 and GROW charts; these infants are the only ones that are targeted by both strategies being assessed in this trial. SGA by ultrasound EFW was defined as EFW<10th centile using the Hadlock chart for babies born in standard care clusters during all the study periods. In the intervention arm clusters, SGA by ultrasound EFW was defined as EFW<10th centile using GROW chart in the intervention arm during the outcome period and as EFW<10th centile using Hadlock charts in the pre-randomisation period. For clarity, SGA by ultrasound was determined solely by the definition being used in the trial arm and time period to assess full performance of each screening strategy/intervention (SGA by both customised and population chart was not applied in EFW centiles). Given the use of SGA at birth by both definitions, false positive rates are not clinically meaningful/interpretable (this would include babies who were actually SGA at birth by either the UK90 charts, or the GROW charts, but not by both). Therefore, the test positive rate was reported for the primary outcome; the test positive rate does not depend on the definition of SGA at birth being used.

For secondary outcomes of SGA at birth by customised centiles, SGA at birth was defined as birthweight <10th centiles using GROW chart for all pregnancies in the trial (both intervention and standard care arm). SGA by ultrasound EFW was defined in the same way as for the primary outcome.

For secondary outcomes of SGA at birth by population centiles, SGA at birth was defined as birthweight <10th centiles using UK90 chart for all pregnancies in the trial (both intervention and standard care arm). SGA by ultrasound EFW was defined in the same way as for the primary outcome.

**Data management**

Data management was performed to harmonise and amalgamate datasets from all clusters. This process has been previously published in detail [3], and will be described briefly here. Data were pseudonymised and linked to a study identification number at each site, before being sent centrally to the trial team. Distribution of each continuous variable was explored, and clinically impossible values were replaced as missing data [3]. For key variables with missing data, multiple imputation with chained equations (MICE) was performed (10 imputations, ‘mi’ functions in Stata) where the assumption could be made that missing data values were missing at random (MAR) [4]. The predictors used for each imputation, and whether imputation was within cluster or trial wide, are listed in detail elsewhere [3]. In summary, a broadly common set of predictors was used for the imputations that included study outcomes and a range of key demographic variables. Imputation was conducted within cluster except for a small number of variables that were data for a given variable was missing for all participants in a cluster. Where trial wide imputation was used, imputed variables were thought unlikely to vary appreciably between clusters. For one cluster, ultrasound measurement data were not available for the baseline period. The proportion of SGA infants detected antenatally (by both definitions) at baseline for this cluster was imputed based on a model fitted to data from the other clusters predicting the number of infants detected based on the number of pregnancies with an ultrasound scan after 24 completed weeks. A sensitivity analysis was also performed where this cluster was excluded from analysis, after request from reviewers.

**Statistical analysis**

**Two-stage cluster-summary statistical approach**

Due to the modest number of clusters, the analysis was performed using a two-stage cluster-summary statistical approach [5]. Firstly, the cluster summary values were adjusted for the ethnicity, age and parity of the individual participants (these are residuals from comparing the observed summary values with those predicted from a model fitted to all participants). This adjustment was applied to the summary values for both baseline and trial outcome period separately. In the second stage, linear regression analysis (ANCOVA) was undertaken in which the adjusted cluster-summary values for an outcome in the trial outcome period were compared between intervention and standard care arms adjusting for the stratification factor, maternal age, ethnicity, parity and the baseline (pre-randomisation) cluster-summary value for that outcome. This analysis provides an effect of the intervention on the difference scale (e.g. a change in the proportion of an outcome). The intervention effect therefore reflects the mean difference between clusters in the intervention and standard care arm and does not directly relate to the reported proportions amongst participants in each trial arm reported in descriptive statistics. All confidence intervals are 95% and two-sided. Statistical tests were interpreted using a two-sided p-value of 0.05.

**One-stage ‘sensitivity’ analysis method**

A reviewer expressed concerns over the application of the two-stage approach to the stillbirth outcome because they felt this may not be an appropriate method for analysis of rare outcomes. Our opinion is that low event rates do not affect two-stage methods more than one-stage approaches, and that the two-stage approach we selected *a-priori* is superior where the number of clusters is modest as in this trial. Nevertheless, we provide the results of the requested post-hoc sensitivity analysis in case any other reader poses the same question regarding the stillbirth finding. We therefore considered one-stage logistic regression analysis based on a simple comparison of intervention and control arms in the trial follow-up period to give an unadjusted odds ratio. The adjusted odds ratio for the intervention effect is derived from analysis of both baseline period and outcome period data together following the constrained baseline approach,[6] and including adjustment for factors as described for the two-stage approach. Our preferred approach was random effects models, which for the constrained baseline model involved a pair of correlated random effects for each clusters one for each period. Such a model was however incompatible with the use of imputed data (the ‘mi estimate’ command) in Stata. The second choice approach was to apply generalised estimating equations with an exchangeable working correlation matrix but this did not converge. The third choice, for which results are presented, is to apply a standard logistic regression method but with robust standard errors to acknowledge the clustering. This analysis was applied under the modified ITT (mITT) approach only.

**References**

1. Clifford S, Giddings S, South M, Williams M, Gardosi J. The Growth Assessment Protocol: a national programme to improve patient safety in maternity care. MIDIRS Midwifery Digest. 2013;23(4):516–23.

2. Hadlock FP, Harrist RB, Martinez-Poyer J. In utero analysis of fetal growth: a sonographic weight standard. Radiology. 1991;181(1):129-33.

3. Relph S, Elstad M, Coker B, Vieira MC, Moitt N, Gutierrez WM, et al. Using electronic patient records to assess the effect of a complex antenatal intervention in a cluster randomised controlled trial-data management experience from the DESiGN Trial team. Trials. 2021;22(1):195.

4. Sterne JA, White IR, Carlin JB, Spratt M, Royston P, Kenward MG, et al. Multiple imputation for missing data in epidemiological and clinical research: potential and pitfalls. BMJ. 2009;338:b2393.

5. Hayes RJ, Moulton LH. Cluster Randomized Trials. Abingdon, UK: Taylor & Francis; 2009.

6. Hooper R, Forbes A, Hemming K, Takeda A, Beresford L. Analysis of cluster randomised trials with an assessment of outcome at baseline. BMJ. 2018;360:k1121.
