# Supplementary material for: Evaluation of the Growth Assessment Protocol (GAP) for antenatal detection of small for gestational age: The DESiGN cluster randomised trial
Source: PLoS Med. 2022 Jun 21;19(6):e1004004. doi: 10.1371/journal.pmed.1004004 (PMC9212153; doi:10.1371/journal.pmed.1004004)
Supplement: S3 Appendix — (DOCX) [file pmed.1004004.s004.docx]

# **Effect of the Growth Assessment Protocol (GAP) on the detection of small for gestational age: the DESiGN cluster randomised trial.**

**S3 Appendix. Supplementary tables and figures.**

Table A. Clinical and sociodemographic characteristics according to treatment allocation – available case analysis and modified intention to treat.

Table B. Clinical and sociodemographic characteristics according to treatment allocation – intention to treat.

Table C. Detailed ethnic characteristics according to treatment allocation – modified intention to treat.

Table D. Detailed ethnic characteristics according to treatment allocation – available case analysis and modified intention to treat.

Table E. Screening performance according to treatment allocation – intention to treat.

Table F. Screening performance according to treatment allocation – available case analysis and modified intention to treat.

Table G. Sensitivity analysis: screening performance according to treatment allocation among women who have had exposure to GAP from 24 weeks gestation (women with ultrasound between 18-24 weeks performed locally) – modified intention to treat.

Table H. Secondary clinical outcomes according to treatment allocation– available case analysis and modified intention to treat.

Table I. Sensitivity analysis: Secondary clinical outcomes according to treatment allocation among women who have had exposure to GAP from 24 weeks gestation (women with ultrasound between 18-24 weeks performed locally) – modified intention to treat.

Figure J. Association between antenatal detection of SGA at baseline and the comparison period across clusters.

# Table A. Clinical and sociodemographic characteristics according to treatment allocation – available case analysis and modified intention to treat.

|  | **Pre-randomisation period** | | **Outcome period** | |
| --- | --- | --- | --- | --- |
|  | **Standard Care**  **(n=29,404)** | **Intervention (GAP) (n=26,546)** | **Standard Care**  **(n=13,810)** | **Intervention (GAP) (n=11,096)** |
| Age at conception (years), median (IQR) | 31.6 (27.5, 35.2) | 31.5 (27.5, 35.1) | 32.0 (28.0, 35.4) | 31.8 (27.9, 35.4) |
| *Missing Age, n* | *18* | *1,424* | *39* | *557* |
| Ethnicity, % (n) |  |  |  |  |
| White | 62.4 (17,682/28,328) | 52.5 (11,228/21,396) | 62.4 (8,348/13,387) | 56.2 (5,627/10,016) |
| Black | 16.6 (4,709/28,328) | 13.7 (2,932/21,396) | 15.4 (2062/13387) | 12.8 (1,281/10,016) |
| Asian | 13.4 (3,787/28,328) | 21.4 (4,588/21,396) | 13.5 (1812/13387) | 20.6 (2,067/10,016) |
| Mixed | 2.2 (611/28,328) | 2.1 (439/21,396) | 2.6 (352/13387) | 1.6 (161/10,016) |
| Other | 5.4 (1,539/28,328) | 10.3 (2,209/21,396) | 6.1 (813/13387) | 8.8 (880/10,016) |
| *Missing Ethnicity, n* | *1,076* | *5,150* | *423* | *1,080* |
| Index of Multiple Deprivation Quintiles, % (n) |  |  |  |  |
| 1 (Least deprived) | 17.5 (5,114/29,252) | 7.6 (1,993/26,341) | 16.6 (2,258/13,623) | 7.5 (821/10,894) |
| 2 | 12.6 (3,678/29,252) | 10.8 (2,849/26,341) | 12.7 (1,729/13,623) | 10.6 (1,153/10,894) |
| 3 | 16.1 (4,707/29,252) | 23.2 (6,114/26,341) | 16.7 (2,268/13,623) | 23.6 (2,572/10,894) |
| 4 | 28.5 (8,328/29,252) | 34.7 (9,143/26,341) | 28.7 (3,903/13,623) | 35.4 (3,856/10,894) |
| 5 (Most deprived) | 25.4 (7,425/29,252) | 23.7 (6,242/26,341) | 25.4 (3,465/13,623) | 22.9 (2,492/10,894) |
| *Missing Index of multiple deprivation, n* | *152* | *205* | *187* | *202* |
| Maternal Height (m), median (IQR) | 1.6 (1.6, 1.7) | 1.6 (1.6, 1.7) | 1.6 (1.6, 1.7) | 1.6 (1.6, 1.7) |
| *Missing Maternal Height, n* | *7,353* | *3,930* | *2,852* | *431* |
| Maternal Weight (kg), median (IQR) | 66.0 (58.8, 76.0) | 64.0 (57.1, 73.9) | 67.2 (59.9, 78.0) | 65.0 (58.0, 75.3) |
| *Missing Maternal Weight, n* | *4,503* | *7,451* | *1,661* | *1,942* |
| Body Mass Index Categories, % (n) |  |  |  |  |
| <18.5 | 2.5 (555/21,892) | 3.1 (596/19,036) | 2.4 (261/10,892) | 2.9 (263/9,140) |
| (18.5-24.9) | 49.3 (10,793/21,892) | 56.5 (10,760/19,036) | 46.2 (5,033/10,892) | 53.3 (4,875/9,140) |
| (25.0-29.9) | 28.8 (6,308/21,892) | 25.8 (4,914/19,036) | 30.5 (3,318/10,892) | 26.9 (2,459/9,140) |
| (30.0-34.9) | 12.7 (2,775/21,892) | 10.0 (1,896/19,036) | 13.9 (1,510/10,892) | 10.8 (991/9,140) |
| (35.0-39.9) | 4.5 (993/21,892) | 3.3 (618/19,036) | 4.8 (524/10,892) | 4.2 (386/9,140) |
| ≥40.0 | 2.1 (468/21,892) | 1.3 (252/19,036) | 2.3 (246/10,892) | 1.8 (166/9,140) |
| *Missing BMI, n* | *7,512* | *7,510* | *2,918* | *1,956* |
| Parity, % (n) |  |  |  |  |
| Nulliparous | 50.6 (13,234/26,133) | 60.0 (13,709/22,839) | 51.2 (6,004/11,727) | 51.8 (5,536/10,693) |
| 1 | 29.0 (7,584/26,133) | 25.7 (5,865/22,839) | 29.8 (3,499/11,727) | 30.2 (3,229/10,693) |
| 2 | 11.8 (3,085/26,133) | 9.1 (2,078/22,839) | 11.1 (1,305/11,727) | 11.0 (1,180/10,693) |
| 3 | 4.7 (1,223/26,133) | 3.1 (710/22,839) | 4.4 (512/11,727) | 4.2 (445/10,693) |
| 4 + | 3.9 (1,007/26,133) | 2.1 (477/22,839) | 3.5 (407/11,727) | 2.8 (303/10,693) |
| *Missing Parity, n* | *3,271* | *3,707* | *2,083* | *403* |

Data are % (n/N); mean (SD); or median (IQR), unless otherwise specified.

Abbreviations: GAP=Growth Assessment Protocol, SLE= Systemic Lupus Erythematous, APS= Antiphospholipid Syndrome,. GDM= Gestational diabetes,. Gest HT=Gestational hypertension.

# Table B. Clinical and sociodemographic characteristics according to treatment allocation – intention to treat.

|  | **Pre-randomisation period** | | | **Outcome period** | | |
| --- | --- | --- | --- | --- | --- | --- |
|  | **Standard Care**  **(n=29,404)** | **Intervention (GAP) (n=36,555)** | | **Standard Care**  **(n=13,810)** | | **Intervention (GAP) (n=15,379)** |
| **Imputed data** |  |  | |  | |  |
| Age at conception (years), median (IQR) | 31.6 (27.5, 35.2) | 31.2 (27.3, 34.9) | | 32.0 (27.9, 35.4) | | 31.3 (27.3, 35.1) |
| Ethnicity, % |  |  | |  | |  |
| White | 62.8 | 50.2 | | 62.7 | | 50.6 |
| Black | 16.2 | 11.8 | | 15.1 | | 10.9 |
| Asian | 13.3 | 24.8 | | 13.5 | | 24.6 |
| Mixed | 2.1 | 1.6 | | 2.6 | | 1.3 |
| Other | 5.5 | 11.6 | | 6.1 | | 12.6 |
| Index of Multiple Deprivation Quintiles, % |  |  | |  | |  |
| 1 (Least deprived) | 17.4 | 8.0 | | 16.5 | | 7.8 |
| 2 | 12.5 | 12.1 | | 12.7 | | 12.1 |
| 3 | 16.1 | 24.8 | | 16.6 | | 25.3 |
| 4 | 28.5 | 35.5 | | 28.7 | | 36.0 |
| 5 (Most deprived) | 25.4 | 19.7 | | 25.5 | | 18.8 |
| Maternal Height (m), median (IQR) | 1.6 (1.6, 1.7) | 1.6 (1.6, 1.7) | | 1.6 (1.6, 1.7) | | 1.6 (1.6, 1.7) |
| Maternal Weight (kg), median (IQR) | 66.0 (58.5, 76.0) | 64.7 (57.3, 74.4) | | 67.0 (59.5, 77.9) | | 65.4 (58.0, 76.0) |
| Body Mass Index Categories, % |  |  | |  | |  |
| <18.5 | 3.9 | 3.9 | | 3.4 | | 3.3 |
| (18.5-24.9) | 50.1 | 52.4 | | 47.2 | | 50.0 |
| (25.0-29.9) | 28.0 | 27.3 | | 29.5 | | 28.2 |
| (30.0-34.9) | 11.9 | 11.1 | | 13.1 | | 12.1 |
| (35.0-39.9) | 4.2 | 3.7 | | 4.6 | | 4.4 |
| ≥40.0 | 2.0 | 1.7 | | 2.2 | | 2.0 |
| Parity, % |  |  | |  | |  |
| Nulliparous | 46.4 | 53.5 | | 47.5 | | 48.4 |
| 1 | 33.8 | 29.1 | | 34.0 | | 32.2 |
| 2 | 11.6 | 10.8 | | 11.0 | | 11.8 |
| 3 | 4.6 | 3.9 | | 4.2 | | 4.4 |
| 4 + | 3.7 | 2.8 | | 3.3 | | 3.1 |
| **Non-imputed data** |  |  | |  | |  |
| Smoking in pregnancy, % (n) | 5.8 (1,646/28,252) | 5.3 (1,645/30,868) | | 5.2 (698/13,466) | | 5.8 (823/14,282) |
| *Missing smoking, n* | *1,152* | *5,687* | | *344* | | *1,097* |
| Pre-existing comorbidities, % (n) |  |  | |  | |  |
| Hypertension | 2.0 (379/19,324) | 1.4 (415/30,010) | | 1.3 (119/9,276) | | 1.4 (187/13,428) |
| *Missing hypertension, n* | *10,080* | *6,545* | | *4,534* | | *1,951* |
| Diabetes | 0.9 (162/18,511) | 2.0 (602/30,170) | | 1.0 (94/9,153) | | 2.6 (343/13,143) |
| *Missing diabetes, n* | *10,893* | *6,385* | | *4,657* | | *2,236* |
| Systemic Lupus Erythematous | 0.18 (35/19,344) | 0.04 (9/25,181) | | 0.17 (16/9,294) | | 0.03 (3/10,610) |
| *Missing SLE, n* | *10,060* | *11,374* | | *4,516* | | *4,769* |
| Antiphospholipid Syndrome | 0.05 (9/19,285) | 0.00 (0/16,653) | | 0.05 (5/9,294) | | 0.00 (0/6,992) |
| *Missing APS, n* | *10,119* | *19,902* | | *4,516* | | *8,387* |
| Pregnancy comorbidities, % (n) |  |  | |  | |  |
| Gestational diabetes | 3.5 (833/23,957) | 5.5 (1,652/30,066) | | 6.3 (713/11,416) | | 7.0 (907/12,972) |
| *Missing GDM, n* | *5,447* | *6,489* | | *2,394* | | *2,407* |
| Gestational hypertension | 1.7 (308/18,506) | 1.6 (402/25,194) | | 1.2 (136/11,418) | | 2.0 (219/10,771) |
| *Missing Gest HT, n* | *10,898* | *11,361* | | *2,392* | | *4,608* |
| Pre-eclampsia | 0.7 (132/18,504) | 1.4 (417/30,129) | | 1.2 (100/8,663) | | 1.7 (234/13,458) |
| *Missing Pre-eclampsia, n* | *10,900* | *6,426* | | *5,147* | | *1,921* |
| Eclampsia | 0.29 (54/18,504) | 0.08 (18/21,351) | | 0.30 (26/8,663) | | 0.09 (8/9,100) |
| *Missing Eclampsia, n* | *10,900* | | *15,204* | | *5,147* | *6,279* |
| Infant sex, male, % (n) | 51.3 (15,086/29,397) | | 51.2 (18,673/36,493) | | 51.1 (7,053/13,798) | 51.0 (7,810/15,304) |
| *Missing Infant sex, n* | *7* | | *62* | | *12* | *75* |

Data are % (n/N); mean (SD); or median (IQR), unless otherwise specified. Where multiple imputation was used numbers are not provided, only percentages.

Abbreviations: GAP=Growth Assessment Protocol, SLE= Systemic Lupus Erythematous, APS= Antiphospholipid Syndrome, GDM= Gestational diabetes, Gest HT=Gestational hypertension.

# Table C. Detailed ethnic characteristics according to treatment allocation – modified intention to treat.

|  | **Pre-randomisation period** | | **Outcome period** | |  |
| --- | --- | --- | --- | --- | --- |
| **Ethnicity, %** | **Standard Care (n=29,404)** | **Intervention (GAP) (n=26,546)** | **Standard Care (n=13,810)** | **Intervention (GAP) (n=11,096)** | |
| Bangladeshi | 1.6 | 1.7 | 1.9 | 1.4 | |
| British European | 51.3 | 49.3 | 45.4 | 49.8 | |
| Caribbean | 3.4 | 2.5 | 3.3 | 2.6 | |
| Central African | 0.4 | 0.1 | 0.4 | 0.1 | |
| Chinese | 1.4 | 1.2 | 1.7 | 1.2 | |
| East African | 1.6 | 2.4 | 1.6 | 2.5 | |
| East European | 5.4 | 4.3 | 7.8 | 4.0 | |
| Indian | 6.5 | 11.6 | 6.1 | 13.3 | |
| Irish European | 1.0 | 1.1 | 1.0 | 1.0 | |
| Middle Eastern | 2.4 | 1.4 | 3.0 | 1.6 | |
| Mixed African-European | 0.7 | 0.4 | 0.8 | 0.3 | |
| Mixed Asian-European | 0.7 | 0.7 | 0.8 | 0.6 | |
| Mixed Caribbean-European | 0.8 | 0.9 | 1.0 | 0.7 | |
| North African | 0.6 | 0.5 | 0.7 | 0.4 | |
| North European | 0.3 | 0.1 | 0.3 | 0.1 | |
| Other | 4.7 | 8.6 | 5.2 | 7.9 | |
| Other Far East | 0.7 | 1.0 | 0.6 | 1.0 | |
| Pakistani | 2.3 | 3.5 | 2.4 | 3.1 | |
| South African Black | 0.3 | 0.2 | 0.3 | 0.1 | |
| South East Asia | 0.7 | 0.4 | 0.8 | 0.3 | |
| South European | 1.9 | 0.5 | 4.2 | 0.5 | |
| West African | 10.6 | 7.6 | 9.5 | 7.4 | |
| West European | 1.1 | 0.3 | 1.4 | 0.3 | |

Data are % (n/N), unless otherwise specified. Where multiple imputation was used numbers are not provided, only percentages.

# Table D. Detailed ethnic characteristics according to treatment allocation – available case analysis and modified intention to treat.

|  | **Pre-randomisation period** | | **Outcome period** | |
| --- | --- | --- | --- | --- |
| **Ethnicity, % (n)** | **Standard Care (n=29,404)** | **Intervention (GAP) (n=26,546)** | **Standard Care (n=13,810)** | **Intervention (GAP) (n=11,096)** |
| Bangladeshi | 1.7 (468/28,328) | 1.9 (410/21,396) | 1.9 (254/13,387) | 1.5 (148/10,016) |
| British European | 50.6 (14,337/28,328) | 45.0 (9,635/21,396) | 44.6 (5,967/13,387) | 49.3 (4,938/10,016) |
| Caribbean | 3.4 (972/28,328) | 2.6 (553/21,396) | 3.4 (456/13,387) | 2.6 (257/10,016) |
| Central African | 0.4 (101/28,328) | 0.1 (19/21,396) | 0.4 (56/13,387) | 0.1 (6/10,016) |
| Chinese | 1.4 (400/28,328) | 1.3 (272/21,396) | 1.6 (219/13,387) | 1.1 (111/10,016) |
| East African | 1.6 (461/28,328) | 2.5 (543/21,396) | 1.6 (220/13,387) | 2.5 (251/10,016) |
| East European | 5.5 (1,556/28,328) | 4.9 (1,040/21,396) | 8.0 (1,073/13,387) | 4.4 (444/10,016) |
| Indian | 6.4 (1,820/28,328) | 12.8 (2,735/21,396) | 6.0 (800/13,387) | 13.5 (1,352/10,016) |
| Irish European | 0.9 (265/28,328) | 1.1 (238/21,396) | 1.0 (137/13,387) | 0.9 (92/10,016) |
| Middle Eastern | 2.5 (694/28,328) | 1.7 (352/21,396) | 3.1 (420/13,387) | 1.7 (171/10,016) |
| Mixed African-European | 0.7 (188/28,328) | 0.4 (79/21,396) | 0.8 (112/13,387) | 0.3 (25/10,016) |
| Mixed Asian-European | 0.7 (192/28,328) | 0.8 (162/21,396) | 0.8 (106/13,387) | 0.6 (63/10,016) |
| Mixed Caribbean-European | 0.8 (231/28,328) | 0.9 (198/21,396) | 1.0 (134/13,387) | 0.7 (73/10,016) |
| North African | 0.6 (159/28,328) | 0.6 (121/21,396) | 0.7 (90/13,387) | 0.4 (41/10,016) |
| North European | 0.3 (74/28,328) | 0.0 (9/21,396) | 0.3 (43/13,387) | 0.1 (8/10,016) |
| Other | 4.6 (1,288/28,328) | 8.7 (1,871/21,396) | 5.1 (680/13,387) | 7.3 (734/10,016) |
| Other Far East | 0.8 (217/28,328) | 1.2 (246/21,396) | 0.7 (88/13,387) | 1.0 (103/10,016) |
| Pakistani | 2.4 (668/28,328) | 3.8 (822/21,396) | 2.4 (323/13,387) | 3.2 (318/10,016) |
| South African Black | 0.3 (90/28,328) | 0.2 (32/21,396) | 0.3 (34/13,387) | 0.1 (9/10,016) |
| South East Asia | 0.7 (199/28,328) | 0.5 (100/21,396) | 0.9 (114/13,387) | 0.3 (33/10,016) |
| South European | 1.9 (541/28,328) | 0.5 (109/21,396) | 4.3 (581/13,387) | 0.5 (52/10,016) |
| West African | 10.9 (3,080/28,328) | 8.3 (1,784/21,396) | 9.7 (1,294/13,387) | 7.6 (758/10,016) |
| West European | 1.2 (327/28,328) | 0.3 (66/21,396) | 1.4 (186/13,387) | 0.3 (29/10,016) |
| *Unclassified (missing), n* | *1,076* | *5,150* | *423* | *1,080* |

Data are % (n/N), unless otherwise specified.

# Table E. Screening performance according to treatment allocation – intention to treat.

|  | **Pre-randomisation period** | | **Outcome period** | | **Intervention effect size - unadjusted (95%CI)** | **Intervention effect size – adjusted* (95%CI)** | **p-value** |
| --- | --- | --- | --- | --- | --- | --- | --- |
|  | **Standard Care**  **(n= 29,404)** | **Intervention (GAP) (n=36,555)** | **Standard Care (n=13,810)** | **Intervention (GAP) (n=15,739)** |  |  |  |
| **Primary outcome (SGA by customised and population centiles)** |  |  |  |  |  |  |  |
| Proportion of SGA (birthweight), % | 7.2 | 7.8 | 7.2 | 8.1 | - | - |  |
| Antenatal detection of SGA, % | 19.1 | 23.6 | 27.7 | 22.1 | -4.0 (-14.8, 6.8) | -3.5  (-14.0, 7.0) | 0.52 |
| Test positive rate, % | 2.4 | 3.2 | 3.4 | 3.1 | 0.1  (-1.6, 1.9) | -0.3  (-1.7, 1.2) | 0.73 |
| **Secondary outcomes** |  |  |  |  |  |  |  |
| **SGA by customised centiles** |  |  |  |  |  |  |  |
| Proportion of SGA (birthweight), % | 11.2 | 11.0 | 11.6 | 12.3 | - | - |  |
| Antenatal detection of SGA, % | 14.9 | 19.1 | 21.5 | 19.1 | -1.6 (-10.1, 6.9) | -1.8  (-10.3, 6.8) | 0.68 |
| Specificity †, % | 99.1 | 98.8 | 99.0 | 99.1 | - | - |  |
| Positive predictive value‡, % | 68.9 | 66.6 | 73.3 | 73.8 | - | - |  |
| Negative predictive value‡, % | 90.2 | 90.6 | 90.6 | 89.3 | - | - |  |
| False positive rate †, % | 0.9 | 1.2 | 1.0 | 1.0 | 0.2  (-0.6, 0.9) | 0.0 (-0.7, 0.6) | 0.91 |
| False negative rate, % | 85.1 | 80.9 | 78.5 | 80.9 |  |  |  |
| **SGA by population centiles** |  |  |  |  |  |  |  |
| Proportion of SGA (birthweight), % | 8.6 | 10.1 | 8.5 | 10.2 | - | - |  |
| Antenatal detection of SGA, % | 17.1 | 20.5 | 25.0 | 17.9 | -5.1 (-15.2, 4.9) | -4.0 (-13.0, 4.9) | 0.38 |
| Specificity †, % | 99.0 | 98.9 | 98.6 | 98.6 | - | - |  |
| Positive predictive value‡, % | 60.9 | 66.5 | 62.5 | 58.8 | - | - |  |
| Negative predictive value‡, % | 92.7 | 91.6 | 93.4 | 91.4 | - | - |  |
| False positive rate †, % | 1.0 | 1.2 | 1.4 | 1.5 | 0.4  (-0.8, 1.6) | 0.2  (-0.8, 1.1) | 0.74 |
| False negative rate, % | 82.9 | 79.5 | 75.0 | 82.1 | - | - |  |

Data are % (n/N). Where multiple imputation was used numbers are not provided, only percentages. Effect size provided are differences (intervention minus standard care arm) for the outcome period. with 95% confidence intervals and p-values are derived from linear regression where the dependent variable for each outcome was the adjusted cluster summary; p-values are reported only for the adjusted analysis.

Abbreviations: GAP=Growth Assessment Protocol, SGA=small for gestational age infant.

* adjusted for baseline, age, ethnicity, parity, stratification factor. †Excludes one cluster. ‡Pre-randomisation values excludes two clusters but outcome period excludes only one cluster.

# Table F. Screening performance according to treatment allocation – available case analysis and modified intention to treat.

|  | **Pre-randomisation period** | | **Outcome period** | | **Intervention effect size - unadjusted (95%CI)** | **Intervention effect size – adjusted* (95%CI)** | **p-value** |
| --- | --- | --- | --- | --- | --- | --- | --- |
|  | **Standard Care**  **(n= 29,404)** | **Intervention (GAP) (n=26,546)** | **Standard Care (n=13,810)** | **Intervention (GAP) (n=11,096)** |  |  |  |
| **Primary outcome (SGA by customised and population centiles)** |  |  |  |  |  |  |  |
| Proportion of SGA (birthweight), % (n) | 7.3 (2,134/29,298) | 7.7 (1,932/25,008) | 7.3  (995/13,597) | 7.7 (807/10,473) | - | - |  |
| Antenatal detection of SGA, % (n) † | 19.0 (406/2,134) | 26.2 (507/1,932) | 27.8  (277/995) | 28.0  (226/807) | 2.6  (-6.7, 11.9) | 0.1  (-13.4, 13.5) | 0.99 |
| Test positive rate, % | 2.4 | 3.3 | 3.4 | 3.8 | 0.8  (-0.8, 2.4) | -0.2  (-1.8, 1.4) | 0.76 |
| **Secondary outcomes**‡ | | | | | | | |
| **SGA by customised centiles** |  |  |  |  |  |  |  |
| Proportion of SGA (birthweight) , % (n) | 11.7 (3,422/29,305) | 11.7 (2,021/17,281) | 12.2 (1,652/13,597) | 13.1 (975/7,461) | - | - |  |
| Antenatal detection of SGA, % (n) | 14.6 (500/3,422) | 20.8 (420/2,021) | 21.2  (350/1,652) | 21.7  (212/975) | 1.0 (-5.2, 7.1) | -0.2  (-12.5, 12.1) | 0.97 |
| Specificity, % | 99.2 | 98.8 | 99.0 | 99.0 | - | - |  |
| Positive predictive value, % | 70.8 | 69.1 | 75.0 | 76.3 | - | - |  |
| Negative predictive value, % | 89.8 | 90.4 | 90.1 | 89.4 | - | - |  |
| False positive rate, % | 0.8 | 1.2 | 1.0 | 1.0 | 0.1  (-0.6, 0.7) | -0.1  (-0.9, 0.7) | 0.72 |
| False negative rate, % | 85.4 | 79.2 | 78.8 | 78.3 | - | - |  |
| **SGA by population centiles** |  |  |  |  |  |  |  |
| Proportion of SGA (birthweight), % (n) | 8.6 (2,510/29,302) | 10.0  (1,717/17,242) | 8.5 (1,158/13,597) | 10.0  (741/7,426) | - | - |  |
| Antenatal detection of SGA, % (n) | 17.1 (430/2,510) | 22.8 (391/1,717) | 25.3  (293/1,158) | 22.0  (163/741) | -3.6  (-11.2, 4.0) | -3.2  (-17.6, 11.2) | 0.57 |
| Specificity, % | 99.0 | 98.6 | 98.6 | 98.3 | - | - |  |
| Positive predictive value, % | 61.0 | 64.4 | 62.7 | 58.8 | - | - |  |
| Negative predictive value, % | 92.7 | 92.0 | 93.4 | 91.9 | - | - |  |
| False positive rate, % | 1.0 | 1.4 | 1.4 | 1.7 | 0.4  (-0.4, 1.0) | 0.2  (-0.8, 1.2) | 0.58 |
| False negative rate, % | 82.9 | 77.2 | 74.7 | 78.0 | - | - |  |

Data are % (n/N). Effect size provided are differences (intervention minus standard care arm) for the outcome period. with 95% confidence intervals and p-values are derived from linear regression where the dependent variable for each outcome was the adjusted cluster summary; p-values are reported only for the adjusted analysis.

Abbreviations: GAP=Growth Assessment Protocol, SGA=small for gestational age infant.

* adjusted for baseline, age, ethnicity, parity, stratification factor. †Excludes 1 cluster. ‡Excludes 2 clusters.

# Table G. Sensitivity analysis: screening performance according to treatment allocation among women who have had exposure to GAP from 24 weeks gestation (women with ultrasound between 18-24 weeks performed locally) – modified intention to treat.

|  | **Pre-randomisation period** | | | **Outcome period** | | **Intervention effect size - unadjusted (95%CI)** | **Intervention effect size – adjusted* (95%CI)** | **p-value** |
| --- | --- | --- | --- | --- | --- | --- | --- | --- |
|  | **Standard Care (n=19,473)** | | **Intervention (GAP) (n=21,807)** | **Standard Care (n=12,212)** | **Intervention (GAP) (n=9,344)** |  |  |  |
| **Primary outcome** |  |  | |  |  |  |  |  |
| Proportion of neonates who were SGA by customised and population centiles, % | 6.8 | 7.5 | | 7.0 | 7.4 | - | - |  |
| Antenatal detection of SGA at birth by customised and population centiles, % | 27.9 | 27.5 | | 29.0 | 28.1 | 1.1 (-7.4, 9.6) | 2.1  (-6.4, 10.5) | 0.63 |
| Test positive rate, % | 3.4 | 3.5 | | 3.5 | 3.8 | 0.7 (-0.6, 2.0) | 0.0 (-1.0, 1.0) | 0.97 |
| **Secondary outcomes**† |  |  | |  |  |  |  |  |
| Proportion of neonates who were SGA by customised centiles, % | 11.2 | 11.2 | | 11.6 | 12.5 |  |  |  |
| Antenatal detection of SGA at birth by customised centiles (<10th centile), % | 14.9 | 19.6 | | 21.5 | 20.3 | -0.5 (-6.6, 5.6) | 0.6 (-4.9, 6.1) | 0.83 |
| Specificity, % | 99.2 | 98.8 | | 99.0 | 99.0 | - | - |  |
| Positive predictive value, % | 68.9 | 67.1 | | 73.3 | 74.4 | - | - |  |
| Negative predictive value, % | 90.2 | 90.7 | | 90.6 | 89.7 | - | - |  |
| False positive rate, % | 0.9 | 1.2 | | 1.0 | 1.0 | - | - |  |
| False negative rate, % | 85.2 | 80.4 | | 78.6 | 79.7 | - | - |  |
| Proportion of neonates who were SGA by population centiles, % | 8.6 | 10.1 | | 8.5 | 9.9 | - | - |  |
| Antenatal detection of SGA at birth by population centiles (<10th centile), % | 17.1 | 20.8 | | 25.0 | 19.7 | -5.1 (-12.3, 2.0) | -1.9 (-7.6, 3.7) | 0.50 |
| Specificity, % | 99.0 | 98.7 | | 98.6 | 98.4 | - | - |  |
| Positive predictive value, % | 60.9 | 64.2 | | 62.5 | 57.7 | - | - |  |
| Negative predictive value, % | 92.7 | 91.8 | | 93.4 | 91.7 | - | - |  |
| False positive rate, % | 1.0 | 1.3 | | 1.4 | 1.6 | - | - |  |
| False negative rate, % | 82.9 | 79.2 | | 75.0 | 80.4 | - | - |  |

Data are % (n/N), unless otherwise specified. Where multiple imputation was used numbers are not provided, only percentages. Effect size provided are differences (intervention minus standard care arm) for the outcome period. with 95% confidence intervals and p-values are derived from linear regression where the dependent variable for each outcome was the adjusted cluster summary; p-values are reported only for the adjusted analysis.

Abbreviations: GAP=Growth Assessment Protocol, SGA=small for gestational age infant.

* adjusted for baseline, age, ethnicity, parity, stratification factor. †Excludes 2 clusters.

# Table H. Secondary clinical outcomes according to treatment allocation– available case analysis and modified intention to treat.

|  | **Pre-randomisation period** | | **Outcome period** | | **Intervention effect size - unadjusted (95%CI)** | **Intervention effect size – adjusted* (95%CI)** | **p-value** |
| --- | --- | --- | --- | --- | --- | --- | --- |
|  | **Standard Care (n=29,404)** | **Intervention (GAP) (n=26,546)** | **Standard Care (n=13,810)** | **Intervention (GAP) (n=11,096)** |  |  |  |
| **Maternal outcomes** |  |  |  |  |  |  |  |
| Induction of Labour, % (n) | 25.1 (7,337/29,253) | 25.2 (6,145/24,360) | 27.0 (3,700/13,728) | 29.4 (3,185/10,844) | 2.7  (-5.2, 10.6) | -0.8 (-8.8, 7.1) | 0.81 |
| Mode of birth, % (n) |  |  |  |  |  |  |  |
| *Spontaneous vaginal delivery* | *58.1 (17,053/29,355)* | *58.4 (14,953/25,617)* | *54.5 (7,510/13,777)* | *54.1 (5,848/10,806)* | *2.1  (-5.6, 9.9)* | *-4.6  (-22.8, 13.6)* | *0.56* |
| *Operative vaginal delivery* | *13.7 (4,013/29,355)* | *15.3 (3,919/25,617)* | *14.1 (1,945/13,777)* | *14.3 (1,545/10,806)* | *0.1  (-3.7, 3.9)* | *-2.7  (-7.9, 2.5)* | *0.25* |
| *Elective caesarean section* | *12.3 (3,611/29,355)* | *12.4 (3,168/25,617)* | *13.9 (1,912/13,777)* | *14.6 (1,577/10,806)* | *-1.2  (-6.9, 4.5)* | *-2.6  (-8.2, 2.9)* | *0.29* |
| *Emergency caesarean section* | *15.6 (4,578/29,355)* | *13.7 (3,508/25,617)* | *17.2 (2,366/13,777)* | *16.7 (1,801/10,806)* | *-1.1  (-5.4, 3.3)* | *-1.8  (-5.8, 2.2)* | *0.32* |
| Post-partum haemorrhage (>1500mls), % (n) | 2.7  (779/29,204) | 2.3  (610/26,106) | 2.7  (375/13,728) | 2.5  (271/10,808) | -0.4  (-1.3, 0.5) | -0.5  (-1.2, 0.3) | 0.17 |
| 3rd/4th degree tears, % (n) | 2.2  (657/29,404) | 2.4  (629/26,546) | 1.9  (262/13,810) | 1.8  (197/11,096) | 0.0  (-0.8, 0.7) | -0.5  (-1.2, 0.2) | 0.12 |
| Epidural, % (n) | 36.5 (8,909/24,399) | 27.9 (7,384/26,475) | 36.4 (4,213/11,568) | 28.2 (3,127/11,076) | -13.0  (-33.7, 7.7) | -5.4  (-15.5, 4.8) | 0.25 |
| Episiotomy, % (n) | 17.7 (4,331/24,415) | 23.1 (4,707/20,381) | 17.6 (2,160/12,307) | 21.8 (1,763/8,100) | 16.4  (-10.1, 43.0) | -2.1  (-9.4, 5.2) | 0.50 |
| **Neonatal outcomes** |  |  |  |  |  |  |  |
| Gestational age at birth (weeks), mean (SD) | 39.5 (2.0) | 39.5 (1.9) | 39.4 (1.9) | 39.4 (2.0) | -0.1  (-0.2, 0.1) | 0.0  (-0.1, 0.1) | 0.92 |
| *Preterm birth (<37 weeks), % (n)* | *5.6 (1,654/29,340)* | *6.0  (1,506/25,121)* | *6.1  (825/13,624)* | *6.4  (670/10,539)* | *0.3  (-1.3, 1.8)* | *-0.6  (-2.3, 1.2)* | *0.46* |
| Birthweight (g), mean (SD) | 3,348.3 (557.6) | 3,326.6 (554.9) | 3,326.4 (551.5) | 3,299.3 (563.1) | -23.4  (-96.0, 49.2) | -11.1  (-35.7, 13.5) | 0.31 |
| ***Condition at birth*** |  |  |  |  |  |  |  |
| Apgar score < 7 at 5 minutes, % (n) | 2.0 (575/29,404) | 1.7  (460/26,546) | 2.1  (290/13,810) | 1.6  (173/11,096) | -0.5  (-1.3, 0.3) | -0.6  (-1.5, 0.4) | 0.18 |
| Arterial cord pH < 7.1, % (n) | 2.3  (555/24,674) | 2.8  (730/26,546) | 2.0  (236/11,655) | 2.9  (317/11,096) | 0.7  (-1.0, 2.4) | 0.1  (-1.1, 1.3) | 0.78 |
| Respiratory support at birth, % (n) | 4.4 (1,084/24,674) | 6.3 (1,664/26,546) | 4.1  (481/11,655) | 4.8  (527/11,096) | 1.2  (-3.5, 5.8) | -1.8  (-4.9, 1.3) | 0.20 |
| ***Neonatal admissions*** |  |  |  |  |  |  |  |
| Neonatal unit admission (inc HDU & SCBU), n (%) | 14.9 (3,549/23,836) | 8.1 (2,146/26,546) | 16.2 (1,785/11,024) | 7.4  (824/11,096) | -8.3  (-27.5, 10.8) | -4.1  (-11.5, 3.4) | 0.22 |
| ***Major neonatal morbidity*** |  |  |  |  |  |  |  |
| Any major neonatal retinopathy above, % (n) | 4.5 (1,331/29,404) | 6.2 (1,639/26,546) | 5.5  (754/13,810) | 4.7  (526/11,096) | -1.5  (-4.9, 1.8) | -1.2  (-4.2, 1.7) | 0.35 |
| *Any neonatal brain injury (HIE + IVH), % (n)* | *0.44  (128/29,404)* | *0.44  (118/26,546)* | *0.41  (56/13,810)* | *0.34  (38/11,096)* |  |  |  |
| *Supplementary O_2_ >28 days, % (n)* | *0.16  (47/29,404)* | *0.16 (43/26,546)* | *0.09  (13/13,810)* | *0.15  (17/11,096)* |  |  |  |
| *Necrotising enterocolitis, % (n)* | *0.18  (52/29,404)* | *0.15  (33/21,596)* | *0.12  (17/13,810)* | *0.08  (7/8,882)* |  |  |  |
| *Sepsis, % (n)* | *4.50 (1,323/29,404)* | *6.13 (1,626/26,546)* | *5.37  (742/13,810)* | *4.60  (510/11,096)* |  |  |  |
| *Neonatal retinopathy, % (n)* | *0.11  (30/26,521)* | *0.12  (25/21,596)* | *0.17  (21/12,481)* | *0.06  (5/8,882)* |  |  |  |
| ***Minor Neonatal morbidity*** |  |  |  |  |  |  |  |
| Any minor neonatal morbidity, % (n) | 2.8  (835/29,404) | 4.5 (1,190/26,546) | 2.6  (352/13,810) | 3.0  (332/11,096) | 0.5  (-1.4, 2.4) | -0.2  (-2.1, 1.7) | 0.84 |
| *Hypothermia, % (n)* | *0.14  (42/29,404)* | *0.41 (109/26,546)* | *0.17  (23/13,810)* | *0.14  (16/11,096)* |  |  |  |
| *Hypoglycaemia, % (n)* | *1.43  (421/29,404)* | *1.72  (456/26,546)* | *1.19  (164/13,810)* | *0.86  (95/11,096)* |  |  |  |
| *Nasogastric feeding, % (n)* | *2.37  (564/23,836)* | *3.62  (962/26,546)* | *1.98  (218/11,024)* | *2.62  (291/11,096)* |  |  |  |
| ***Perinatal loss*** |  |  |  |  |  |  |  |
| Stillbirth, % (n) | 0.30  (88/29,403) | 0.40  (105/26,510) | 0.36  (50/13,780) | 0.31  (34/11,068) | -0.05  (-0.21, 0.11) | -0.06  (-0.20, 0.09) | 0.40 |
| Neonatal death, % (n) | 0.07  (20/29,404) | 0.13  (34/26,546) | 0.04  (6/13,810) | 0.07  (8/11,096) | 0.01  (-0.08, 0.10) | -0.02  (-0.10, 0.07) | 0.64 |
| Perinatal mortality, % (n) | 0.37 (108/29,403) | 0.49 (129/26,510) | 0.41  (56/13,780) | 0.37  (41/11,068) | -0.05  (-0.27, 0.17) | -0.08  (-0.28, 0.13) | 0.40 |

Data are % (n/N) or mean (SD). Effect size provided are differences (intervention minus standard care arm) for the outcome period. with 95% confidence intervals and p-values are derived from linear regression where the dependent variable for each outcome was the adjusted cluster summary; p-values are reported only for the adjusted analysis.

Abbreviations: GAP= Growth Assessment Protocol, HDU=high dependence unit, SCBU=special care baby unit, HIE=hypoxic ischemic injury, IVH=intraventricular hemorrhage, O_2_=oxygen.

* adjusted for baseline, age, ethnicity, parity, stratification factor.

# Table I. Sensitivity analysis: Secondary clinical outcomes according to treatment allocation among women who have had exposure to GAP from 24 weeks gestation (women with ultrasound between 18-24 weeks performed locally) – modified intention to treat.

|  | **Pre-randomisation period** | | **Outcome comparison period** | | **Intervention effect size - unadjusted (95%CI)** | **Intervention effect size – adjusted* (95%CI)** | **p-value** |
| --- | --- | --- | --- | --- | --- | --- | --- |
|  | **Standard Care (n=19,473)** | **Intervention (GAP) (n=21,807)** | **Standard Care (n=12,212)** | **Intervention (GAP) (n=9,344)** |  |  |  |
| **Maternal outcomes** |  |  |  |  |  |  |  |
| Induction of Labour, % | 25.5 | 26.4 | 27.1 | 29.4 | 2.8 (-4.4, 9.9) | 1.3 (-0.5, 3.2) | 0.15 |
| Mode of birth, % |  |  |  |  |  |  |  |
| *Spontaneous vaginal delivery* | *58.8* | *58.8* | *54.1* | *53.8* | *1.8 (-4.4, 8.1)* | *1.3 (-1.2, 3.8)* | *0.31* |
| *Operative vaginal delivery* | *13.6* | *15.1* | *14.2* | *14.7* | *0.3 (-3.1, 3.8)* | *-0.1 (-1.8, 1.7)* | *0.94* |
| *Elective caesarean section* | *12.9* | *12.5* | *14.4* | *14.6* | *-1.3 (-5.8, 3.2)* | *-1.4 (-2.9, 0.1)* | *0.06* |
| *Emergency caesarean section* | *14.5* | *13.4* | *17.1* | *16.7* | *-0.9 (-4.6, 2.9)* | *-0.1 (-2.3, 2.2)* | *0.97* |
| Post-partum haemorrhage (>1500mls), % | 2.6 | 2.4 | 2.7 | 2.5 | -0.3 (-1.2, 0.5) | 0.0 (-0.4, 0.4) | 0.95 |
| 3rd/4th degree tears, % | 2.3 | 2.5 | 1.9 | 2.0 | 0.2 (-0.7, 1.0) | 0.0 (-0.5, 0.5) | 0.90 |
| Epidural, % | 37.1 | 27.5 | 36.5 | 28.0 | -12.9 (-33.6, 7.9) | 6.2 (-1.5, 14.0) | 0.12 |
| Episiotomy, % | 16.3 | 22.4 | 17.6 | 22.4 | 16.9 (-9.6, 43.3) | -2.2 (-6.7, 2.3) | 0.33 |
| **Neonatal outcomes** |  |  |  |  |  |  |  |
| Gestational age at birth (weeks), mean (SD)** | 39.6 (1.7) | 39.5 (1.9) | 39.5 (1.8) | 39.4 (1.9) | -0.1 (-0.2, 0.1) | 0.0 (-0.1, 0.1) | 0.74 |
| *Preterm birth (<37 weeks), %* | *4.9* | *5.5* | *5.5* | *5.7* | *0.2 (-1.2, 1.6)* | *-0.2 (-0.7, 0.3)* | *0.45* |
| Birthweight (g), mean (SD) ** | 3,376.0 (533.0) | 3,338.3 (547.2) | 3,341.5 (531.3) | 3,309.8 (551.8) | -27.0 (-82.5, 28.5) | -12.0 (-26.4, 2.4) | 0.10 |
| ***Condition at birth*** |  |  |  |  |  |  |  |
| Apgar score < 7 at 5 minutes, % | 1.9 | 1.6 | 2.0 | 1.5 | -0.4 (-1.2, 0.4) | -0.1 (-0.5, 0.3) | 0.60 |
| Arterial cord pH < 7.1, % | 1.8 | 2.7 | 2.0 | 2.8 | 0.7 (-1.1, 2.5) | 0.2 (-0.6, 0.9) | 0.71 |
| Respiratory support at birth, % | 3.5 | 6.2 | 3.9 | 4.6 | 1.1 (-3.3, 5.5) | -1.3 (-3.2, 0.5) | 0.15 |
| ***Neonatal admissions*** |  |  |  |  |  |  |  |
| Neonatal unit admission (inc HDU & SCBU), % | 13.7 | 7.9 | 15.3 | 6.9 | -8.7 (-28.0, 10.7) | 0.3 (-0.9, 1.6) | 0.61 |
| ***Major neonatal morbidity*** |  |  |  |  |  |  |  |
| Any major neonatal retinopathy above, % | 5.0 | 6.1 | 5.1 | 4.5 | -1.5 (-4.9, 2.0) | -1.2 (-3.3, 0.9) | 0.25 |
| *Any neonatal brain injury (HIE + IVH), %* | *0.36* | *0.36* | *0.30* | *0.27* | - | - | - |
| *Supplementary O2 >28 days, %* | *0.07* | *0.12* | *0.07* | *0.11* | - | - | - |
| *NEC, %* | *0.12* | *0.16* | *0.11* | *0.05* | - | - | - |
| *Sepsis, %* | *4.97* | *6.07* | *5.01* | *4.38* | - | - | - |
| *Neonatal retinopathy, %* | *0.07* | *0.13* | *0.10* | *0.03* | - | - | - |
| ***Minor neonatal morbidity*** |  |  |  |  |  |  |  |
| Any minor neonatal morbidity, % | 2.9 | 4.3 | 2.2 | 2.7 | 0.5 (-1.2, 2.1) | 0.0 (-1.4, 1.4) | 0.95 |
| *Hypothermia, %* | *0.15* | *0.40* | *0.16* | *0.15* | - | - | - |
| *Hypoglycaemia, %* | *1.57* | *1.68* | *1.14* | *0.83* | - | - | - |
| *NG feeding, %* | *1.99* | *3.45* | *1.59* | *2.38* | - | - | - |
| ***Perinatal loss*** |  |  |  |  |  |  |  |
| Stillbirth, % | 0.24 | 0.31 | 0.29 | 0.29 | 0.01 (-0.14, 0.16) | 0.01 (-0.08, 0.10) | 0.80 |
| Neonatal death, % | 0.06 | 0.11 | 0.02 | 0.05 | 0.01 (-0.07, 0.09) | 0.00 (-0.06, 0.06) | 0.92 |
| Perinatal mortality, % | 0.29 | 0.37 | 0.32 | 0.34 | 0.02 (-0.13, 0.17) | 0.01 (-0.05, 0.08) | 0.69 |

Data are % (n/N) or mean (SD). Where multiple imputation was used numbers are not provided, only percentages. Effect size provided are differences (intervention minus standard care arm) for the outcome period. with 95% confidence intervals and p-values are derived from linear regression where the dependent variable for each outcome was the adjusted cluster summary; p-values are reported only for the adjusted analysis.

Abbreviations: GAP= Growth Assessment Protocol, HDU=high dependence unit, SCBU=special care baby unit, HIE=hypoxic ischemic injury, IVH=intraventricular hemorrhage, O_2_=oxygen.

* adjusted for baseline, age, ethnicity, parity, stratification factor.

# Figure J. Association between antenatal detection of SGA at pre-randomisation and comparison periods across clusters


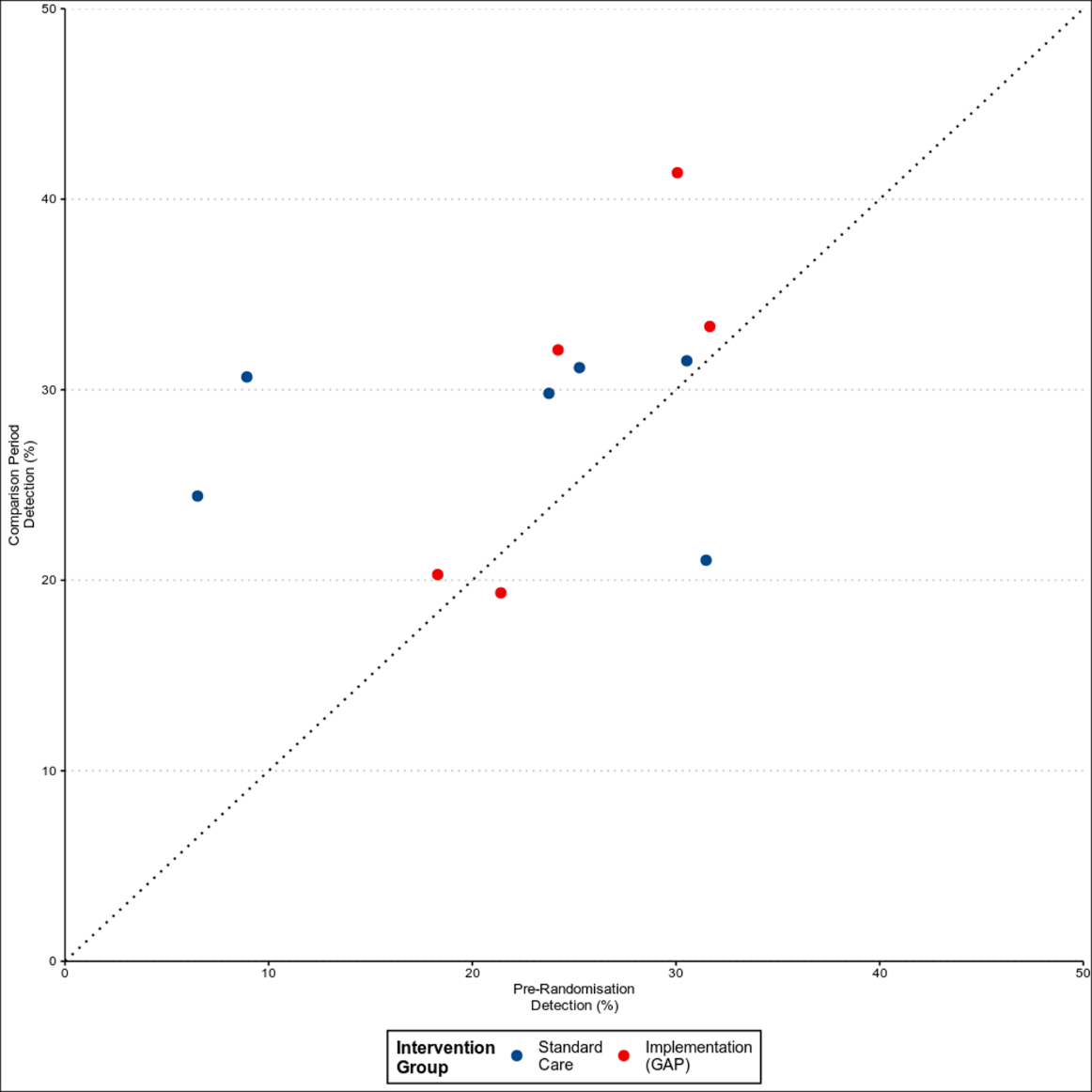


Each point represents a cluster. The doted line represents no change between pre-randomization and comparison period; clusters above the doted line had higher detection in the comparison period (compared to pre-randomisation period) while clusters in below the doted line has lower detection in the comparison period.
