# Supplementary material for: Evaluation of the Growth Assessment Protocol (GAP) for antenatal detection of small for gestational age: The DESiGN cluster randomised trial
Source: PLoS Med. 2022 Jun 21;19(6):e1004004. doi: 10.1371/journal.pmed.1004004 (PMC9212153; doi:10.1371/journal.pmed.1004004)
Supplement: Attachment — Submitted filename: Response to comments 20211117.docx [file pmed.1004004.s006.docx]

**Comments from the Academic Editor:**

This was a very challenging study and unfortunately has limited implications for practice due to the significant logistical challenges. That said it includes some very important messages and articulates many of the challenges associated with complex intervention implementation, and there are important lessons for future studies in this area. There are several issues highlighted by the reviewers which need additional attention - particularly the issue around standard care and the fact that ultrasounds use went up only in the standard care arm. Further detail on the timing of USS scan and detail on acquisition and more importantly validation of the primary outcome is also required.

*Response:*

- *Thank you for consideration of our study for resubmission and for both editors and reviewers for appreciating the value of this first RCT evaluating the effect of GAP. The introduction and use of GAP in the UK and other countries of similar settings have largely been based on observational data and have been subject to much continued debate. This is evidenced by the limited endorsement from national policy makers and clinical guidelines. This trial provides further evidence needed and has been recognised by both guideline developers and the reviewers of this manuscript as an important study in the field. The findings of this study is currently anticipated before publication of the revised Screening and management of SGA guideline by the Royal College of Obstetricians and Gynaecologists.*
- *The comparator of standard care in this trial does include implementation of Saving Babies Lives care bundle; although this raises the issue of comparison with “pure” controls, it reflects real contemporaneous control and is very relevant to current practice (particularly in the UK where we are generalising our findings).*
- *It is likely that the change of ultrasound use observed in the standard care clusters relates to the roll-out of the Saving Babies Lives care bundle, as we explained below in the responses to the reviewers. In this revised submission, we have clarified in detail the methodology for calculating antenatal detection of SGA (for both primary and secondary outcomes) and have also included a brief description of timing of ultrasound use in both arms of the trial.*

**Requests from the editors:**

General comments:

Throughout the paper, please adapt reference call-outs to the following style: "... for high income countries [2,3]." (noting the absence of spaces within the square brackets).

*Response: We have amended the manuscript. We have not tracked this change.*

Data availability:

PLOS Medicine requires that the de-identified data underlying the specific results in a published article be made available, without restrictions on access, in a public repository or as Supporting Information at the time of article publication, provided it is legal and ethical to do so. Since the data are not freely available, please provide an appropriate contact (web or email address) for enquiries (please note that this cannot be a study author).

*Response: We have amended the data sharing statement providing email address for the Department of women and Children Heath at King’s College London (SoLCS_research@kcl.ac.uk), lines 645-646.*

Title: Please revise your title according to PLOS Medicine's style. Please place the study design in the subtitle (ie, after a colon). We suggest "Evaluation of the Growth Assessment Protocol (GAP) for antenatal detection of small for gestational age: the DESiGN cluster randomised trial" or similar

*Response: We agree with the suggestion and have amended the title accordingly*

Please remove the study summary (lines 58-73) and replace with an Author Summary (see below) to follow your Abstract.

*Response: We have transformed the previous study summary into the author summary.*

Abstract:
Please report your abstract according to CONSORT for abstracts, following the PLOS Medicine abstract structure (Background, Methods and Findings, Conclusions) <https://protect-au.mimecast.com/s/6-ghCjZ1N7in8YB2NtxuTAr?domain=eur01.safelinks.protection.outlook.com>

*Response: We have amended the abstract.*

Abstract Background: Please provide the context of why the study is important. The final sentence should clearly state the study question.

*Response: We have amended the abstract.*

Abstract Methods and Findings:

Please provide brief demographic details of the study population (e.g. age, ethnicity, etc) Please be more specific regarding dates of the study for baseline data collection and randomisation. Please include a summary of adverse events if these were assessed in the study.
In the last sentence of the Abstract Methods and Findings section, please describe 2-3 of  the main limitations of the study's methodology."

*Response: We have amended the abstract.*

Abstract Conclusions:

Please begin your Abstract Conclusions with "In this study, we observed ..." or similar, to summarize the main findings from your study, without overstating your conclusions. Please emphasize what is new and address the implications of your study, being careful to avoid assertions of primacy.

*Response: We have amended the abstract.*

Author Summary:

At this stage, we ask that you include a short, non-technical Author Summary of your research to make findings accessible to a wide audience that includes both scientists and non-scientists. The Author Summary should immediately follow the Abstract in your revised manuscript. This text is subject to editorial change and should be distinct from the scientific abstract. Please see our author guidelines for more information: <https://protect-au.mimecast.com/s/OMRECk81N9tO9kK7Gu0WgfQ?domain=eur01.safelinks.protection.outlook.com>
In the final bullet point of 'What Do These Findings Mean?', please describe the main limitations of the study in non-technical  language.

*Response:* *We have transformed the previous study summary into the author summary*

Please rename 'Background' to 'Introduction'

*Response: We have made the change as advised*

Methods:
Please refer to your prospective protocol / analysis plan early in the Methods section. Legends for these files should be included at the end of your manuscript. Changes in the analysis-- including those made in response to peer review comments-- should be identified as such in the Methods section of the paper, with rationale. If a reported analysis was performed based on an interesting but unanticipated pattern in the data, please be clear that the analysis was data-driven.

Please include your Ethics statement within the Methods section.

*Response: We have made the change as advised (lines 198-200).*

Please ensure that the study is reported according to the CONSORT guideline, and include the completed CONSORT checklist as Supporting Information.  Please add the following statement, or similar, to the Methods: "This study is reported as per the Consolidated Standards of Reporting Trials (CONSORT) statement (S1 Checklist)." The CONSORT guideline can be found here: <https://protect-au.mimecast.com/s/CyVxClx1Nji2x1Vy4INaIor?domain=eur01.safelinks.protection.outlook.com>  When completing the checklist, please use section and paragraph numbers, rather than page numbers which will likely no longer correspond to the appropriate sections after copy-editing.

*Response: We have included this statement (lines 331-332).*

Line 223 - please clarify what is meant by ' The utilisation of ultrasound has also been described'

*Response: We have further described the secondary outcomes related to ultrasound utilisation (lines 291-294).*

Results:
Please provide the actual numbers of events for the outcomes (this may be in the Tables, and not necessarily each time they're mentioned).

Please be very clear in the main text which results correspond to which arm.

*Response: Given the use of multiple imputation for primary analysis, actual numbers are not meaningful. Please see response to reviewer #4 (main comments, point 3 below with more detail). All numbers are provided for the available case analysis (S3 Appendix; Supplementary table 4A). We have not amended the manuscript.*

Discussion:
Please present and organize the Discussion as follows: a short, clear summary of the article's findings; what the study adds to existing research and where and why the results may differ from previous research; strengths and limitations of the study; implications and next steps for research, clinical practice, and/or public policy; one-paragraph conclusion.

*Response: We have reorganised the discussion as requested. We have not tracked these changes; no change was made to the text.*

Please remove all subheadings within your Discussion e.g. Main findings Lines 380, 411, 462 - please temper assertions of primacy by adding 'to the best of our knowledge' or similar

*Response: We have removed headings (lines 452;461-464) and have tempered assertions of primacy as advised (line 528 and 572).*

Figures:
Please provide titles and legends for all figures (including those in Supporting Information files).

Tables (including those in Supporting Information files):

Please relabel Box 1 as Table 1

Please present numerators and denominators for percentages.

When a p value is given, please specify the statistical test used to determine it in the table legend.
Please define all abbreviations used in the table legend of each table.

*Response: We have included the statistical test used in each table in the footnote and reviewed all other points. Changes to formatting of supplementary figures/tables are not tracked (there was no change to the contents).*

References:
Please ensure that journal name abbreviations match those found in the National Center for Biotechnology Information (NCBI) databases, and are appropriately formatted and capitalised.
Please also see <https://protect-au.mimecast.com/s/dcICCmO5glujzpgZ4Fwdez2?domain=eur01.safelinks.protection.outlook.com> for further details on reference formatting.

Where website addresses are cited, please specify the date of access.

*Response: We have reviewed the references.*

Supplementary files:

Please provide titles and legends for each individual table and figure in the Supporting Information.
Please see <https://protect-au.mimecast.com/s/DayXCnx1jni7vXPzBhznEyS?domain=eur01.safelinks.protection.outlook.com> for our supporting information guidelines.

*Response: We have combined all supplementary tables and figures into one appendix file (S3 Appendix). We will follow editorial advice if supplementary tables need to be each in a separate file.*

**Comments from the reviewers:**

**Reviewer #1:** Thank you for allowing me to review the submitted manuscript.  The authors are to be commended on try to prospectively study such a complex intervention at scale. There are however a few areas of concern that I think need to be addressed.

Methodololgy
-Despite reading reading this section several times I'm still nor entirely certain how the antenatal detection rate was devised. Was this from retrospective plotting of utlrasound EFWs onto chart or from a sample of notes. For readers to form opinions on the study then this section needs to be very clear and unambigous which it is not in my view currently.

*Response: We thank reviewer#1 for their constructive comments and have addressed the points raised below*

- *We obtained electronic maternity records for all births during the study period. The birthweight centile was then calculated for all pregnancies using – zanthro – package in Stata (UK90 birthweight chart). Customised birthweight centile was also calculated for all pregnancies in the study using the GROW bulk calculator provided by the Perinatal Institute. The definition of which birthweight centile (UK90 and/or GROW) was used is described below separately for primary and secondary outcomes.*
- *We obtained electronic ultrasound records for all pregnancies during the study period. We calculated estimated fetal weight (EFW) from biometry where it was not already available in the ultrasound dataset. The EFW centile was then calculated according to the fetal growth chart applicable to each study arm and period of study (Hadlock chart used during pre-randomization in all clusters and in standard care clusters during the outcome period; GROW charts were used for intervention arm clusters during the outcome period). Hadlock centiles were calculated in Stata using the Hadlock formula (Hadlock FP, et al. In utero analysis of fetal growth: a sonographic weight standard. Radiology. 1991 181(1): 129-133). GROW centile were calculated using the bulk calculator provided by the Perinatal Institute.*
- *Antenatal detection was calculated as the proportion of babies confirmed as SGA at birth (birthweight centile; denominator) that were identified as SGA antenatally by ultrasound (EFW centile; numerator). For clarity, false positive cases (SGA by estimated fetal weight but not SGA by birthweight) were not included in the numerator, as they were not part of the population of interest (denominator includes only babies confirmed to be SGA at birth).*
- *For the primary outcome the definition of SGA at birth was infants that had birthweight <10^th^ centile by both the UK90 and GROW charts; these infants are the only ones that are targeted by both strategies being assessed in this trial. SGA by ultrasound EFW was defined as EFW<10^th^ centile using the Hadlock chart for babies born in standard care clusters during all the study periods. In the intervention arm clusters, SGA by ultrasound EFW was defined as EFW<10^th^ centile using GROW chart in the intervention arm during the outcome period and as EFW<10^th^ centile using Hadlock charts in the pre-randomisation period. For clarity, SGA by ultrasound was determined solely by the definition being used in the trial arm and time period to assess full performance of each screening strategy/intervention (SGA by both customised and population chart was not applied in EFW centiles). Given the use of SGA at birth by both definitions, false positive rates are not clinically meaningful/interpretable (this would include babies who were actually SGA at birth by either the UK90 charts, or the GROW charts, but not by both). Therefore, the test positive rate was reported for the primary outcome; the test positive rate does not depend on the definition of SGA at birth being used.*
- *For secondary outcomes of SGA at birth by customised centiles, SGA at birth was defined as birthweight <10^th^ centiles using GROW chart for all pregnancies in the trial (both intervention and standard care arm). SGA by ultrasound EFW was defined in the same way as for the primary outcome.*
- *For secondary outcomes of SGA at birth by population centiles, SGA at birth was defined as birthweight <10^th^ centiles using UK90 chart for all pregnancies in the trial (both intervention and standard care arm). SGA by ultrasound EFW was defined in the same way as for the primary outcome.*
- *This detailed description has been included in the supplementary material (S2 Appendix; page 3. Changes were not tracked in the appendix) and a statement was added to the methods section of the manuscript (lines 276-280). This pre-specified approach was reported in our protocol publication (Vieira MC, et al. The DESiGN Trial (DEtection of Small for GestatioNal age fetus), evaluating the effect of the Growth Assessment Protocol (GAP): study protocol for a randomised controlled trial. Trials. 2019 Mar 4;20(1):154. doi: 10.1186/s13063-019-3242-6.)*

Standard care. There is no attempt to define what GAP is being compared and it is therefore difficult to judge whether GAP had a realistic chance of success (see below) following intervention. In my view the authors should provide more detail on what standard care is and whether this was within certain parameters or very variable. It is interesting to note that the standard care arm seemed to changes more significantly in terms of scan frequency than the GAP arm. There is also no attempt to describe whether units were secondary or tertiary or involved in other measures to improve SGA detection.

*Response:*

- *There was no pre-specified protocol for the standard care. At the start of the trial, national recommendations were provided by the RCOG guideline on screening and detection of SGA. We have summarised the recommendations in lines 240-249. Given the lack of a pre-specified protocol to benchmark compliance, primary data collection through review of a sample of notes was not performed in the standard care arm (as was performed in the intervention arm). This is a pragmatic based trial on ‘standard care’; we believe attempting to ‘standardise’ standard care in this study would create an artificial comparison and potential for change of practice given the study processes (contamination). Even though we agree it is challenging to ascertain based on our trial to what is an ‘optimal’ standard practice, we do believe practice in standard care clusters reflected national practice in maternity units not implementing GAP, including national variation. Indeed, local guidelines largely followed national recommendations, albeit with some degree of variation. Information obtained from review of local guidelines was included in a separate detailed implementation manuscript and has not been included in this submission given not based on primary data from pregnancies. We would follow advice from editors if this need to be included in this submission.*
- *Shortly after the launch of the DESiGN trial, the Saving Babies’ Lives care bundle was rolled out, changing national recommendations. Even though clusters in this study were exempt from the fetal growth restriction component of the care bundle, other components of the care bundle and awareness of stillbirth in the media may have influenced practice in clusters from the standard care arm of this study. This may have resulted in the increased use of ultrasounds observed in the standard care arm. Indeed, The SPiRe study (Widdows K, Roberts SA, Camacho EM, Heazell AEP. Evaluation of the implementation of the Saving Babies’ Lives Care Bundle in early adopter NHS Trusts in England. Maternal and Fetal Health Research Centre, University of Manchester, Manchester, UK. 2018.), which evaluated the impact of the introduction of the Saving Babies’ Lives care bundle, described that introduction of the care bundle was associated with an increase in the use of ultrasound. This is in keeping with our findings from the standard care arm. We have clarified in line 471 that the care bundle was associated with increased ultrasound use.*
- *We used size of hospital as a proxy measure for complexity of units. We can confirm there were 4 tertiary units in the trial and they were balanced by randomization (2 in each arm). We have added a sentence in the results (lines 393-394).*

Results
The primary outcome table 3 is not very easy to read or interpret in its current format and I would suggest reviewing the row legends.

*Response:* *We have amended the row legends in table 3.*

Is there any capacity to perform a sensitivity analysis using scans performed within 3 weeks of birth rather than performed throughout the 3rd trimester.

*Response: A sensitivity analysis using scans within 3 weeks of birth would invariably reduce the antenatal detection as all women with scans with a larger interval would be considered not detected (given this is a population level analysis these women cannot be excluded). An alternative approach restricting the analysis only to women with a scan within 3 weeks of birth (women without a scan excluded), would not be representative of the population (high risk group) and therefore not generalisable. We would like to highlight we are assessing the performance of a complex intervention in the population and not the accuracy of ultrasound. We have not made changes to the manuscript at present.*

Discussion
Whilst the populations are similar there are some signficant differences and these are not discussed. From my reading of table 1 it would appear that the sites where the intervention occurred had more pakistani women and this might potentially allter the sensitivity of grow/standard care.
Table 2 demonstrates some surprising findings that are difficult to understand/believe for example the number of women not receiving anatomy scans between 18-24 weeks seems very low. There is also a huge increase in >24 weeks scan frequency in the standard care arm which is not even mentioned. This increase means that 77% of women in standard care have a scan though there is no description of when during the third trimester these scans occur. The big problem here in my mind is that the primary function of GAP is not managing women who are having growth scans, but improving the detection of SGA in low risk women, by more accurately targetting scans. However when scan frequency is so high it seems unlikely any package of measures will have an effect on SGA detection rate. The authors should discuss this as it is a mojor limitation of the primary outcome.
With such high % of scan usuage it is surprising that the detection rate was so low which suggest lots of process problems as briefly discussed in terms of historical scan timing. It is however difficult to judge the effecrtiveness of GAP if the recommended scan frequency has not been implemented. Of interest there does not seem to be a big difference between customised and non-customised outcomes in any metric. This is potentially the most interesting finding as although I'm not convinced the authors have adequately tested GAP, they have demonstrated that customisation does not really make a difference to any outcome. This should be highlighted and discussed in my view.
I note the stillbirth seems lower in the intervention arm, but no mention is made in the discussion about the pre-implementation phrase during which the standard care group had higher levels.

*Response:*

- *We described differences in demographic characteristics between intervention and standard care arm in lines 386-390. Imbalances may be related to the modest number of clusters in the trial. To mitigate differences in relevant factors such as ethnicity, the analysis plan included adjustments in the calculation of the adjusted cluster summary values and hence the intervention effect. Of note, such adjustments do not affect or influence the customisation process.*
- *As for any randomised control trial, the aim of this study was to compare the effect between arms. We did not pre-specify in the analysis plan the assessment of any potential changes of demography, resources or outcomes within each arm between pre-randomisation and outcome periods. The purpose of the pre-randomisation data was to provide adjustment for cluster characteristics and detection rates in the comparison of primary and secondary outcomes between the intervention and standard care arms (with the aim of improving power for analysis).*
- *Despite the difference observed in ultrasound use in the pre-randomization period, there was no difference between arms in the outcome period. The mean number of scans per pregnancy after 24 weeks (with EFW) was numerically the same (1.5 per pregnancy) and the high proportion of pregnancies with scan after 24 weeks was observed both in intervention and standard care arm (66% vs 77%). If the intervention were better at targeting scans in low-risk women, a significantly lower proportion should have been observed in comparison with standard care. We don’t agree that the high use of ultrasound is a limitation of the study. However, we agree that this does need to be considered when assessing generalisability of the findings.*
- *In response to the interest from 3 reviewers and the academic editor with regards to ultrasound use, we have now included a descriptive analysis of timing (gestation) of ultrasound utilisation. No clear difference was observed between arms during the outcome period and this confirmed the common practice in the UK of offering scans at 28, 32 and 36 weeks. In the pre-randomisation period, a higher proportion of women giving birth in the intervention arm had a scan at 36 weeks. We have added this in the methods (lines 294-296), results (lines 398-402; Figure 2) and discussion (lines 512-519). We would like to emphasise that the findings from pre-randomisation are not the primary or secondary endpoint of this trial and should be considered only for their value as an adjustment factor.*
- *We included a sentence in the discussion stating the trial findings suggest the use of a customised chart has limited influence in the detection of SGA (line 520-522).*
- *We have stated that perinatal mortality rates were lower in the intervention arm compared to the standard care arm during the outcome period (clarification now added to line 502); we have not made any comparison between the pre-randomization and outcome periods (for primary or secondary outcomes) for reasons explained in previous responses. Stillbirth and perinatal mortality are strongly correlated (as stillbirth accounts for most of perinatal mortality in our trial). We have been cautious in the interpretation and discussion of stillbirth and perinatal morbidity findings given these are two of 26 secondary outcomes. Two of the four reviewers (including the statistical reviewer) agreed with our approach. We have not made changes to the manuscript with regards to stillbirth/perinatal mortality findings.*

Summary
This study has potential practice chaging findings and I am very keen to see it published, but currently there are some significant changes required in my view prior to publication.

*Response:* *Thank you for your comments and for emphasizing the clinical value of this trial.*

**Reviewer #2:** Statistical review

This paper reports a cluster randomised trial investigating implementation of the Growth Assessment Protocol on detection of small for gestational age during pregnancy.

The trial was reported well and I had only minor comments.

1.      Abstract "(instead of patients or subjects)" - presume this was leftover text?

*Response: We thank Reviewer #2 for their feedback and comments for change. Thank you for pointing this out. We have amended.*

2.      Abstract - providing a brief summary of the low observed false positive detections of SGA might be of interest to aid interpretation of the results.

*Response:* *We have adhered to the CONSORT statement for abstracts that recommends reporting only the primary outcome in the abstract. We will follow editorial advice on this.*

3.      Page 14 line 314 'with obesity (15.7% vs 18.1%)' I initially read this as restricted to women of white ethnicity - a colon after 'less women' might help avoid misreads.

*Response:* *Thank you,* *we have amended the manuscript (line 387).*

4.      Page 15 line 343 - I feel it should be pointed out here that two marginally significant secondary outcomes out of 26 is consistent with what would be expected by chance if there were no benefit of intervention for any of the outcomes (to be fair the authors do point out this in the methods).

*Response: We agree with your comment. However, as this is stated in the methods section, we have not duplicated this information in the results. There is also a sentence in the discussion stating replication of this finding is needed given the number of secondary outcomes (line 510-511). We have not amended the manuscript.*

5.      Discussion: Table 2 seems to show huge improvements in control clusters during the outcome period, compared to the baseline period. This might be worth reflecting on in the discussion. It wasn't clear to me why there were generally big differences between control and intervention clusters in the pre-randomisation period though?

James Wason

*Response:* *Differences between arms pre-randomisation are explained by the modest number of clusters in the trial.* *As per previously stated (Reviewer #1, discussion, response to point number 2), the aim of this trial was to compare the effect between arms and we do not conduct any formal testing of change over time. We do now explicitly mention the increase in detection in the standard care arm compared to the pre-randomization period in the discussion, which we relate to the introduction of the Saving Loves care bundle. Further analysis describing timing of utilisation of ultrasound has been included in the revised manuscript as explained in our previous response (Reviewer #1, discussion, response to point number 4).*

**Reviewer #3:** This is a well designed and executed study assessing the utility of the GAP program is the detection of fetal growth impairment (Defined as SGA, <10thcentile). The study was complicated by the roll-out of the Safer Baby Bundle, albeit the participating hospitals were exempted. The authors acknowledge that there would have been some contamination from that roll-out.
The methodology is well described and appropriate. The results are appropriately reported.
I would make the following comments:

1. The standard care hospitals appeared to have a significant change in key outcomes from the pre-randomisation phase to the outcome period (ie % pregnancies with 18-24 week scan; % pregnancies with a scan >24 weeks; % pregnancies with a scan >24 weeks +EFW; % pregnancies with no scan). these changes suggest changes in care provision between these two times, in the direction of improved care. The authors don't discuss this effect. Could this not explain why there is no difference in outcome between the two groups of hospitals? A discussion of this would be useful.

*Response: We thank Reviewer #3 for their positive feedback and for emphasising the appropriateness of our methodology and report of results*

- *As previously stated (Reviewer #1, discussion, response to point number 2), the aim of this trial was to compare the effect between arms and we did not pre-specify in the analysis plan the assessment of demography, resources or outcomes within each arm between baseline period and outcome period.*
- *The change in utilisation of ultrasound does not necessarily infer improved care, but we discuss the increased use of scans over time and the likely impact of introducing the saving lives care bundle.*
- *Please see response to previous reviewer regarding additional descriptive analysis of timing of ultrasound utilisation (Reviewer #1, discussion, response to point number 4).*

2. The authors report that the rate of stillbirth and perinatal mortality were reduced in the intervention group of hospitals but not the standard care group. What they don’t comment on is that the rates fell to those of the standard care hospitals. Nonetheless, this is an important finding. As the authors comment, the whole point of detecting SGA is to reduce stillbirth. I think this finding should be in the Abstract and in the Main Findings section with further discussion in the Implications.

*Response:*

- *As per previous response (Reviewer #1, discussion, response point number 6), we have stated that stillbirth/perinatal mortality rates were lower in the intervention arm compared to the standard care arm (clarification now added to line 502); we have not made any comparison between pre-randomisation and outcome period (for primary or secondary outcomes) for reasons explained in previous responses. We have been cautious in the interpretation and discussion of stillbirth and perinatal morbidity findings given these are two of 26 secondary outcomes. Two of the four reviewers (including the statistical reviewer) agreed with our approach. We have not made changes to the manuscript with regards to stillbirth/perinatal mortality findings. We will take editorial advice.*

3. The intervention led to earlier birth than standard care. This is a common finding among studies exploring better detection of SGA. Indeed, in this study the false positive rate increased in both groups too. Earlier delivery of SGA, but not false positive AGA, effect has potential to cause longer term cognitive harm. This was recently reported in JAMA (Selvaratnam 2021;326(2):145-153). A comment on the possibility of harm - not just lack of utility - would be important.

*Response: We added a sentence regarding effect of earlier delivery of SGA infants (line 506-509).*

**Reviewer #4:** This cluster randomized trial evaluated the effectiveness of implementing the GAP program for antenatal detection of SGA births in maternity care hospitals in the UK. The research question is a highly important one, as this program appears to have been widely adopted into routine care across the UK without high-quality evidence of its effectiveness, and findings from this study suggest that the program may not in fact be achieving its intended objective. The trial was a pragmatic one, evaluating the effectiveness of the program as implemented in a real-world setting (as opposed to evaluating the efficacy of the program if implemented under optimal conditions), which is most relevant to informing roll-out of the program nationally and internationally.
The study findings are a challenge to interpret because of several issues arising during the trial, most notably, the concurrent national introduction of the Saving Babies Lives care bundle. The adoption of this care bundle means that the type of care provided to the standard of care comparison group likely changed across the study period, making it unclear to what clinical care settings the adverse outcome rates in the comparison group are generalizable to, and decreasing the value of the information on baseline rates. I do sympathize with the investigators, as the unfortunate timing of introduction of this care bundle was beyond their control, and as a new trial to address this question now seems unlikely, believe the trial nevertheless provides the best available information to help assess the merits of this program.

Main comments

1) My first concern relates to the two stage cluster-summary statistical analysis approach used, primarily as it relates to the less common secondary outcomes. If I understand correctly (without remote access to the textbook cited in the Supporting Information), the first stage of the two-stage cluster analysis calculates a summary value for each site (i.e., a site-specific mean rate (percent) of each outcome), adjusted for the listed covariates, and these rates (percents) are then put into a linear regression model estimating the difference in rate (percent) between treatment and control groups in stage 2. My concern is that for uncommon secondary outcomes, the site-specific rates estimated in step 1 will be very unstable because of low numbers of events at each site. For example, with 11,096 births in the intervention arm, and a stillbirth rate of 3.1 per 1000, there will only be ~34 stillbirths across all 5 intervention sites, meaning that the site-specific rates are being estimated from only 6-7 events per site. One stillbirth more, or less, per site will have an outsized impact on a site's rate, which in turn, will have a major impact on the comparison of rates between sites given the small number of sites.

I am not a trial statistician, but it would seem to me that for rare outcomes, an approach should be chosen that does not depend on accurate estimation of the hospital-specific rates. I agree that a multi-level model might be challenging given the small number of clusters, but is it not possible to build a single model using the original individual-level data that controls for clustering by hospital using GEE (or even an indicator variable for hospital), and uses marginal estimation to calculate risk differences between treatment arms (e.g., as per the approaches compared here: <https://protect-au.mimecast.com/s/d469CoV1kpfrEDgMWHmPyQf?domain=eur01.safelinks.protection.outlook.com>). Please better justify the choice of analytic approach as it relates to rare outcomes.

*Response: We thank Reviewer #4 for their comments and recognising the importance of this study and the limited robust evidence from clinical trials to support use of GAP.*

- *The essence of the referee’s concern about the two-stage method seems to be that it depends on accurate estimation of hospital-specific rates, which would be problematic for a low event outcome as the observed rate for a hospital could be influenced by adding or removing just one event. However, we disagree that the method requires accurate estimation of hospital-specific rates or that the challenge of low event rates affects the two-stage method more than a one-stage method. We see the one and two-stage methods as fundamentally closely related. Both compare the average outcome value (here an event ‘rate’) between arms, pooling data across hospitals. There are subtleties about the ‘weighting’ of this average if hospitals vary in size (see next comment) which differ between one and two-stage methods. The one-stage method is generally preferred because it is slightly more statistically efficient (powerful) if hospitals vary in size and because of the ease of adjusting for individual covariates. We selected the two-stage method because of the known problems with one-stage methods when, as in this trial, the number of clusters is modest.*

2) On a related note, please expand the description of the analytic approach in the Supporting Information to clarify how the two-step approach accounts for differences in cluster sizes. That is, the site-specific rate from a large site will be more precise than that from a small site; were the summary values in the second step weighted to account for the number of births at that site?

*Response: We agree with the author that the hospital-level rates will be more precisely estimated in larger hospitals, and we are aware that some researchers advocate weighting the hospital-level rates in analysis by either the inverse of the variance of the estimate or by the ‘size’ (number of births in this context) in preference to an unweighted analysis. Weighting by the inverse of the variance of the estimate (not just the ‘within cluster’ variance but the variance accounting also for variation between clusters) is complex but theoretically optimal and should provide some greater efficiency if hospitals vary in size. Weighting by the hospital size leads to inference for the ‘population of births’ but is not statistically optimal. Given these issues, and in our opinion no clear favourite between these approaches in the literature, we applied an unweighted analysis. We have clarified this in the methods (line 311).*

3) The amount of missing data in this trial is concerning. Although I agree that using existing hospital databases can be a reasonable strategy for efficiently collecting participant and outcome data for a large number of trial participants, the amount of missing data in this trial was much higher than I typical expect from clinical perinatal databases (e.g., 37% missing values for a key pregnancy complication such as pre-eclampsia during the outcome period in the standard of care group), and for variables that should not have missing data (e.g., vital status at discharge [neonatal mortality, stillbirth], based on the lack of 'n' provided in Table 4). This raises concerns for me about data quality and the appropriateness of multiple imputation given high rates of missingness. What efforts were made to reduce missing data and ensure data quality? Were chart audits considered to eliminate missing data for important health outcomes such as stillbirth and neonatal mortality?

*Response:*

- *We would not wish to deny that missing data is a concern for this trial, but in our opinion applying multiple imputation (at least if there is information recorded which is associated with the variable subject to missing data and can therefore be used in prediction) will in general be preferable to analysing the available cases only, an approach which requires stronger assumptions. Given the complexity of the data and agreeing with the reviewer regarding the relevance of this point, we have published a detailed report of data management, including its challenges and action undertaken to mitigate limitations (Relph S, et al. Using electronic patient records to assess the effect of a complex antenatal intervention in a cluster randomised controlled trial - Data management experience from the DESiGN Trial team. Trials 2021 Mar 8;22(1):195. doi: 10.1186/s13063-021-05141-8). Brief response to specific questions in this point are provided below:*
- *Limitations with regards to availability of data in electronic patient records for previous medical comorbidities and obstetric complications are known; National Maternity Perinatal Audit has previously described this (https://maternityaudit.org.uk/pages/reports). Out findings are consistent with the findings from the NMPA report. However, missing data for these characteristics does not influence the effect of the intervention or the value of the trial, just limits our ability to describe the study population in more detail.*
- *Counts (‘n’) are not provided for stillbirth/perinatal mortality and many other variables as primary analysis was undertaken using data with multiple imputation; therefore, counts are not meaningful as they represent the average of 10 datasets (there could be 4.55 stillbirth, whereas in real life there can only be 4 or 5 stillbirth). For transparency we provided counts (‘n’) for all variables in the supplementary tables with available case analysis (S3 Appendix; Supplementary Table 4B). In particular for stillbirth/perinatal mortality, there were 95 pregnancies with missing data (combined pre-randomization and outcome period for both arm of the trial); this represents 0.12% (95/80,856) of all pregnancies. Even though these variables shouldn’t have missing data, we believe the risk of this influencing the trial results for this secondary outcome is minimal.*
- *Regarding efforts to improve data quality, we performed a quality assessment of data for all clusters at the start of the trial and provided feedback to improve data collection. We noted that levels of missing values improved after this exercise. We re-obtained data from the pre-randomisation period again later in the trial to ensure we had the best quality data possible. Results of improvement of data quality based on our processes have been previously described in the data management paper from DESiGN referenced above.*

4) The Supporting Information section on data management states, "For one cluster, ultrasound measurement data were not available for the baseline period. The proportion of SGA infants detected antenatally (by both definitions) at baseline for this cluster was imputed based on a model fitted to data from the other clusters predicting the number of infants detected based on the number of pregnancies with an ultrasound scan after 24 completed weeks". Imputing values for an entire site with missing data does not seem like a good solution given the between-site variation observed in the study. Did the study team consider conducting a chart review for a random sample of deliveries from this site to at least have some real data from the site to include in the imputation model?  Further, more information on this lack of a key outcome variable seems important given the adjustment for baseline values in the final model: a) was this cluster in the treatment or control group, and b) were sensitivity analyses done excluding this cluster from analyses?

*Response:*

- *We acknowledge the concern about this, but as explained in our previous response, we believe imputation is helpful here (in comparison to alternative approaches) because we have information on the number of pregnancies with an ultrasound scan completed after 24 weeks which we see is strongly associated with the proportion of SGA infants in other sites.*
- *We did consider reviewing a sample of notes but due to the start of COVID-19 pandemic with healthcare pressures and national lockdown, it was not possible at that time. We felt it was not appropriate to delay trial reporting and opted for imputation of the pre-randomisation estimate for that cluster as performed.*
- *The cluster in question was in the intervention arm. We have performed a post hoc sensitivity analysis excluding this cluster and results are consistent with primary analysis. We have added a sentence with this information in the methods (lines 313-315) and results (lines 414-417)*

5) While appreciating that an economic analysis is likely beyond the scope of this manuscript, it does seem important to include some mention of the costs of this intervention. If a program is free, shows no evidence of causing harm, and there is a suggestion that it might improve an important health outcome like perinatal mortality, one might argue that it is not unreasonable to still implement the program despite the lack of evidence for its effectiveness, given the potential for benefit. However, if the program uses a meaningful amount of resources, then implementation of this program is much less justifiable, given that it would be detracting resources from other proven interventions. Can the authors comment in general terms on the costs of GAP implementation for those readers less familiar with the program? If implemented outside of the trial setting, does the Perinatal Institute provide its services for free, or are hospitals required to pay for the Institute's support? How much staff time was required/recommended for training and implementation? For ongoing running of the program?

*Response:*

- *There is a cost related to implementation of GAP which includes both direct costs, such as the training fee (one off) and the annual fee charged by the perinatal institute, and opportunity cost, including time out of clinical practice to train the whole maternity workforce. We have added a sentence in the discussion (lines 497-500).*
- *Full economic analysis has been subject to detailed analysis in the DESiGN trial. This has been planned as a separate manuscript given the breadth of data and analysis undertaken. We are currently finalising that manuscript and we would follow editorial advice with regards to the interest from PLOS Medicine to receive submission of that manuscript.*

6) The trial findings were not entirely unexpected, given previous literature demonstrating that customised growth charts (the core element of the GAP protocol) are not meaningfully better at identifying high-risk infants than non-customised ultrasound fetal growth charts (customised charts generally only appear to be better at identifying high risk infants when compared to birthweight charts [i.e., charts derived from the weights of live births rather than estimated fetal weights from ongoing pregnancies], which do not reflect the charts used antenatally to monitor fetal growth and are biased at preterm ages because of the correlation between preterm birth and fetal growth restriction). Citing some of this literature in the Discussion, particularly those studies comparing ultrasound estimated fetal weights classified using customised and non-customised standards, seems relevant to help interpret the plausibility of trial findings. E.g., Am J Obstet Gynecol 2018:218:S738-S744; Am J Epidemiol 2011;173:539-543.

*Response:*

- *We have included in the discussion previous evidence suggested by the reviewer (lines 522-527). It is worth noting though that the previous evidence assess the value of customised centiles alone (not as part of GAP).*

Minor comments

1) It would be helpful to include a diagram of the study timeline in the Supplementary Material (pre-intervention, implementation, washout, and outcome collection periods by calendar time at each of the sites) - the description in the main body of the text was somewhat hard to follow, and given the concurrent roll-out of the Saving Babies Lives care bundle, it would be helpful to know the calendar timing of outcome collection at trial sites in relation to this bundle.

*Response: Providing a timeline for each cluster would have the potential of identifying individual units through both this and our separate implementation report. As per our ethics submission and study protocol, we have to ensure clusters are not identifiable. We have therefore not included it in the manuscript. With regards to the care bundle, it was rolled -out in 2016. The outcome period for all clusters was in 2018/2019, therefore well into the implementation of the care bundle. We have added a sentence about the time of implementation of the care bundle (lines 471-472).*

2) Please confirm that the study outcomes were included in the imputation models in the description of multiple imputation in the Supporting Information.

*Response: We can confirm that the study outcomes were included in the imputation models. We have clarified this in the S2 Appendix (page 4; changes not tracked).*

3) Presentation of results: an "adjusted difference 2.2%, 95% CI -6.4% to 10.7%" could be interpreted by some to be a relative change (i.e., an increase of 2.2%) rather than an absolute change in percentage points (which I believe is the correct interpretation based on a linear regression model). If so, the authors may wish to rephrase this as "2.2 more SGA infants detected per 100 births" for clarity for this and all outcomes.

*Response: The reviewer is correct in the interpretation that results are absolute change/difference. We have clarified in the methods that results reported are absolute differences (line 312) and explicitly stated in the results that differences are intervention minus control (line 404; line 412; line 416). We would take guidance from the academic editor if the wording for reporting results should be changed throughout the manuscript as we believe general readership are more used to current presentation of results.*

4)      The stillbirths are of considerable interest, and although the authors were appropriately cautious in interpreting the results for this outcome given the number of secondary analyses conducted, I believe that readers may be tempted to focus on this outcome. Including more information on these cases would be helpful, such as presenting the counts in each group (and counts of missing data), as well as a summary of cause of deaths.

*Response: In response to an earlier comment (Reviewer #4, main comments, point 3), counts cannot be given for the primary analysis given datasets with multiple imputation were used. For available case analysis (S3 Appendix; supplementary table 4A), the counts in each group are provided and the missingness can be calculated (0.12% missingness for perinatal mortality/stillbirth when combining data from pre-randomisation and outcome periods).*

5) The references in the Supporting Information appear to be incomplete; please update instances of "ref Mx" and "ref data Mx" (page 3) with the appropriate citation.

*Response: Thank you for pointing this. We have amended the S2 appendix (changes not tracked).*
