# Supplementary material for: Evaluation of the Growth Assessment Protocol (GAP) for antenatal detection of small for gestational age: The DESiGN cluster randomised trial
Source: PLoS Med. 2022 Jun 21;19(6):e1004004. doi: 10.1371/journal.pmed.1004004 (PMC9212153; doi:10.1371/journal.pmed.1004004)
Supplement: Attachment — Submitted filename: Response to comments 202200209.docx [file pmed.1004004.s007.docx]

**Comments from the reviewers:**

Reviewer #1: Thank you for the detailed responses and changes made to the manuscript in response to mine and others reviews.

My remaining area of concern regards the information on standard care provided in the mansuscript in line 215-229 and in the response to review.

The authors state that-

"This is a pragmatic based trial on 'standard care'; we believe attempting to 'standardise' standard care in this study would create an artificial comparison and potential for change of practice given the study processes (contamination). Even though we agree it is challenging to ascertain based on our trial to what is an 'optimal' standard practice, we do believe practice in standard care clusters reflected national practice in maternity units not implementing GAP, including national variation."

-and then go on to state that a detailed review of the standard care local guidelines is already available in a prior publication (not referenced). Having looked for this I cannot easily find it to assess whether the reader can determine the range of standard care applied, but still think more information needs to be provided in this manuscript. Given there are only a small number of units in the study standard arm it would not require much additional work to include for example how many units employed uterine artery Dopplers or not, whether surveillance scan frequency was 3, 4 weekly or longer or prespecified at certain gestations, and gestation of last planned surveillance scan. This information could all be included in a simple supplementary table. Due to the international reach of PLOS med and the fact that this study's findings will be of interest to a range of healthcare systems outside of the UK I do not think quoting the RCOG guidelines and potential adherence to this is sufficient and still requires further work.

**Response**

- *We have now included in the S2 Appendix (page 2) a summary description of screening strategy in the standard care. A summary description was chosen (instead of a table) to avoid identification of participating clusters. Of note, this information was obtained based on review of their guidelines; this has been described in the methods section (lines 224-226).*

Reviewer #2: Thank you to the authors for addressing my previous comments well. I have no further issues to raise.

**Response**

- *Thank you for reviewing out manuscript.*

Reviewer #4: I appreciate the authors' thoughtful and detailed responses, which have satisfactorily answered most of my initial concerns.

I do, however, remain concerned about the use of the two-stage approach in the context of rare events (i.e, estimation of site-specific rates based on very low counts of events). The authors describe the two-stage approach in their Statistical Analysis (supporting information) as: "Firstly, the cluster summary values were adjusted for the ethnicity, age and parity of the individual participants (these are residuals from comparing the observed summary values with those predicted from a model fitted to all participants)... In the second stage, linear regression analysis (ANCOVA) was undertaken in which the adjusted cluster-summary values for an outcome in the trial outcome period were compared between intervention and standard care arms..." It is thus unclear to me how the response to reviewer comments can then claim "However, we disagree that the method requires accurate estimation of hospital-specific rates"-- unless I am misunderstanding, the adjusted hospital-specific rates are precisely what is being compared between groups, so an inaccurate or unstable rate would be expected to influence results. Especially if there is uncertainty in the literature as to which is the best approach, it would be helpful to include results showing the results when using a one-stage approach as supporting information. Confirmation that findings are robust to the type of analytic method would greatly strengthen confidence in the study conclusions.

**Response**

- *We maintain* *our opinion that low event rates do not affect two-stage methods more than one-stage approaches, and that the two-stage approach we selected is superior where the number of clusters is modest as in this trial. One-stage methods are liable to non-convergence and may generate inappropriate standard errors. We also note that we selected the two-stage method a-priori and this request for a different analysis approach was made after having seen the results from the first analyses. Nevertheless, we have now included a further sensitivity analysis as suggested* in case any other reader poses the same question regarding the stillbirth finding*. Our preferred one-stage approaches were unfeasible or did not converge, but we report the results from a simple approach that uses ‘robust standard errors’. We have added text in the methods to summarise the analysis approach (lines 303-307), and details to the statistical analysis section of the S2 Appendix (page 6). We report the resulting odds ratios in the results (lines 405-406) and note in the discussion that the analysis did not confirm the findings from our primary approach (lines 480-481). We have not changed our interpretation of the results based on this additional sensitivity analysis given that we had previously discussed the possibility of a chance findings (479-480) and the need to replication of the stillbirth/perinatal mortality finding (487-488).*
