# Supplementary material for: Evaluation of the Growth Assessment Protocol (GAP) for antenatal detection of small for gestational age: The DESiGN cluster randomised trial
Source: PLoS Med. 2022 Jun 21;19(6):e1004004. doi: 10.1371/journal.pmed.1004004 (PMC9212153; doi:10.1371/journal.pmed.1004004)
Supplement: Attachment — Submitted filename: Response to comments 20220426.docx [file pmed.1004004.s008.docx]

**Requests from Editors:**

General comments:

To help us extend the reach of your research, please provide any Twitter handle(s) that would be appropriate to tag, including your own, your coauthors’, your institution, funder, or lab.

**Response**

- *We have included the Twitter handles (*@lifecourse_KCL, @GSTTCharity, @SandsInsights, @tommys) *in the submission website.*

Data availability:

The Data Availability Statement (DAS) requires revision. Please remove “request made to the Chief Investigator (Prof Dharmintra Pasupathy) as, in the interests of transparency and reproducibility, a study author cannot be a contact person for the data or be responsible for approving access to data.

**Response**

- *We have amended the manuscript (lines 634-635).*

Abstract:

Abstract Background: please temper assertions of primacy by adding ‘to the best of our knowledge’ or similar with relation to ‘there are no reported randomised control trials.’

**Response**

- *We have amended the manuscript (line 59).*

Abstract Methods and Findings:

Please clarify whether this should be November 2015 or 2016 (noting discrepancy between the Abstract and Methods section) Please add a sentence to summarise the secondary outcomes Please revise to <10th centile Please define CI at first use

**Response**

- *The trial started on November 2016 but electronic data was obtained for women that delivered up to one year prior to randomization, as stated in the methods section. For shortness, the abstract only reported the period for which data was collected from women. We have now clarified the information in the abstract (line 65). We were asked about these dates and protocol registration when we were invited for full submission of our manuscript; as per our previous response, we will follow editorial guidance on the appropriate wording regarding dates of study start and data collection.*
- *We have included a brief description of the secondary outcomes on the abstract (lines 74-75).*
- *We have amended the manuscript regarding ‘<10^th^ centile’ (lines 72-73) and defining CI at first use (lines 86)*

Author Summary:

Please temper assertions of primacy by adding ‘to the best of our knowledge’ (or similar) with relation to ‘This first randomised control trial’

In the final bullet point of ‘What Do These Findings Mean?’, please describe the main limitations of the study in non-technical language.

**Response**

- *We have amended the assertion of primacy (line 112) and have included the main study limitations (lines 126-128).*

Methods

Thank you for providing a CONSORT checklist. When completing the checklist, please use section and paragraph numbers, rather than page numbers which will likely no longer correspond to the appropriate sections after copy-editing.

**Response**

- *We have amended the CONSORT checklist by removing the page number and keeping only the section name/paragraph number.*

Results:

Line 367 (and throughout) - please revise to ‘there were fewer women..’, ‘fewer stillbirths’, etc Where adjusted analyses are presented, please specify which factors are adjusted for, and provide the unadjusted analyses.

**Response**

- *We have amended the wording (lines 375, 415 and 417) and include the unadjusted analysis where appropriate. We have not included unadjusted analysis for post-hoc analyses.*

Discussion:

Line 443 - please temper assertions of primacy by adding ‘to the best of our knowledge’ or similar

**Response**

- *We have amended the manuscript (line 456).*

Tables:

Tables 2, 3, 4, 5, S3A, S3B, S3C, S4A, S4B - When a p value is given, please specify in the table legend the statistical test used to determine it.

Tables 3, 4, 5, S1C, S3A, S3C, S4B - Please present numerators and denominators for percentages Supplementary Table 2 appears to be missing

**Response**

- *We have clarified in each table legend that p-values are provided only for the adjusted model of the linear regression used to estimate the effect sizes.*
- *It is not possible to provide numerators and denominators for the percentages reported for analysis with multiple imputation. An explanation about this was included in each table legend as a response to a previous similar comment.*
- *The supplementary tables numbers mirror the main tables number with same content (with the addition of a letter). Therefore, there is no Supplementary table 2.*

**Comments from the reviewers:**

Reviewer #1: Thanks for including the information requested which as suspected has revealed that there is no single "standard care" in the different sites not randomised to GAP. This likely means that GAP has been compared to multiple different preexisting detection rates. However, I think as this information is now clear for the reader there is no reason for the manuscript to go forward for publication in my view and look forward to the interest and debate it will create.

**Response**

- *Thank you for reviewing our manuscript.*

Reviewer #2: I presume I've been asked to re-review (after commenting that I was happy with the R2 version) is to comment on the two-stage vs one-stage approach. I'm afraid I am not knowledgable about that: I asked a colleague who works on cluster randomised trials and they are not aware of any investigation that has shown an issue with two-stage approaches. They did not feel there was a reason to favour using a one-stage approach instead of a two-stage.

I think the authors having added an additional analysis and discussion has addressed this issue to my satisfaction, but the other reviewer perhaps knows more about the issue than I do.

**Response**

- *Thank you for reviewing our manuscript. We are pleased to hear you agree with our approach.*

Reviewer #4: I have no further suggestions for the manuscript; this revised version of the manuscript addresses my previous concerns.

**Response**

- *Thank you for reviewing our manuscript.*
